# Supplementary material for: What should be included in case report forms? Development and application of novel methods to inform surgical study design: a mixed methods case study in parastomal hernia prevention
Source: BMJ Open. 2022 Oct 5;12(10):e061300. doi: 10.1136/bmjopen-2022-061300 (PMC9535162; doi:10.1136/bmjopen-2022-061300)
Supplement: Supplementary data [file bmjopen-2022-061300supp001.pdf]

Additional File 1, Appendices

Table 1: Example from the long-list representing the discussion held at the consensus meeting on the overarching category theme '*Reinforcing the stoma trephine with mesh*'.

Technical variations		
Reinforcing the stoma trephine with mesh		
**a. Mesh type**	Synthetic	Non-absorbable [polypropylene, polyethylene terephthalate, polytetrafluoroethylen (PTFE), polyvinylidene fluoride (PVDF), other]
		Absorbable [rapid, delayed]
		Combined
	Biologic	
	Other	
**b. Mesh size**	Size of mesh inserted [height and width measured in cm OR if circular widest diameter in cm]	Mesh was uncut/unadjusted
		Mesh not cut/adjusted
	Mesh size estimation	Finger breadths
		Ruler
		Eye
		Other
**c. Shape of inserted mesh**	2D	Oval
		Square
		Circle
		Other
	3D	Funnel
		Other
**d. d. Location of mesh and mechanism of creating space**	Sublay [pre-peritoneal, retro-rectus]	Space created by instrumental dissection
		Space created with blunt force (e.g. finger)
		Combination
		Other
	Onlay [supra-rectus]	Space created by instrumental dissection
		Space created with blunt force (e.g. finger)
		Combination
		Other

<table>
<tr><td rowspan="2"></td><td colspan="3">Inlay [intra-rectus]</td></tr>
<tr><td colspan="3">Underlay [intra-peritoneal, IPOM]</td></tr>
<tr><td rowspan="4">e. Route used to position mesh</td><td colspan="3">Through the main operative incision</td></tr>
<tr><td colspan="3">Through the stoma trephine</td></tr>
<tr><td colspan="3">Via a port</td></tr>
<tr><td colspan="3">Other</td></tr>
<tr><td rowspan="9">f. Mesh trephine</td><td colspan="3">Sugarbaker (mesh edges secured to fascial edges)</td></tr>
<tr><td colspan="3">Modified Sugarbaker (mesh edges secured overlapping the fascial edges)</td></tr>
<tr><td rowspan="7">Key-hole (trephine within the mesh) Y/N</td><td colspan="2">Size of key-hole (widest diameter in cm)</td></tr>
<tr><td rowspan="3">Shape of key-hole</td><td>Circular</td></tr>
<tr><td>Cruciate</td></tr>
<tr><td>Other</td></tr>
<tr><td colspan="2">Key-hole created using a mechanical device (other than the tacker)</td></tr>
<tr><td colspan="2">Key-hole created before mesh has been secured</td></tr>
<tr><td colspan="2">Key-hole created after mesh has been secured</td></tr>
<tr><td rowspan="18">g. Securing the mesh</td><td colspan="3">Not secured</td></tr>
<tr><td rowspan="11">Securing the mesh to the abdominal wall</td><td rowspan="3">Tacking</td><td>Single crown</td></tr>
<tr><td>Double crown</td></tr>
<tr><td>Other</td></tr>
<tr><td rowspan="2">Suturing</td><td>Continuous</td></tr>
<tr><td>Interrupted [entire perimeter, corners only, other]</td></tr>
<tr><td rowspan="2">Suture choice</td><td>Absorbable</td></tr>
<tr><td>Non-absorbable</td></tr>
<tr><td>Glue</td><td></td></tr>
<tr><td colspan="2">Combination of tacks and sutures</td></tr>
<tr><td colspan="2">Mechanical device to secure the mesh (other than tacker)</td></tr>
<tr><td colspan="2">Other</td></tr>
<tr><td rowspan="6">Securing the mesh to the stoma serosa</td><td rowspan="3">Suturing</td><td>Interrupted [number and position]</td></tr>
<tr><td>Continuous</td></tr>
<tr><td>Other</td></tr>
<tr><td rowspan="2">Suture choice</td><td>Absorbable</td></tr>
<tr><td>Non-absorbable</td></tr>
<tr><td colspan="2">Other</td></tr>
</table>

Table 2: Examples from the qualitative data representing the development of the overarching category theme '*Trephine formation: Skin and subcutaneous tissue; Muscle layers; Other*

**Overarching category theme**<br><br>**Trephine formation*: Skin and subcutaneous tissue***	
**Sub theme**	**Extract**
Skin incision at stoma site	*Extract 1: "The surgeon turns the knife 90 degrees and 'shaves through' the skin around the Langenbeck. This makes a circle shaped incision in the skin. They then achieve haemostasis using forceps diathermy." [Observation NB: BRI0021, End Colostomy, Laparoscopic, RDE]*<br><br>*Extract 2: "Holding the skin edges with some forceps, the surgeon uses diathermy to incise each edge (skin and subcutaneous tissue), thereby turning the cruciate incision into a oval." [Observation NB: BRI0034, End Ileostomy, Converted laparoscopic to open, BRI]*
Subcutaneous adipose	*Extract 3: "I used to take out a long column of fat and then I thought, "Well, fat is so compressible, what's the point?" And so I just separate it and cut, you know, in a straight line down to the rectus with some protractors… Well, I'm trying to take away- take away as little as possible, to try and minimise the risk of parastomal hernias. So I don't know if it works or not, but again, it's anecdotal other than evidence-based." [HCP: BRI0009, Surgeon, Upper GI, BRI]*<br><br>*Extract 4: Interviewer: "So you think that may have an effect (on PSH rates)."*<br><br>*Respondent:" Yes…And I think the fatter somebody is the more fat people excise and therefore the bigger the space because the fat retracts around it." [HCP: BRI0015, Surgeon, Lower GI, RDE]*<br><br>*Extract 5: "I would have a cone of fat with a circle of skin on the top." [HCP: BRI0018, Surgeon, Hepatobiliary, RDE]*<br><br>*Extract 6: "No subcutaneous tissue is excised." [Observation CM; RDE, End Colostomy, Laparoscopic, RDE]*

**Overarching category theme**<br>**Trephine formation*: Muscle layers***	
**Sub theme**	**Extract**
Location of the muscle trephine	*Extract 7: "I think it makes a difference [to PSH rates]. If you put the trephine within the rectus sheath then the rectus sheath is a closed compartment because you've got a posterior and anterior layer and lateral and medial. Therefore any rise in the force or the pressure within that rectus abdominal muscle by the passage of stoma, or material through a stoma, is equalised by the fact it's a closed compartment so you get are resistant force. If you put it outside the rectus abdominus, in the obliques, then by the very nature of which your obliques and transverse salus separate then they're more likely to separate around a stoma. I think there's a difference in whether the patient's got a posterior sheath or whether you've got a peritoneum because if you've only got a peritoneum you're less likely to have a resisted force because it's dissipated by the peritoneum at the back as opposed to posterior sheath."* [HCP: BRI0015, Surgeon, Lower GI, RDE]<br><br>*Extract 8: "I honestly don't know. You could imagine it might [make a difference in PSH rates], but I don't know."* [HCP: BRI0018, Surgeon, Hepatobillary, RDE]<br><br>*Extract 9: "Patients who have [a stoma] above the arcuate line of Douglas may be less inclined to have parastomal hernias…Disrupting the linea semilunaris I think is a recipe for disaster."* [HCP: BRI0023, Lower GI, RDE]
Approach to creating the trephine through the anterior sheath	*Extract10: "Some people do transverse. Some do longitudinally. Some make cruciate incisions. I've seen people do incisions say either longitudinally or horizontally, and then buttress the ends with suture material, to try and prevent propagation." [HCP: BRI0023, Lower GI, RDE]*<br><br>*Extract 11: 'By virtue of doing a cruciate incision you are splitting, because you've got a linear incision, it might widen. If we make a circular incision does that mean that the tensal strength or the*

	forces within the abdominal wall, are they going to distribute." [HCP: BRI0004, Surgeon Lower GI, BRI]<br><br>*Extract 12 "The cruciate incision allows the bowel to come through with the minimum defect. So that's why I've avoided doing the circular incision in the rectus sheath." [HCP: BRI0009, Surgeon, Upper GI, BRI]*
Approach to creating the trephine through the posterior sheath/peritoneum	*Extract 13: ""In terms of technique, I think the size of the cruciate incision, both on the anterior and the posterior layers of the rectus sheath, matter. I think that that's probably, I think, what matters most." [HCP: BRI0001, Surgeon, Upper GI, BRI]*
Approach to creating the muscle trephine	*Extract 14: "Respondent: If you de-vascularise any muscle fibres and it becomes infected then infection leads to hernia formation there is no doubt about that.*<br><br>*Interviewer: So splitting of the fibres improves (PSH rates) that rather than incising them?*<br><br>*Respondent: I think it does, I can't show you evidence to say that. Logically to me if you are splitting the fibres and not disrupting my cutting through the fibres and all the blood supply to the muscle and the surrounding area." [HCP: BRI0022, Surgeon, Lower GI, RDE]*
Dilation of the stoma	*Extract 15: "Further fibres of muscles/fascial tissue are cut with diathermy" [Observation NB: BRI0021, End Colostomy, Laparoscopic, RDE]*
How the measurements of the stoma trephine are made	*Extract 16: "Well, it's very subjective. Some people would say two fingers, but my two fingers are bigger than your two fingers." [HCP: BRI0010, Surgeon, Lower GI, BRI]*
**Overarching category theme**<br><br>**Trephine formation*: Other***	

**Sub theme**	**Extract**
Intraoperative nerve damage	*Extract 17: "I think if you start making incisions I think you're more at risk at damaging muscles- Not muscles, the nerves that run into the abdominal wall." [HCP: BRI0004, Surgeon Lower GI, BRI]*

Table 3: Examples from the qualitative data representing the development of the overarching category theme '*reinforcing the stoma trephine with mesh'*

***Overarching category theme: 'R*einforcing the stoma trephine with mesh'**	
**Sub theme**	**Extract**
Mesh type	*Extract 6: "See, the fact that all these different meshes are being used for parastomal hernias must mean that no one of them is actually working. The more ways there are of doing something, the more sure you are that nobody knows what the right way is." [HCP: BRI0009, Upper GI, BRI]*<br><br>*Extract 7: "The corners are trimmed off the mesh, making an oval shape. It looks like it's made of polypropylene."* [Observation NB: BRI0021, End Colostomy, Laparoscopic, RDE]<br><br>*Extract 8: Interviewer: "Why do you think Permacol's [biologic mesh] beneficial?"*<br><br>*Respondent: "Compared to [synthetic] mesh? Just because it resists infection better."* [HCP: BRI0032, Lower GI, BRI]<br><br>*Extract 9: "I would use in most patients a piece of polypropylene mesh in the retrorectus position…With the synthetic meshes the rate of tissue ingrowth is so rapid, and scar tissue formation is so quick, that you will find that you will have a fairly solid ring of tissue within four to six weeks, so that you get the protective effect relatively quickly. It's why most of the RCTs would demonstrate nowadays a protective effect with prophylactic mesh."* [HCP: BRI0023, Lower GI, RDE]
Mesh size	*Extract 10: "The consultant measures (using a ruler) the width and length of the retrorectus space and draws a rectangle in permanent marker of the edge of the mesh. The SpR holds the mesh while the consultant cuts*

	using scissors." [Observation CM: BRI0030, End Colostomy, Open, BRI]<br><br>*Extract 12: "If we do that it involves developing a plane between the muscle layer and the posterior sheath and placing a very small piece of mesh 4cm by 4cm with polypropylene which you pull the stoma through."* [HCP: BRI0022, Lower GI, RDE]
Mesh shape	*Extract 13: "The corners are trimmed off the mesh, making an oval shape. It looks like it's made of polypropylene…The surgeon folds the mesh into quarters, then cuts off the corner, which makes a circular shaped hole in the centre.* [Observation NB: BRI0021, End Colostomy, Laparoscopic, RDE]
Location of mesh	*Extract 14: "The consultant then places the mesh with the retrorectus space."* [Observation CM: BRI0030, End Colostomy, Open, BRI]<br><br>*Extract 15: "Whether that is done intra-peritoneal, whether that's done in a retroperitoneal space, or in an onlay space… So, what I'm saying to you is there's about ten different ways of doing something, which to anyone says that none of them are perfect, and that's never really been subjected to a trial of sufficient power, strength for us to know what the most effective technique is."* [HCP: BRI0004, Lower GI, BRI]<br><br>*Extract 16: "I think the evidence when they did all those prophylactic meshes, whether you put it intraperitoneal, retromuscular or anterior into the muscles, it didn't show any difference in the outcomes. I think it's just strengthening the muscle layer, but probably a large trial will tell us whether there's any difference. In an ideal world, I think you should probably put it either intraperitoneal or retromuscular. I think putting it anterior to the rectus sheath is probably pointless, you might as well not put it, I don't think it adds anything to it because the hernias start from the inside…Yes, it will just lift the mesh; it's not going to make any difference. It either has to be between the rectus and the posterior sheath or intraperitoneal where it might have a use."* [HCP: BRI0010, Lower GI, BRI]

How the space is created and method of positioning the mesh	*Extract 17: "Single- index finger feels and sweeps around breath of trephine to create a space in the retrorectus space."* [Observation CM: BRI0021, End Colostomy, Laparoscopic, RDE]<br><br>*Extract 18 "The surgeon uses their finger to create a space, and further fibres of muscles/fascial tissue is cut with diathermy…The surgeon uses a clip to guide the mesh into the pocket that they have created for it. They use their fingers to smooth the mesh out and get it into position."* [Observation NB: BRI0021, End Colostomy, Laparoscopic, RDE]
Mesh trephine	*Extract 19: "But I think, if you're putting a keyhole into any mesh, in mesh prophylaxis, or even as a repair, it's just going to shrink away, that circle is just going to get larger…*<br><br>*Interviewer: "So it contracts… Do you think that's a reason why the Sugarbaker technique would be superior to-"*<br><br>*Respondent: "Yes, so that's what I think, why Sugarbaker would be superior. That said, I don't think it's 100% effective. That's what I would use as a method of mesh prophylaxis and permanent stoma."* [HCP: BRI004, Lower GI, BRI]<br><br>*Extract 20: "If you've got a circular incision maybe you've got a bit more strength around the radius of it, rather than splitting it."* [HCP: BRI0032, Surgeon Lower GI, RDE]
Method of securing the mesh	*Extract 21: 'When I do it laparoscopically I just use the tacks. If I'm doing open surgery I use stitches."* [HCP: BRI0022, Lower GI, RDE]<br><br>*Extract 22: I will use transfascial sutures to place the mesh as well, and that acts partially as a fixation. I will do that and some stapling device as well, and the Sugarbaker is associated with its own unique stapling pattern, because of the way in which it overlies the bowel."* [HCP: BRI023, Lower GI, RDE]

Table 4: Short-list of data items used for development of the CIPHER Study case report forms

<table>
<tr><th colspan="3">1. Surgical approach to stoma formation</th></tr>
<tr><td rowspan="2">a. Context of stoma formation</td><td colspan="2">Indication for surgery (see separate sheet)</td></tr>
<tr><td colspan="2">Name of procedure (see separate sheet)</td></tr>
<tr><td rowspan="3">b. Intended type of access used</td><td colspan="2">Minimally invasive [SILS; Laparoscopic; Robotic]</td></tr>
<tr><td colspan="2">Open</td></tr>
<tr><td colspan="2">Trephine</td></tr>
<tr><td>c. Intended type of procedure converted to open</td><td colspan="2">Conversion from minimally invasive to open [SIL converted, Laparoscopic converted; Robotic converted] Y/N</td></tr>
<tr><td rowspan="6">d. Type of stoma formed</td><td colspan="2">Envisaged longevity of stoma [permanent, uncertain]</td></tr>
<tr><td colspan="2">End</td></tr>
<tr><td>Loop</td><td>With or without rod</td></tr>
<tr><td colspan="2">Loop end (Abcarian)</td></tr>
<tr><td colspan="2">Double barrelled</td></tr>
<tr><td colspan="2">Other</td></tr>
<tr><td rowspan="6">e. Section of bowel used to form functioning end of stoma</td><td colspan="2">Jejunum</td></tr>
<tr><td colspan="2">Ileum</td></tr>
<tr><td colspan="2">Ascending colon</td></tr>
<tr><td colspan="2">Transverse colon</td></tr>
<tr><td colspan="2">Descending colon</td></tr>
<tr><td colspan="2">Sigmoid colon</td></tr>
<tr><td rowspan="2">f. Stoma site</td><td rowspan="2">Stoma site pre-marked Y/N [preserved with suture, pen or not preserved]</td><td>Stoma site marked by [stoma nurse, surgeon, non-specialist nurse, other]</td></tr>
<tr><td>Stoma formed/ not formed at pre-marked site (Y/N)</td></tr>
<tr><td rowspan="2">g. Route of stoma</td><td colspan="2">Trans-peritoneal</td></tr>
<tr><td colspan="2">Extra-peritoneal (cannot be trephine in section b)</td></tr>
<tr><th colspan="3">2. Trephine formation</th></tr>
<tr><td>a. Subcutaneous tissue</td><td colspan="2">Subcutaneous tissue excised (Y/N)</td></tr>
<tr><td rowspan="3">b. Relationship of the muscle layer incision to the rectus abdominis</td><td colspan="2">Outside of the rectus sheath (Within oblique abdominal muscles)</td></tr>
<tr><td rowspan="2">Within the rectus sheath</td><td>Through the belly of the rectus abdominis</td></tr>
<tr><td>Lateral to the belly of the rectus abdominis* (e.g. LRAPS technique)</td></tr>
<tr><td rowspan="5">c. Anterior sheath (ONLY if within the rectus sheath*)</td><td colspan="2">Was a laparoscopic trocar used to puncture the anterior sheath (Y/N) (only for minimally invasive Laparoscopic + robotic OR converted Laparoscopic + robotic procedure) (NOT SILS or SILS converted)</td></tr>
<tr><td colspan="2">Size of incision [widest diameter in mm]</td></tr>
<tr><td colspan="2">Shape of incision [linear (horizontal, vertical), cruciate, circular, other]</td></tr>
<tr><td colspan="2">Was any of the anterior sheath removed? (Y/N)</td></tr>
<tr><td>Adjustments made to the size of the incision (Y/N)</td><td>Sutures used to buttress end of incision (Y/N)</td></tr>
</table>

<table>
<tr><td rowspan="5">d. Posterior sheath (ONLY within the rectus sheath*)</td><td colspan="3">Was a laparoscopic trocar used to puncture the anterior sheath (Y/N) (only for minimally invasive Laparoscopic + robotic OR converted Laparoscopic + robotic procedure) (NOT SILS or SILS converted)</td></tr>
<tr><td colspan="3">Size of incision [widest diameter in mm]</td></tr>
<tr><td colspan="3">Shape of incision [linear (horizontal, vertical), cruciate, circular, other]</td></tr>
<tr><td colspan="3">Was any of the posterior sheath removed? (Y/N)</td></tr>
<tr><td colspan="2">Adjustments made to the size of the incision (Y/N)</td><td>Sutures used to buttress end of incision (Y/N)</td></tr>
<tr><td>e. Muscle fibres</td><td colspan="3">Separated with blunt dissection (Y/N) (only possible if within rectus belly/oblique muscles)</td></tr>
<tr><td>f. Intra-operative vessel damage</td><td colspan="3">Epigastric vessel (Y/N)</td></tr>
<tr><td rowspan="2">g. Laparoscopic procedures (only for minimally invasive Laparoscopic + robotic OR converted Laparoscopic + robotic procedure) (NOT SILS)</td><td rowspan="2">Location of trephine in relation to port site [Trephine created at port site/ Trephine created in a location other than port site]</td><td rowspan="2">Trephine created at port site</td><td>Trephine created at the port site at the beginning of procedure [then subsequently used as port site]</td></tr>
<tr><td>Trephine created at end of procedure [conversion of port site to trephine]</td></tr>
<tr><td colspan="4">3. Reinforcing the stoma trephine with mesh</td></tr>
<tr><td>a. Mesh type</td><td colspan="2">Mesh used (Y/N)</td><td>Product code, fix label, provide manufacture name</td></tr>
<tr><td rowspan="2">b. Mesh size</td><td rowspan="2" colspan="2">Mesh cut or adjusted (Y/N)</td><td>Size of mesh inserted if changed from original (Y/N) [height and width measured in cm OR if circular/oval diameter in cm]</td></tr>
<tr><td>Shape of mesh if changed from original (Y/N) [3D/funnel; Circular/oval; Square/rectangular]</td></tr>
<tr><td>c. Location of mesh placement</td><td colspan="3">Please indicate using diagram [Diagram provided with the following drop-down categories: Sublay/pre-peritoneal/retro-rectus; Underlay/intra-peritoneal; Onlay; Inlay] (Retrorectus; Intra-peritoneal; Onlay but keep all descriptions on the diagram)</td></tr>
<tr><td rowspan="5">d. Route used to position mesh</td><td colspan="3">Through the main operative incision (e.g. in an open procedure or an extended port site) (any type of surgery) (can be all three: onlay, intraperitoneal or retorectus)</td></tr>
<tr><td colspan="3">Through the stoma trephine (any type of surgery) (can be all three: onlay, intraperitoneal or retorectus)</td></tr>
<tr><td colspan="3">Via a port (Any minimally invasive or converted procedure) (can only be intraperitoneal placement of mesh)</td></tr>
<tr><td rowspan="2" colspan="2">Keyhole Y/N</td><td>What shape was the keyhole [cruciate; circular/oval; Slit]</td></tr>
<tr><td>What was the size was the keyhole (Maximum diameter)</td></tr>
</table>

**e. Securing the mesh**	Mesh secured to abdominal wall *(including sheath, muscle, peritoneum)* (Y/N)
	Mesh secured to stoma serosa (Y/N)
**4. Use of the stoma as a specimen extraction site**	
**a. Stoma trephine used as a specimen extraction site**	Stoma trephine used as an extraction site (Y/N)
**5. Closure of other wounds formed during the procedure**	
**a. Closure of deep layers of the abdominal wall**	Main abdominal incision (e.g. in an open procedure, extended port site or specimen extraction site) [Small bite closure; Large bite closure; NA])
	Biggest port site (e.g. 10, 11 or 12mm) *(Minimally invasive laparoscopic + robotic OR converted laparoscopic + robotic procedures only)* [Closure of deep layer Y/N/NA]
**6. Spouting the stoma lumen**	
**a. Suturing of bowel to skin**	Has the stoma been spouted (Y/N)
