# Supplementary material for: What should be included in case report forms? Development and application of novel methods to inform surgical study design: a mixed methods case study in parastomal hernia prevention
Source: BMJ Open. 2022 Oct 5;12(10):e061300. doi: 10.1136/bmjopen-2022-061300 (PMC9535162; doi:10.1136/bmjopen-2022-061300)
Supplement: Supplementary data [file bmjopen-2022-061300supp002.pdf]

CIPHER STUDY

The CIPHER Study

SURGICAL TECHNIQUE

TH1

Patient Initials: ☐☐☐

CIPHER Study ID: ☐☐☐-☐☐☐☐

SURGEON DETAILS

Most senior surgeon scrubbed in at time of stoma formation:

Name: ____________________ GMC number: ____________________

PATIENT DETAILS

Operation date: __ __ /__ __ /__ __ __ __ (d d / m m / y y y y)

Operation start time
(defined as knife to skin: time): __ __ : __ __

Operation end time
(defined as time of final skin suture in wound): __ __ : __ __

Month of birth: __ __ Year of birth: __ __ __ __

Patient's Sex: *Male* ☐ *Female* ☐

SURGICAL APPROACH TO STOMA FORMATION

Indication for surgery (***please select one option***):

- Tumour – benign ☐
- Tumour – malignant ☐
- Diverticular Disease ☐
- Functional Intestinal Disorder ☐
- Inflammatory Bowel Disease (IBD) ☐
- IBD – Crohn's ☐
- IBD – Ulcerative Colitis ☐
- Other ☐

If other, specify: ____________________

ASA Classification:

- I ☐
- II ☐
- III ☐
- IV ☐
- V ☐

2

CIPHER STUDY

The CIPHER Study

SURGICAL TECHNIQUE

TH1

Patient Initials: ☐☐☐

CIPHER Study ID: ☐☐☐-☐☐☐☐

Name of procedures (tick YES or NO as appropriate for each):

	Yes	*No*
Small bowel resection	☐	☐
Colectomy: left (including sigmoid colectomy and anterior resection)	☐	☐
Colectomy: right (including ileocaecal resection)	☐	☐
Colectomy: subtotal or panproctocolectomy	☐	☐
Hartmann's procedure	☐	☐
Colorectal resection – other	☐	☐
Reduction of volvulus	☐	☐
Stricturoplasty	☐	☐
Drainage of abscess/collection	☐	☐
Debridement	☐	☐
Abdominoperineal excision	☐	☐
Posterior pelvic exenteration	☐	☐
Repair or revision of anastomosis	☐	☐
Repair of intestinal fistula	☐	☐
Resection of other intra-abdominal tumour(s)	☐	☐
Stoma formation	☐	☐
Other	☐	☐

If other, specify: ____

If **YES** to ***abdominoperineal excision*** or ***posterior pelvic exenteration***:

Vertical rectus abdominis myocutaneous (VRAM) flap *Yes* ☐ *No* ☐

Intended type of access used (***please select one***):

- SLS ☐
- Laparoscopic ☐
- Robotic ☐
- Open ☐
- Trephine ☐

Intended type of procedure converted to open: *Yes* ☐ *No* ☐
***(Do not answer if intended type of access* was open)**

3

TH1

The CIPHER Study

SURGICAL TECHNIQUE

Patient Initials: ☐☐☐

CIPHER Study ID: ☐☐☐-☐☐☐☐

Envisaged longevity of stoma: Permanent ☐ Uncertain ☐

Type of stoma formed (***please select one***):

- End ☐
- Loop ☐ → If **loop**, with or without rod: With ☐ Without ☐
- Loop end ☐
- Double barrelled ☐
- Other ☐

Section of bowel used to form functioning end of stoma (***please select one***):

- Jejunum ☐
- Ileum ☐
- Ascending colon ☐
- Transverse colon ☐
- Descending colon ☐
- Sigmoid colon ☐

Stoma site pre-marked (***please select one***):

- Not preserved ☐
- Preserved with suture ☐ →
- Pre-marked with pen ☐ →

If ***preserved with suture*** or ***pre-marked with pen***,
Stoma site marked by (***please select one***):

- Stoma nurse ☐
- Surgeon ☐
- Non-specialist nurse ☐
- Other ☐

If other, specify: _______________

	Yes	*No*
Stoma formed at pre-marked site:	☐	☐

Route of stoma: Trans-peritoneal ☐ Extra-peritoneal ☐

4

TH1

The CIPHER Study

SURGICAL TECHNIQUE

Patient Initials: ☐☐☐

CIPHER Study ID: ☐☐☐-☐☐☐☐

TREPHINE FORMATION

Subcutaneous tissue excised: *Yes* ☐ *No* ☐

Relationship of the muscle layer incision to the rectus abdominis (***please select one***):

- Outside of the rectus sheath (within oblique abdominal muscles) ☐ → **Continue on next page**
- Within the rectus sheath - though the belly of the rectus abdominis ☐
- Within the rectus sheath - lateral to the belly of the rectus abdominis ☐

Complete the box below

Anterior sheath: was a laparoscopic trocar used to puncture the anterior sheath *Yes* ☐ *No* ☐
(Only answer for laparoscopic or robotic procedures)

Anterior sheath: size of incision [widest diameter in mm] ___________

Anterior sheath: Shape of incision (***please select one***)

- Linear - horizontal — ☐
- Linear - vertical | ☐
- Cruciate + ☐
- Circular ○ ☐
- Other ? ☐

Anterior sheath: was any of the anterior sheath removed *Yes* ☐ *No* ☐

Anterior sheath: adjustments made to the size of the incision *Yes* ☐ *No* ☐

Posterior sheath: was a laparoscopic trocar used to puncture the posterior sheath *Yes* ☐ *No* ☐
(Only answer for laparoscopic or robotic procedures)

Posterior sheath: size of incision [widest diameter in mm] ___________

Posterior sheath: Shape of incision (***please select one***)

- Linear - horizontal — ☐
- Linear - vertical | ☐
- Cruciate + ☐
- Circular ○ ☐
- Other ? ☐

Posterior sheath: was any of the posterior sheath removed *Yes* ☐ *No* ☐

Posterior sheath: adjustments made to the size of the incision *Yes* ☐ *No* ☐

Murkin C, *et al. BMJ Open* 2023; 12:e061300. doi: 10.1136/bmjopen-2022-061300

The CIPHER Study

TH1

SURGICAL TECHNIQUE

Patient Initials: ☐☐☐

CIPHER Study ID: ☐☐☐-☐☐☐☐

Muscle fibres separated with blunt dissection *Yes* ☐ *No* ☐

Intra-operative vessel damage - epigastric vessel *Yes* ☐ *No* ☐

Location of trephine in relation to port site (***please select one***):
(Only answer for laparoscopic or robotic procedures, **including procedures converted to open)**

- Trephine created at the port site as the beginning of procedure (then subsequently used as port site) ☐
- Trephine created at end of procedure (conversion of port site to trephine) ☐
- Trephine created in a location other than port site ☐

Were sutures used to buttress the incision (***please select one***):
(Only answer if relationship of the muscle layer incision to the rectus abdominis is "within rectus sheath")

- Anterior sheath only ☐
- Posterior sheath only ☐
- Both anterior and posterior sheath ☐
- Anterior and posterior sheath sutured together ☐
- No ☐

6

The CIPHER Study

SURGICAL TECHNIQUE

TH1

Patient Initials: ☐☐☐

CIPHER Study ID: ☐☐☐-☐☐☐☐

REINFORCING THE STOMA TREPHINE WITH MESH

Was mesh used to reinforce the stoma trephine *Yes* ☐ *No* ☐ → If **NO**, continue on next page

If **YES**, complete the box below

Mesh product code (attach product sticker) ***Attach product sticker here***

Mesh cut or adjusted *Yes* ☐ *No* ☐

If **YES**,

Craniocaudal length of mesh inserted if changed from original [in cm] ______________

Medio-lateral length of mesh inserted if changed from original [in cm] ______________

Diameter of mesh inserted if changed from original [in cm] ______________

Shape of mesh inserted if changes from original (***please select one***):

- 3D/funnel ☐
- Circular/Oval ☐
- Square/rectangular ☐
- No change in shape ☐

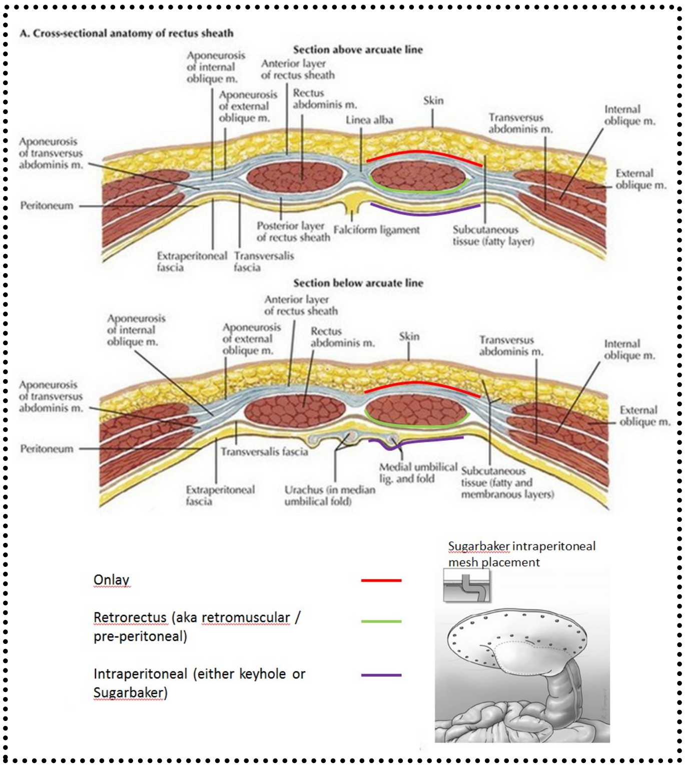

Location of mesh replacement (***please select one***):

- Sublay/pre-peritoneal/retro-rectus ☐
- Underlay/intra-peritoneal ☐
- Onlay ☐

Route used to position mesh (***please select one***):

- Through the main operative incision ☐
- Through the stoma trephine ☐
- Via a port ☐

What shape was the hole in the mesh?

Cruciate ☐ Circular/oval ☐ Slit ☐ None (Sugarbaker) ☐

What size was the hole in the mesh [in mm] ___________

Mesh secured to abdominal wall (including sheath, muscle, peritoneum) *Yes* ☐ *No* ☐

Mesh secured to stoma serosa *Yes* ☐ *No* ☐

Murkin C, *et al. BMJ Open* 2023; 12:e061300. doi: 10.1136/bmjopen-2022-061300

TH1

The CIPHER Study

SURGICAL TECHNIQUE

Patient Initials: ☐☐☐

CIPHER Study ID: ☐☐☐-☐☐☐☐

USE OF THE STOMA TREPHINE AS A SPECIMEN EXTRACTION SITE

Stoma trephine used as an extraction site *Yes* ☐ *No* ☐

CLOSURE OF OTHER WOUNDS FORMED DURING THE PROCEDURE

Main abdominal incision (***please select one***):

Small bite closure ☐

Large bite closure ☐

N/A ☐

Biggest port size [in mm] __________
(Only answer for laparoscopic or robotic procedures, **including procedures converted to open)**

Closure of deep layer *Yes* ☐ *No* ☐
(Only answer for laparoscopic or robotic procedures, **including proce- dures con- verted to open)**

SPOUTING THE STOMA LUMEN

Has the stoma been spouted *Yes* ☐ *No* ☐

COMMENTS

____

____

____

____

Who has collated this data?

GMC/NMC number: ________________________

Date completed: __ __ / __ __ /__ __ __ __
