# Supplementary material for: What should be included in case report forms? Development and application of novel methods to inform surgical study design: a mixed methods case study in parastomal hernia prevention
Source: BMJ Open. 2022 Oct 5;12(10):e061300. doi: 10.1136/bmjopen-2022-061300 (PMC9535162; doi:10.1136/bmjopen-2022-061300)
Supplement: Author's manuscript [file bmjopen-2022-061300.draft_revisions.pdf]

BMJ Open is committed to open peer review. As part of this commitment we make the peer review history of every article we publish publicly available.

When an article is published we post the peer reviewers' comments and the authors' responses online. We also post the versions of the paper that were used during peer review. These are the versions that the peer review comments apply to.

The versions of the paper that follow are the versions that were submitted during the peer review process. They are not the versions of record or the final published versions. They should not be cited or distributed as the published version of this manuscript.

BMJ Open is an open access journal and the full, final, typeset and author-corrected version of record of the manuscript is available on our site with no access controls, subscription charges or pay-per-view fees (http://bmjopen.bmj.com).

If you have any questions on BMJ Open's open peer review process please email info.bmjopen@bmj.com

Development and application of novel methods to inform surgical study design and case report forms: a case study in parastomal hernia prevention

Journal:	*BMJ Open*
Manuscript ID	bmjopen-2022-061300
Article Type:	Original research
Date Submitted by the Author:	25-Jan-2022

Complete List of Authors:	Murkin, Charlotte; Bristol Medical School, Population Health Sciences<br>Rooshenas, Leila; University of Bristol, School of Social and Community Medicine<br>Smart , Neil ; Royal Devon & Exeter NHS Foundation Trust<br>Daniels, IR; Royal Devon & Exeter Hospital<br>Pinkney, Tom; University of Birmingham School of Clinical and Experimental Medicine, Surgery<br>Shabbir, J; University Hospitals Bristol NHS Foundation Trust<br>Rockall, Timothy; The Royal Surrey County Hospital, Oesophago-gastric Surgery<br>Bennett, J; Gloucestershire Hospitals NHS Foundation Trust, Department of Colorectal Surgery<br>Torkington, Jared; University Hospital of Wales, Department of Colorectal Surgery<br>Randall, J; University Hospitals Bristol NHS Foundation Trust<br>Brandsma, HT; Canisius Wilhelmina Hospital, Department of Surgery<br>Reeves, Barnaby; University of Bristol, Bristol Heart Institute<br>Blazeby, Jane; University of Bristol, Department of Social Medicine<br>Blencowe, Natalie; Bristol Medical School, Population Health Sciences

Keywords:	QUALITATIVE RESEARCH, SURGERY, EPIDEMIOLOGY

SCHOLARONE™
Manuscripts

I, the Submitting Author has the right to grant and does grant on behalf of all authors of the Work (as defined in the below author licence), an exclusive licence and/or a non-exclusive licence for contributions from authors who are: i) UK Crown employees; ii) where BMJ has agreed a CC-BY licence shall apply, and/or iii) in accordance with the terms applicable for US Federal Government officers or employees acting as part of their official duties; on a worldwide, perpetual, irrevocable, royalty-free basis to BMJ Publishing Group Ltd ("BMJ") its licensees and where the relevant Journal is co-owned by BMJ to the co-owners of the Journal, to publish the Work in this journal and any other BMJ products and to exploit all rights, as set out in our licence.

The Submitting Author accepts and understands that any supply made under these terms is made by BMJ to the Submitting Author unless you are acting as an employee on behalf of your employer or a postgraduate student of an affiliated institution which is paying any applicable article publishing charge ("APC") for Open Access articles. Where the Submitting Author wishes to make the Work available on an Open Access basis (and intends to pay the relevant APC), the terms of reuse of such Open Access shall be governed by a Creative Commons licence – details of these licences and which Creative Commons licence will apply to this Work are set out in our licence referred to above.

Other than as permitted in any relevant BMJ Author's Self Archiving Policies, I confirm this Work has not been accepted for publication elsewhere, is not being considered for publication elsewhere and does not duplicate material already published. I confirm all authors consent to publication of this Work and authorise the granting of this licence.

For peer review only

Development and application of novel methods to inform surgical study design and case report forms: a case study in parastomal hernia prevention

C Murkin[1], L Rooshenas[1], NJ Smart[2], IR Daniels[2], T Pinkney[3], J Shabbir[4], T Rockall[5], J Bennett[6], J Torkington[7], J Randall[4], HT Brandsma[8], B Reeves[9], JM Blazeby[1], NS Blencowe[1]

On behalf of the CIPHER Study[9]

1. Bristol NIHR Biomedical Research Centre and Centre for Surgical Research, Population Health Sciences, Bristol Medical School, Bristol, UK
2. Exeter Surgical Health Services Research Unit (HeSRU), Royal Devon & Exeter Hospital, Exeter, Devon, UK
3. Academic Department of Surgery, Queen Elizabeth Hospital, University of Birmingham, UK
4. Division of Surgery, University Hospitals Bristol NHS Foundation Trust, Bristol, UK
5. Royal Surrey County Hospital NHS Foundation Trust, Guildford, UK
6. Department of Colorectal Surgery, Gloucestershire Hospitals NHS Foundation Trust, UK
7. Department of Colorectal Surgery, University Hospital of Wales Healthcare NHS Trust, Cardiff, UK
8. Department of Surgery, Canisius Wilhelmina Hospital, Nijmegen, The Netherlands
9. Clinical Trials and Evaluation Unit, Bristol Trials Centre, Bristol Medical School, University of Bristol, Bristol, UK

Corresponding author

NS Blencowe

Centre for Surgical Research

Population Health Sciences

Bristol Medical School

University of Bristol

natalie.blencowe@bristol.ac.uk

Keywords

Case report forms, cohort study, mixed methods research, qualitative research, literature reviews

For peer review only

Abstract

Objectives: To describe the development and application of methods to optimise the design of case report forms (CRFs) for clinical studies evaluating surgical procedures, illustrated with an example of abdominal stoma formation.

Design: i) Literature reviews, to identify reported variations in surgical components of stoma formation, were supplemented by ii) intra-operative qualitative research (observations, videos and interviews), to identify unreported variations used in practice to generate iii) a 'long list' of items, which were rationalised using iv) consensus methods, providing a pragmatic list of CRF items to be captured in the CIPHER study (Cohort study to Investigate the Prevention of parastomal HERnias).

Setting: Two secondary care surgical centres in England.

Participants: Patients undergoing stoma formation, surgeons undertaking stoma formation, and stoma nurses.

Outcome measures: Successful identification of key CRF items to be captured in the CIPHER study.

Results: 59 data items relating to stoma formation were identified and categorised within six themes: i) surgical approach to stoma formation; ii) trephine formation; iii) reinforcing the stoma trephine with mesh; iv) use of the stoma as a specimen extraction site; v) closure of other wounds during the procedure; and vi) spouting the stoma.

Conclusions: This study used multi-modal data collection to understand and capture the technical variations in stoma formation and design bespoke CRFs for a multicentre cohort study. The CIPHER study will use the CRFs to examine associations between the technical variations in stoma formation and risks of developing a parastomal hernia.

Clinical trial registration

Main CIPHER study ISRCTN17573805.

For peer review only

Article Summary

Strengths and limitations of this study

- To our knowledge, this is the first study to use a scientific and systematic process to inform the design of case report forms for a main study
- Supplementing literature reviews with qualitative methods helped to highlight important technique variations that would otherwise not have been identified
- However, this study was time consuming and future studies are needed to streamline the process of data collection and analysis

For peer review only

Introduction

A major challenge facing surgical research is that surgical procedures are considered to be complex healthcare interventions, comprising multiple components that can act independently or interdependently to influence outcomes (1). This complexity means that even surgical procedures labelled with the same name are frequently undertaken in different ways. Such variation has implications for the design and conduct of studies evaluating surgical interventions. It can introduce bias into randomised controlled trials (RCTs) due to difficulties in establishing exactly how the interventions in each group were delivered. In non-randomised studies, a lack of consideration for variations in surgical technique may compromise the full exploration of relationships between exposures and outcomes and result in misidentification of important factors, leading to criticism of the results (1). This may be particularly problematic in studies that aim to identify risk factors for the 'failure' of a procedure (i.e. recurrence of the same condition, or development of a post-operative complication).

One way to understand the heterogeneity of surgical procedures (within randomised and non-randomised studies) is to record accurately how their constituent components are performed, within case report forms (CRFs). This makes it possible to document variation in delivery of an intervention, and subsequently link this to outcomes, enabling study findings to be contextualised within routine practice. It is therefore crucial that consideration is given to how details of intervention delivery are recorded in CRFs. Whilst it may be impractical to monitor all components of an intervention, it is necessary to provide clarity about which details are expected to be documented and very important information for interpreting study results. Currently, however, methods for identifying the components of interest are lacking and there is a tension between recording all possible surgical components and streamlining data collection. This paper describes the development of novel methods to optimise the documentation of intervention components in CRFs for studies evaluating surgical procedures.

Methods

The research described in this paper was undertaken as preliminary work prior to the CIPHER study (UK Cohort study to Investigate the Prevention of parastomal HERnia), a multicentre cohort study (HTA-14/166/01) aiming to investigate the technical risk factors for developing parastomal hernia (PSH). Technical risk factors can be defined as variations in operative technique that may predispose patients to a PSH. PSH is a common complication following stoma formation surgery and although

For peer review only

various patient-related risk factors have been proposed, technical factors relating to the initial surgical procedure are much less certain. The preliminary work comprised three phases: 1) identification of all of the possible technical factors that may contribute to PSH formation, using literature reviews and mixed qualitative research methods to deconstruct stoma formation into its component parts and steps; 2) amalgamation of these findings into a 'long list' of potential factors influencing PSH development, and 3) rationalisation of the 'long list' into a pragmatic but systematically determined list of final data items to be collected in the CIPHER study.

Phase 1: identifying the technical risk factors for PSH development

Potential risk factors relating to the technical aspects of stoma formation were defined as those that were a) 'known' (defined as reported in the literature, identified from systematic literature searches) and b) 'unreported but practised' (identified using mixed qualitative methods). Methods to identify each type are described in full below.

a) Systematic literature reviews

To comprehensively identify 'known' technical risk factors, snowballing methods were used to find papers cited by and citing prominent articles relevant to the research question. Snowballing was selected as an efficient alternative to conventional search strategies. Database searches using terms such as 'stoma' and 'parastomal hernia' yielded many abstracts, of which only a small proportion were relevant. In contrast, snowballing provided a manageable volume of results and in-depth review of full-text papers to identify all relevant information relating to the intricacies of stoma formation.

Search strategy

Snowballing began using an index paper, defined as a high-quality paper known to contain information relevant to the study question. The index paper (2) was selected by two senior surgeons both with extensive experience of surgical research. Papers cited by the index paper were then screened (backward snowballing) using broad inclusion criteria (i.e. written in English and providing technical details of small or large bowel stoma formation). Once backward snowballing was complete, all papers citing the index paper were retrieved (forward snowballing) using a citation tracking service. Further index papers (3–9) (selected using expert opinion) were then selected to continue snowballing. This process was continued by two researchers (CM and NB) until no new data were identified (i.e. saturation). The saturation point was tested twice by a second reviewer (NB) who coded additional papers to look for further 'known and reported' technical risk factors for PSH formation.

For peer review only

Data collection and analysis

Searches and analyses of the articles were performed in tandem. Once identified as relevant, papers were uploaded to NVivo 10 (QSR International, Melbourne, Australia). Included papers were read in full to identify all text regarding the technical variations of stoma formation. Once relevant text was identified, a qualitative content analysis was performed and the text was coded into themes, arranged according to the component parts of stoma formation. A proportion of texts were double coded by a second reviewer (LR) to iteratively refine the coding framework. Once saturation was felt to be achieved, a further reviewer (NB) tested the framework by coding further works to identify any additional themes. This was performed twice.

b) Mixed qualitative study

To identify the 'unreported but practised' potential risk factors for PSH, an exploratory study was performed using mixed qualitative methods. This comprised non-participant observation and digital video data capture of stoma formation within the operating theatre, and semi-structured interviews with surgeons and stoma nurses. This work had three objectives, to i) confirm, refine, refute and develop the 'known and reported' factors identified in the literature work, ii) uncover and explore 'unknown but practised' technical variations in stoma formation that may influence PSH development, and iii) triangulate the non-participant observation and semi-structured interview data to clarity and better understand these practices and beliefs. This mixed qualitative study gained NHS Research and Ethics Committee approval from the East Midlands - Nottingham 1 Research Ethics Committee [ref 16/EM/0155] on 1st June 2016. All patients, stoma nurses and surgeons provided written consent to be observed and digitally video recorded in theatre and/or be interviewed. Verbal consent was taken from additional healthcare personnel present in the operating theatre, for observations and digital video recording.

Sampling

Operations including stoma formation were purposefully sampled from two NHS hospital centres. We approached key informants with a role in conducting stoma surgery or providing aftercare. This included colorectal and general surgeons at consultant and registrar level, and stoma nurses. Sampling continued up until the point of saturation, whereby during parallel analysis no new themes were uncovered and additional data were not adding any further explanatory or descriptive benefit.

For peer review only

Data collection

Non-participant observations of stoma formation were undertaken by CM (a medically qualified researcher) and documented as field notes. Observations were supplemented by digital videos of the procedure, filmed by theatre staff or a member of the medical illustration team using portable video recorders in conjunction with above-head theatre cameras (where available). Recording started when stoma formation began and stopped upon completion, when the stoma bag was attached. All recordings were stored on encrypted and secure devices.

Semi-structured interviews were conducted by CM. All interviews were digitally audio-recorded and transcribed verbatim. Interviews with surgeons were held directly after an observation wherever possible, to add explanatory weight to the practices observed and explore inconsistencies between what was observed and what was said. Due to time constraints this was sometimes not possible, and instead interviews were held at a mutually convenient time.

Data analysis

Efforts were made to analyse the data immediately after each interview or observation was completed. This enabled emerging findings to be explored further and scrutinised in the interviews and/or observations that followed. Field notes from observations were aligned with each digital video and watched in full, unedited, by two researchers (CM and NB) independently. The researchers took notes on the videos by documenting the surgeon's movements to create a stepwise account of the surgery, which was imported into NVivo. Semi-structured interviews with surgeons and nurses were transcribed verbatim and also imported into NVivo.

All transcripts were analysed thematically by coding, line by line, to construct themes relating to technical variations in stoma formation. This was an iterative process. CM and LR coded a sample of the transcripts in the early stage of the analysis and met to discuss their coding and agree a consistent framework. LR scrutinised subsequent coded transcripts and CM, LR and NB met regularly to discuss data interpretation and evolution of the coding framework.

Phase 2: Creating a long-list of potential risk factors for PSH

Findings from the literature review and mixed qualitative methods were amalgamated by comparing and contrasting emerging themes. Two researchers, including one senior general surgeon (NB), reviewed each theme and compared the findings of the two data collection processes. The wording of themes and organisation into categories was iteratively refined, combining similar themes and retaining distinction where relevant. Some themes were reclassified as non-technical factors, such as pre-operative bowel preparation. This was a long and iterative process as the coding framework from

For peer review only

both phases interacted and created more complexity within the framework of overarching themes, category themes, and themes (Figure 1). Themes did not always use the same language and were difficult to untangle for the more complex concepts. For example, many surgeons referred to the 'Modified Sugarbaker' technique as 'Sugarbaker' in the interviews, whereas in the literature the two were distinct with different meanings. After removing duplicates, refining the description of the themes and creating exhaustive response categories, a 'long list' of discrete risk factors was compiled, organised sequentially according to the steps of stoma formation.

Phase 3: Agreement of the final list of data items to be collected in the CIPHER cohort study

A consensus meeting was held to achieve agreement about the final list of data items to be collected. Participants were selected using a key-informant approach, where participants were deliberately selected for their expertise as either appointed colorectal surgeons or for their experience in research and publication in the field of parastomal hernia prevention, to represent a broad spread of views. Trial managers were invited to provide views on the practicality of recording information (e.g. measurement of adequate bowel mobilisation). Each risk factor in the 'long list' was introduced by the meeting facilitator and discussed in turn. Attendees were encouraged to consider whether each data item was 'essential', 'desirable' or 'not required' for inclusion in the CIPHER CRFs. Consideration was also given to the wording of each item, to ensure there was no ambiguity to the data items and that the response categories were mutually exclusive. When required, researchers (NB and CM) supplemented discussions with information about the origin of each data item (e.g. from qualitative work or reported in the literature). In the case of items reported in the literature, they also described strength of evidence (e.g. from a systematic review, RCT or case series).

If consensus (i.e. unanimous agreement) was achieved about whether an item was 'essential' or 'not desirable', the facilitator moved onto the next item for discussion. Items where consensus was not met or those considered 'desirable' were carried forward to the next 'round' of discussion, when they were reconsidered in light of how all other items had been prioritised.

Further meetings with the trials design team were held to map the items prioritised and refined through consensus using design principles for CRFs.

Patient and public involvement

Patients and the public were involved in all aspects of the design and conduct of the CIPHER study.

For peer review only

Results

Phase 1: Identifying the risk factors for PSH development

a) Literature review

Data saturation was achieved after undertaking forward and backward snowballing of six index papers (Table 1). A total of 490 references were screened and of these, 130 articles finally included. Within these articles, 138 items relating to the technical aspects of stoma formation, hypothesised to influence PSH prevention, were identified.

For peer review only

Table 1: Approach taken to literature snowballing

Index paper	Description of index paper	References forward snowballing	References backward snowballing	Articles excluded	Articles not located	Duplicates	Articles eligible	Additional articles included in the review (excluding duplicates)
Shabbir, 2012 (2)	Systematic review	34	42	12	1	0	63	40
Aquina, 2014 (3)	Review article	108	13	12	1	15	93	38
Hauters, 2016 (4)	Prospective cohort study	29	15	1	0	19	24	10
Prudhomme, 2016 (5)	Randomised control trial	30	0	0	0	19	11	6
Hotouras, 2013 (6)	Systematic review	115	42	12	6	37	102	26
Hardt, 2013 (7)	Systematic review	52	9	4	3	29	25	6
Additional articles suggested by experts: Brandsma, 2016 & López-Cano, 2016 (9)	Randomised control trials	2	0	0	0	0	0	2

For peer review only

b) Mixed qualitative methods

Six procedures involving stoma formation were observed (Table 2) and thirteen health professionals were interviewed across two centres (Table 3). Interviews lasted between 17:22 and 1:13:95 minutes (mean = 27:45 minutes). The qualitative work identified 150 items relating to technical factors that were thought to influence the development of PSH.

Table 2: Characteristics of the non-participant observation patient sample

Sex	Age	Ethnicity	Type of stoma formed	Surgical approach to stoma formation	Indication for stoma formation	Planned or unplanned surgery	Centre number
Male		White British	End colostomy	Laparoscopic	Bowel management for paraplegia	Planned	1
Male		White British	End colostomy	Laparoscopic	Bowel management for multiple sclerosis	Planned	1
Female		White British	End colostomy	Open	Bowel cancer	Planned	2
Female		White British	End Ileostomy	Laparoscopic	Inflammatory Bowel Disease	Planned	1
Male		White British	End Ileostomy	Converted laparoscopic to open	Bowel cancer	Planned	2
Male		White British	End colostomy	Open	Sigmoid volvulus	Unplanned	2

For peer review only

Table 3: Healthcare professional interviewee characteristics

Sex	Specialty	Grade/role	Length of time in role (years)
Female	Upper gastrointestinal	Consultant	16
Female	Colorectal	Stoma nurse	12
Female	Colorectal	Stoma nurse	3
Male	Colorectal	Consultant	<1
Male	Upper gastrointestinal	Consultant	<1
Male	Upper gastrointestinal	Consultant	1
Male	Colorectal	Consultant	3
Male	Colorectal	Consultant	11
Male	Colorectal	Consultant	3
Male	Colorectal	Registrar	7
Male	Colorectal	Consultant	6
Female	Colorectal	Stoma nurse	5
Male	Colorectal	Consultant	2

Phase 2: Amalgamation of Phase 1 findings into a long-list of data items

Following amalgamation of the themes for the Phase 1 data collection processes and exclusion of duplicates, 180 technical factors were identified (40 were unique to the literature, 63 unique to the qualitative work, 77 appeared in both). This long-list of individual data items was arranged into tables to present the data in a format that was easy to manage for the consensus meeting. Formatting of the long-list into tables resulted in the splitting and condensing of some data items as well as the addition of further response categories such as 'other' where appropriate. The long-list was reviewed by two senior colorectal surgeons from different centres who were not attending the consensus meeting. This was to check for the researchers' understanding of terminology and also to ensure that all factors had adequately been identified. This review resulted in the addition of 12 new data items that were felt to be of interest. A total of 207 data items were taken to the consensus meeting for discussion (a segment of the long-list is provided in Supplementary Table 1).

For peer review only

The data items were arranged into eight overarching category themes: i) surgical approach to stoma formation; ii) trephine formation (skin and subcutaneous tissue, muscle layers, other); iii) reinforcing the stoma trephine with mesh; iv) closure of the lateral space; v) use of the stoma as a specimen extraction site; vi) stoma snugness; vii) closure of other wounds formed during the procedure; viii) creating and securing the stoma lumen. Each overarching category theme is discussed in greater detail below using examples from the qualitative work to demonstrate their reported significance to parastomal hernia prevention.

i) Surgical approach to stoma formation

Findings from both the literature and qualitative work suggested several aspects of the surgical approach to stoma formation may influence the development of PSH. Many variations to operative technique were identified in the literature, with further depth and detail added by the qualitative work. These included: the overall approach to stoma formation (open, laparoscopic, mixed); the section of bowel used to create the stoma; the length of bowel mobilised; whether the stoma site was 'pre-marked'; and the route of the stoma through the abdominal wall (Table 4).

Table 4: Examples from the qualitative data representing the development of the overarching category theme *'Surgical approach to stoma formation'*

Sub theme	Extract
Surgical approach to stoma formation	*Extract 1:"I would favour using a laparoscopic technique if that was technically possible. The reason being that you can actually confirm the anatomy so you can perform an end colostomy, which I think reduces the risk of parastomal formation and the other complications such as prolapse and retraction. You can actually mobilise the colon to bring it up to the abdominal wall."[HCP: BRI0022, Surgeon Lower GI, RDE]*<br>*Extract 2: "There is talk, that maybe sometimes laparoscopic surgery they end up with more hernias, but to me I think it's when they've had a full-blown laparotomy and then they have a stoma form. I think they're probably more at risk because they've got double… They appear to have weaker muscles, don't they?" [HCP: BRI0003, Stoma Nurse, BRI]*
The section of bowel used to create the stoma	*Extract 3: "It does, you can do end stoma rather than a loop. The loop ones I think we tend to have a lot more trouble with prolapse, retraction and herniation because you have to make a bigger cut to bring up the loop of the colon." [HCP: BRI0022, Surgeon, Lower GI, RDE]*<br>*Extract 4: "An end seems less likely; a loop ileostomy seems more likely to get a parastomal hernia than an end ileostomy. I'm trying to think if colostomies seem less likely to get an earlier-on hernia and more likely to get a later-on hernia." [HCP: BRI0002, Stoma Nurse, BRI]*

For peer review only

Length of bowel mobilised	*Extract 5: Interviewer: "What do you think about the amount of bowel mobilised? Do you think that would make a difference to parastomal hernias?"*<br>*Respondent: "Probably. Yes, you're probably right, probably more with stoma prolapse or retraction, rather than parastomal hernias. I suppose if you have a prolapsing stoma, it would widen the defect. I don't know." [HCP: BRI0009, Surgeon, Upper GI, BRI]*
Pre-marked stoma site	*Extract 6: "The stoma site is pre-marked at two sites above and below and left lateral to the umbilicus. Both sites have been tied with a suture." [Observation CM: BRI0014, End colostomy, Laparoscopic, BRI]*<br>*Extract 8: Interviewer: "You also mentioned preoperative marking of the stoma site. Do you think that will make a difference to the parastomal hernia rates?"*<br>*Respondent: "It probably doesn't, it is probably more about having a better site for the patient in terms of a place where they can change it. I suppose sometimes if it is marked in a position that is not ideal for a surgeon it can be more challenging for us. If anything is more challenging you might increase things marginally, but probably not." [HCP: BRI0022, Surgeon, Lower GI, RDE]*
Route of the stoma through the abdominal wall	*Extract 9: "Technical factors associated with parastomal. So, I suppose one thing to address is whether we do this as a trans-peritoneal, or an extra peritoneal approach. So, years ago, in the '60s and '70s, and maybe even more recently, it was quite common for the stomas to be tunnelled, pre-peritoneally, laterally, so essentially what you were doing is you'd have the bowel up laterally against the abdominal wall or the under surface, and then it would come out through the muscles as an extraperitoneal stoma. That may have an impact, I don't know, that's never been subjected to a randomised trial comparing it to the trans-peritoneal approach, where the bowel simply just comes through the abdominal wall without tunnelling it. So I think that's a possible surgical technique factor." [HCP: BRI0004, Surgeon Lower GI, BRI]"*

ii) *Trephine formation:*

The term 'trephine' describes the hole or opening in the abdominal wall for the stoma to pass through. Trephine formation was divided into three subsections: skin and subcutaneous fat; muscle layers; and other.

a) Skin and subcutaneous tissue

All of the variations in this theme were identified in the non-participant observations, with some being common to the literature and and/or the semi-structured interviews. Various techniques were employed by surgeons to form the trephine of the skin, including the instrument used to create the incision as well as size and shape of the incision (Supplementary Table 2). How the trephine was formed through the subcutaneous tissue was also raised as a possible technical factor that could

For peer review only

impact on PSH rates. Variations involving the subcutaneous tissue included whether adipose tissue was excised or divided, and (if excised), whether this was done in the shape of a cone or column.

b) Muscle layers

Both the literature and mixed qualitative methods identified multiple variations relating to trephine formation through the abdominal muscle layers. Many were uniquely found in either the literature or the qualitative work. Overall, both the literature and qualitative work indicated that factors surrounding the muscle trephine may be important for PSH prevention. Sub-themes within this theme included the location of the muscle trephine, methods used to make the incision through the anterior rectus sheath, posterior rectus sheath, how the stoma trephine was created through the muscle fibres and methods to measure and dilate the stoma trephine. Interviews revealed different surgical preferences for where the trephine should be created, with some reporting that trephine formation within the rectus sheath was superior for PSH prevention, whilst others were unsure of the significance of the trephine location. Informants also described diverse practices for how the trephine was formed through the different layers of the abdominal wall. Some described using circular incisions to incise the anterior and posterior sheath. This was thought to prevent PSH through an improved distribution of tensile forces (Supplementary Table 2). Others preferred a cruciate incision to create the trephine with a 'minimal defect'.

c) Trephine formation: Other

This overarching category theme related to intraoperative nerve injury and factors relating to laparoscopic procedures, such as level of gas inflation during the trephine formation and use of the stoma trephine as a port site. The sub-themes specific to laparoscopic procedures were identified solely in the qualitative work, particularly the semi-structured interviews. As shown in the extract below, the level of inflation and use of the stoma site as a port-site were anticipated by some to impact on PSH occurrence:

Extract 1: "I would generally deflate, I think it's important to deflate the abdomen before making a stoma cut…so from what we've talked about so far, what you want is a hole that's closely mirrors the diameter of the bowel so not too much so that you can get a parastomal hernia, not too little so that it… you're gonna get it pinched. So, to create that doing it under tension with the CO2 inside pushing up against the fascia just doesn't make sense to me."

Extract 2: Interviewer: "I've seen people perform laparoscopic surgery through the stoma site…so will use a [Alexis] wound protector and a glove; do you think that will have an affect on parastomal hernia rates?"

For peer review only

Respondent: "Yeah I think exactly the same reason so I don't do it. Nice idea I know people are desperate to keep their wounds down, but if you've got a sub total colectomy for a permanent ileostomy which is with the patient for the rest of their life, I think you're gonna get a hernia and they would probably, I have never actually done it with anyone but they would probably narrow the stoma hole down later and they're using stitches, and yet we've said that repairing a parastomal hernia with stitches has got a 100% recurrence rate" [HCP: BRI0036, Surgeon, Lower GI, BRI]

iii) Reinforcing the stoma trephine with mesh

The use of mesh to reinforce the stoma site at the primary stoma formation was an overarching theme throughout the literature and qualitative work with both sources providing rich data. The use of mesh was a commonly published topic; however, the qualitative work demonstrated uncertainty on its value:

Extract 1: "I put an intraperitoneal mesh to stop the parastomal hernia. That is my anecdotal experience, I've done 10 or 15 of those and I think they are less likely to get hernias, but even if they get hernias, they're less likely to be symptomatic." [HCP: BRI0010, Lower GI, BRI]

Extract 2: "I've seen meshes eroding through the bowel, so I'm reluctant to use it." [HCP: BRI0010, Surgeon, Lower GI, BRI]

There were also variations in practice and views in relation to the type of mesh, its size and shape, location, positioning, the size and shape of trephine through the mesh and how the mesh was secured (Supplementary Table 3).

iv) Closure of the lateral space

Closure of the lateral spaces was referred to in both the literature and the semi -structured interviews as a method of PSH prevention. One surgeon theorised that a method to close the lateral space could influence PSH formation by closing a space where a hernia could form, while others were unsure:

Extract 1: "I think having a permanent one, potentially closing the space, you know, probably in the short term, you know, before a bit of bowel has a chance to sort of get its foot through the door metaphorically, after you're closing that space off so there's nothing that can sneak around there while you're waiting for a bit of fibrosis to form, makes sense to me." [HCP: BRI0032, Surgeon Lower GI, RDE]

Closure of the lateral space was performed through 'fascial fixation', the act of attaching the stoma, either the bowel wall (serosa) or the mesentery (the bowels attachment to the posterior abdominal wall where its' blood supply derives from) to the anterior abdominal wall (10). Fascial fixation was discussed in a portion of the semi-structured interviews, and was occasionally performed by two surgeons within their clinical practice (Extract 1 and 2 below), but not others. Sub-themes within this category included fixation of the mesentery to the abdominal wall and fixation of the stoma to the abdominal wall:

Extract 1: "The other thing maybe is whether or not you choose to [tack] the stoma, or choose to tack the bowel to the fascia. So, in does that somehow reduce your risk of parastomal hernia formation? Again, I don't know… I would suture the serosal surface. That's another thing; I don't do any tacking of the mesentery to anything, it's just literally bowel wall." [HCP: BRI0004, Surgeon Lower GI, BRI]

Extract 2: "I suture the colon to the rectus and to the skin separately…" [HCP: BRI0009, Surgeon, Upper GI, BRI]

v) Use of the stoma as a specimen extraction site

Use of the stoma trephine as an extraction site (where the stoma trephine was used to remove the excised piece of bowel) was an overarching category theme only identified through semi-structured interviews and was observed in the non-participant observation:

Extract 1: "The specimen is handed out. With the specimen removed, I can now see that all that bowel did seem to come out of the stoma hole. Impressive! Won't that have stretched it?" [Observation NB: BRI0033, End Ileostomy, Laparoscopic, RDE]

Some surgeons interviewed felt strongly that this would be a high-risk factor for PSH development due to the stretching and extending of the stoma trephine size to accommodate the delivery of the often diseased bowel (see extracts below). Other sub themes included in this overarching category theme were the type of specimen (colon or small bowel) that was brought through the stoma trephine and adjustments to the trephine size to facilitate delivery of the bowel. These factors were also proposed to influence the likelihood of PSH, as shown below:

Extract 2: *"What I feel strongly about is when you use the stoma site to extract a specimen you do predispose them to parastomal hernias so if you're doing a, predictly (sic) if you're doing a subtotal colectomy which is the way it's tempting to do it, you're taking a diseased colon out through a*

hole which eventually is gonna become an ileostomy site and yes you can narrow it down with sutures afterwards but if we know that parastomal hernia repairs, repair by these sutures have an almost 100% recurrence rate then it's gonna get a parastomal hernia… I think it is important… you're making a hole to get the colon out which is um you know much bigger than the small bowel you're gonna leave there eventually. So technically if you do make the hole too big you're get a parastomal hernia there's no doubt about it." [HCP: BRI0036, Surgeon, Lower GI, BRI]

Extract 3: *"You then have to create a very large hole for the specimen to come out of, and, as we discussed before, the size of the hole… Once you've made it big, making it smaller again is really challenging."* [HCP: BRI0023, Lower GI, RDE]

Extract 4: "The other thing is whenever I, if I ever widen the trephine to deliver a specimen, I then to tend to put a couple of stitches in to try and narrow it again, but I don't think that's as good as not opening it in the first place, if that makes sense." [HCP: BRI0032, Surgeon Lower GI, RDE]

vi) Stoma snugness

When the bowel is being brought through the stoma trephine to form the stoma, it may require further adjustments following an assessment of how 'snug' the bowel is within the stoma trephine- a term used by some interview participants to describe the fit of the stoma in the abdominal wall (Table 5, Extract 1). During the non-participant observations, it was observed that one of the surgeons assessed for *'snugness'* by inserting a finger into the stoma trephine and making a further incision to the abdominal wall (Table 5, Extract 2). This was also discussed in the interviews (Table 5, Extracts 3). A further subtheme within this overarching category theme was mesentery stripping, where one surgeon theorised excessive removal of the stoma's mesentery could cause ischemia and therefore predispose to PSH (Table 5, Extract 4).

For peer review only

Table 5: Examples from the qualitative data representing the development of the overarching category theme *'stoma snugness'*

Sub theme	Extract
Assessment of stoma snugness	*Extract 1: "It is better to pull it through and think, "That is a bit snug." Then making a bit of a nick in the posterior sheath to make it a bit wider or using your Langenbeck's to sometimes just stretch it. That does vary between whether you do that sharp or blunt." [HCP: BRI0022, Surgeon, Lower GI, RDE]*<br><br>*Extract 2: "With the surgeons index finger he checks for snugness of the trephine. On deciding it is too tight he requests a langenbeck retractor and places it along side his finger, asking the SpR to hold it and retract. Using mackindo scissors he makes a further incision in the peritoneum. Consultant re-checks for snugness on either side of the stoma, checking from inside of the abdomen and outside." [Observation CM: BRI0034, End Ileostomy, Converted laparoscopic to open, BRI]*<br><br>*Extract 3: "The traditional has been one finger in, one finger in, and a bit of pulling and tearing and everything else." [HCP: BRI0023, Lower GI, RDE]*
Mesentery stripping	*Extract 4: "What you're trying to avoid is stripping the mesentery off, cos if you strip the mesentery off the bowel it's ischemic and it will go dusky and flat so you might create a bigger whole and then you've got a risk factor for parastomal hernia." [HCP: BRI0036, Surgeon, Lower GI, BRI]*<br><br>*Extract 5: "If the appendices epiploicae are large I'll remove them but I want it under no tension and subcuticular to extramucosal sutures with all the knots buried." [HCP: BRI0015, Surgeon, Lower GI, RDE]*

vii) Closure of other wounds formed during the procedure

Closure of other abdominal wounds formed during the procedure was identified as themes in both the literature and qualitative work. The qualitative work provided rich data on the importance of closing the other abdominal incisions for PSH prevention. For example, BRI00023 felt strongly that restoring the anterior abdominal wall's function through closure of the deep layers using small bite closure with a specific suture size and type was superior for PSH prevention (Table 6, Extract 1). A further sub-theme identified was the order of the wound closure. Some interviewees stated their preference for closing the anterior abdominal wall incisions prior to securing the lumen of the stoma, this practice was also confirmed in the observations (Table 6, Extracts 3 and 4).

For peer review only

Table 6: Examples from the qualitative data representing the development of the overarching category theme *'closure of other wounds formed during the procedure'.*

Sub theme	Extract
Layers of wound closure (Deep layers; Skin layers)	*Extract 1: "How you close the abdominal wall I think is really important, because it then affects how likely the patient is to develop an incisional hernia. If the patient develops an incisional hernia that will impact, because of the mechanics of the abdominal wall on the stoma aperture and then lead to development of parastomal hernias. The two are intimately related. You have to take every possible step to ensure that you have good abdominal wall closure, and restoration of appropriate function, so we tend to use the small bite closure technique using 2-0 PDS delayed absorbable sutures. It's been standard practice now for about two years, particularly for primary surgery." [HCP: BRI0023, Lower GI, RDE]*<br><br>*Extract 2: "Typically I would use a glue to give you a seal. Then you haven't got a dressing extending from the edges of the main wound, that might impinge on where your stoma bag would sit. Also the glue, even if there are leakages, will give you a seal over the centre of the wound." [HCP: BRI0018, Surgeon, Hepatobiliary, RDE]*
Order of wound closure	*Extract 3: "At this point the end of the bowel that I've brought through is typically stapled off, and I will leave it stapled off when it's drawn through the stoma. Then we would finish any further intraabdominal work, close the anterior abdominal wall, close the skin, dress the skin." [HCP: BRI0018, Surgeon, Hepatobillary, RDE]*<br><br>*Extract 4: "4.0 monocryl on curve and PDS sutures close midline umbilical port site. Mostly out of view with the handheld. Monocryl for port smaller lateral port sites. Wet and dry. Glue used (theory to reduce stoma infections). Two babcocks on distal edges of stapled stoma. Out of view. Lotus dissects the stapled line. Fine tooth forceps and 4.0 monocryl on curve secures stoma to skin with slight spout at 3, 12, 9 then 6 o'clock positions." [Observation CM: BR0021, End Colostomy, Laparoscopic, RDE]*

viii) Creating and securing the stoma lumen

How the stoma lumen was created and secured was an overarching category theme identified in both the literature and qualitative work. Subthemes included efforts to reduce faecal spillage and cleaning of the stoma lumen. Further detail was provided, particularly from non-participant observations, which identified multiple methods of suturing the stoma lumen. It was not clear if interview

For peer review only

participants perceived these factors to affected PSH rates, but there were many different reported and observed practices to creating and securing the stoma lumen.

Phase 3: Agreement of the final list of data items to be collected in the case report forms

Consensus methods

A consensus meeting was held with eight professionals, chaired by the CIPHER study's Chief Investigator (Table 7). Decisions were made through discussion and voting. Whilst consensus on the classification of most data items was simple, others proved more difficult. Some items such as 'length of bowel mobilised' were agreed to be important, but discussion raised concerns over the difficulty in obtaining intraoperative measurements as well as the reliability of surgeons' self-reporting. This item was therefore classified as 'undesirable'. For data items where consensus was unclear, the panel took an open vote by raising their hands. While this method resolved some of the conflict, for a small proportion of the data items, disagreement persisted. These data items were classed as 'desirable' and re-discussed at the end of the meeting. For 15 data items, further discussion did not resolve the differing views and it was decided that these data items would remain 'desirable' and would be provisionally retained, with a final decision to be made during design of the CRFs.

Table 7: Characteristics of the consensus meeting participants

**Gender**	**Specialty**	**Grade**	**Trust**
Male	Colorectal	Consultant	Royal Devon and Exeter
Male	Colorectal	Consultant	Queen Elizabeth Hospital
Male	Colorectal	Consultant	Bristol Royal Infirmary
Male	Colorectal	Consultant	Royal Surrey County Hospital
Female	Colorectal	Specialty Trainee (ST7)	Southmead Hospital
Female	Professor of Surgery	Consultant	Bristol Royal Infirmary
Male	Professor of Health Services Research	Non-clinician	Bristol Royal Infirmary
Male	Health services provider	Non-clinician	Bristol Royal Infirmary

For peer review only

Four data items that were suggested during the consensus meeting were also included in the final short list: i) name of the primary procedure, ii) indication for surgery, iii) excision of anterior sheath during trephine formation, and iv) excision of posterior sheath during trephine formation. Additional amendments to the list included wording changes, condensing of items, and creating of additional response categories. A total of 207 data items were discussed. These items were categorised as essential (n=56), desirable (n=15) and undesirable (n=136). The undesirable items were deemed not important to collect in CRFs and therefore excluded.

Constructing the short-list of data items into data fields involved two meetings between the research team, the trials unit staff and the Chief Investigator of the CIPHER study. Due to the number of items deemed 'essential' during the consensus meeting it was decided that the majority of 'desirable' items could not be included in the final CRFs. Each data item was considered individually, and only three of the fifteen desirable items were included in the final CRFs (use of sutures to buttress the end of the trephine anterior sheath; use of sutures to buttress the end of the trephine posterior sheath; damage to epigastric vessel), because they were simple and collecting them would not increase the burden on participating surgeons. This decision was supported by the members of the consensus meeting, who were contacted latterly. Amendments were made to phrasing and formatting of the essential items but the content/meaning of the items remained unaltered. The final number of data items included in the CRFs was 59 (Supplementary Table 4). Combining expert opinion and the trials unit staff knowledge of CRF practicalities was key to ensuring the data items were mapped clearly on to the CRFs, enhancing data collection completeness. These collaborative discussions also enabled any disparity between surgeon and trials unit members expectations and any residual ambiguities between the data items to be resolved.

Adherence to the CRFs and any identified associations between surgical components of stoma formation and the risk of developing parastomal hernia will be reported in the results of the main CIPHER study (Additional file 2).

Discussion

This novel study used a combination of literature reviews, mixed qualitative methods and consensus processes to identify key components of a surgical procedure. This informed development of CRFs for a cohort study investigating risk factors for the development of PSH. Initially, 180 data items were identified, which was rationalised to 59 to be recorded in the CRFs. This application of methods has enabled the development of a systematic process for recording how surgical procedures are conducted in clinical studies, resulting in improvements in transparency of intervention delivery and

For peer review only

subsequent implementation in practice. The use of scientifically informed CRFs in trials will be valuable to examine protocol adherence and associations between surgical components and outcome.

Previous studies investigating risk factors for the development of PSH have tended to focus on technical variations of interest to the investigators and wider surgical community (e.g. prophylactic mesh placement(11), trephine location (12)). In some, a selection of other technical factors were standardised within the study protocol, although no commentary or justification was provided for these. A typology of surgical procedures, developed to identify components of surgical interventions within RCTs, could have been employed to facilitate the process of ascertaining how much standardisation is required for each intervention component (13). However, because this approach was designed for RCTs it does not completely suit non-randomised study designs such as CIPHER. The current study modifies and extends the previously developed methods (13) to incorporate how to develop CRFs to capture the delivery of a surgical intervention within its natural setting, recognising that surgical technique is usually variable.

This is a novel study undertaken to inform the development of CRFs in a detailed and systematic way. Despite this, however, there are potential limitations. First, snowballing search strategies are not considered to be as rigorous as systematic database searches of all available literature. As this study focused on achieving saturation of themes, we deemed it unnecessary to identify all the existing evidence and our approach enabled us to identify relevant studies in a more streamlined way. A further limitation is the low number of in-theatre observations across two sites. Despite this, the data retrieved was very rich and data saturation was achieved. Finally, the methods used to reach consensus may be criticised. The meeting was chaired by the study CI who could influence responses, and voting was not anonymous. To mitigate against this criticism the attendees have been deliberately chosen to represent a spread of views.

This study identified numerous potential technical factors relating to the future development of PSH, highlighting the complexity of surgical interventions and the need for this to be carefully considered in feasibility or pilot work prior to a main study. A further key finding from this study is that unique themes relating to the technical process of stoma formation were identified through both literature and qualitative data collection. Moreover, the broad - and at times conflicting - range of surgical practices/beliefs amongst participating healthcare professionals may represent a lack of evidence and/or awareness of the evidence. Together, these provide strong rationale for undertaking such work prior to a main study, preferably through multiple methods of data collection. The mixed methods approach has enabled the breadth of data items included in the final CRFs to be more comprehensive than if they were based solely on the opinions and interests of the CIPHER study team. Nesting this

For peer review only

work within the early stages of the CIPHER study may have helped to reduce the risk of omitting key variations in stoma formation that may affect PSH development.

This study used multi-modal data collection to enable systematic identification of the components and technical variations of a complex surgical intervention. This informed the rigorous development of CRFs for a future cohort study (CIPHER) aiming to determine risk factors for a key complication of stoma formation surgery (PSH). Through a mixed methods approach, data items were rationalised into a manageable number to include those considered most important. These methods will enable future studies investigating surgical interventions to comprehensively design CRFs to reflect the complexity of the intervention, making it possible to understand exactly how an intervention was delivered within a study so that appropriate conclusions can be drawn.

Contributor statement

NB, LR and JB conceived the idea for the study. CM, LR, JB and NB wrote the protocol. CM undertook the literature and qualitative work supervised by LR, JB and NB. NS, ID, TP, JS, TR, JB, JT, JR, BR, and HB all contributed to the consensus meeting. CM, NB, LR and JB drafted the manuscript and all authors read and approved the final manuscript.

Competing interests

The authors declare that they have no competing interests.

Funding

This work was performed as part of the NIHR funded UK **C**ohort study to **I**nvestigate the prevention of **P**arastomal **HER**nia (CIPHER) Study: Understanding surgery and current practice in stoma formation and developing Patient Reported Outcome Measures for Parastomal Hernia to inform Phase B of CIPHER. Part A was led by Professor Jane Blazeby and supported by the Bristol-based Clinical Trials and Evaluation Unit (CTEU), Royal College of Surgeons surgical trials centre, the MRC ConDuCT-II Hub for Trials Methodology Research, and the NIHR Biomedical Research Centre at University Hospitals Bristol and Weston NHS Foundation Trust and the University of Bristol. The views expressed are those of the author(s) and not necessarily those of the NIHR or the Department of Health and Social Care. Jane Blazeby is an NIHR Senior Investigator and Natalie Blencowe is an MRC Clinician Scientist (MR/S001751/1).

For peer review only

Data sharing statement

The datasets generated and analysed during the current study are not publicly available due to confidentiality reasons.

Ethics approval

This study gained National Health Service (NHS) Research and Ethics Committee approval from the East Midlands - Nottingham 1 Research Ethics Committee [ref 16/EM/0155] on the 1st of June 2016. Ethical and local agreements were in place for both NHS trusts prior to commencing the research.

Acknowledgements

The CIPHER team would like to thank those at the CTEU and the Stoma Nurses from Bristol Royal Infirmary and the Royal Devon and Exeter Hospital for their invaluable contribution to this study.

Figure 1: Example of the coding framework for mesh trephine size

For peer review only

References

1. Avery KN, Barham CP, Berrisford R, Blazeby JM, Blencowe NS, Donovan J, et al. Understanding surgical interventions in RCTs: the need for better methodology. The Lancet. 2013;381(9860):27–8.

2. Shabbir J, Chaudhary BN, Dawson R. A systematic review on the use of prophylactic mesh during primary stoma formation to prevent parastomal hernia formation. Colorectal Dis. 2012;14(8):931–6.

3. Aquina CT, Iannuzzi JC, Probst CP, Kelly KN, Noyes K, Fleming FJ, et al. Parastomal hernia: a growing problem with new solutions. Dig Surg. 2014;31(4–5):366–76.

4. Hauters P, Cardin J-L, Lepere M, Valverde A, Cossa J-P, Auvray S, et al. Long-term assessment of parastomal hernia prevention by intra-peritoneal mesh reinforcement according to the modified Sugarbaker technique. Surg Endosc. 2016;30(12):5372–9.

5. Prudhomme M, Alline M, Chauvat J, Fabbro-Perray P, Ripoche J, Bertrand MM, et al. Primary prevention of peristomial hernias via parietal prostheses: A randomized, multicentric study (GRECCAR 7 trial). Dig Liver Dis. 2016;48(7):812–6.

6. Hotouras A, Murphy J, Thaha M, Chan CL. The persistent challenge of parastomal herniation: a review of the literature and future developments. Colorectal Dis. 2013;15(5):e202–14.

7. Hardt J, Meerpohl JJ, Metzendorf M-I, Kienle P, Post S, Herrle F. Lateral pararectal versus transrectal stoma placement for prevention of parastomal herniation. Cochrane Database Syst Rev. 2013;(11).

8. Brandsma HT, Hansson BME, Aufenacker TJ, Van Geldere D, Van Lammeren FM, Mahabier C, et al. Prophylactic mesh placement to prevent parastomal hernia, early results of a prospective multicentre randomized trial. Hernia. 2016;20(4):535–41.

9. López-Cano M, Serra-Aracils X, Mora L, Sánchez-García JL, Jiménez-Gómez LM, Martí M, et al. Preventing parastomal hernia using a modified sugarbaker technique with composite mesh during laparoscopic abdominoperineal resection. Ann Surg. 2016;264(6):923–8.

10. Fazio VW, Church JM, Wu JS. Atlas of intestinal stomas. Springer Science & Business Media; 2012.

11. Odensten C, Strigård K, Rutegård J, Dahlberg M, Ståhle U, Gunnarsson U, et al. Use of prophylactic mesh when creating a colostomy does not prevent parastomal hernia: a randomized controlled trial—STOMAMESH. Ann Surg. 2019;269(3):427.

12. Hardt J, Seyfried S, Weiß C, Post S, Kienle P, Herrle F. A pilot single-centre randomized trial assessing the safety and efficacy of lateral pararectus abdominis compared with transrectus abdominis muscle stoma placement in patients with temporary loop ileostomies: the PATRASTOM trial. Colorectal Dis. 2016;18(2):O81–90.

13. Blencowe NS, Mills N, Cook JA, Donovan JL, Rogers CA, Whiting P, et al. Standardizing and monitoring the delivery of surgical interventions in randomized clinical trials. Br J Surg. 2016;103(10):1377.

For peer review only - http://bmjopen.bmj.com/site/about/guidelines.xhtml

For peer review only

For peer review only - http://bmjopen.bmj.com/site/about/guidelines.xhtml

For peer review only

For peer review only

Figure 1: Example of the coding framework for mesh trephine size

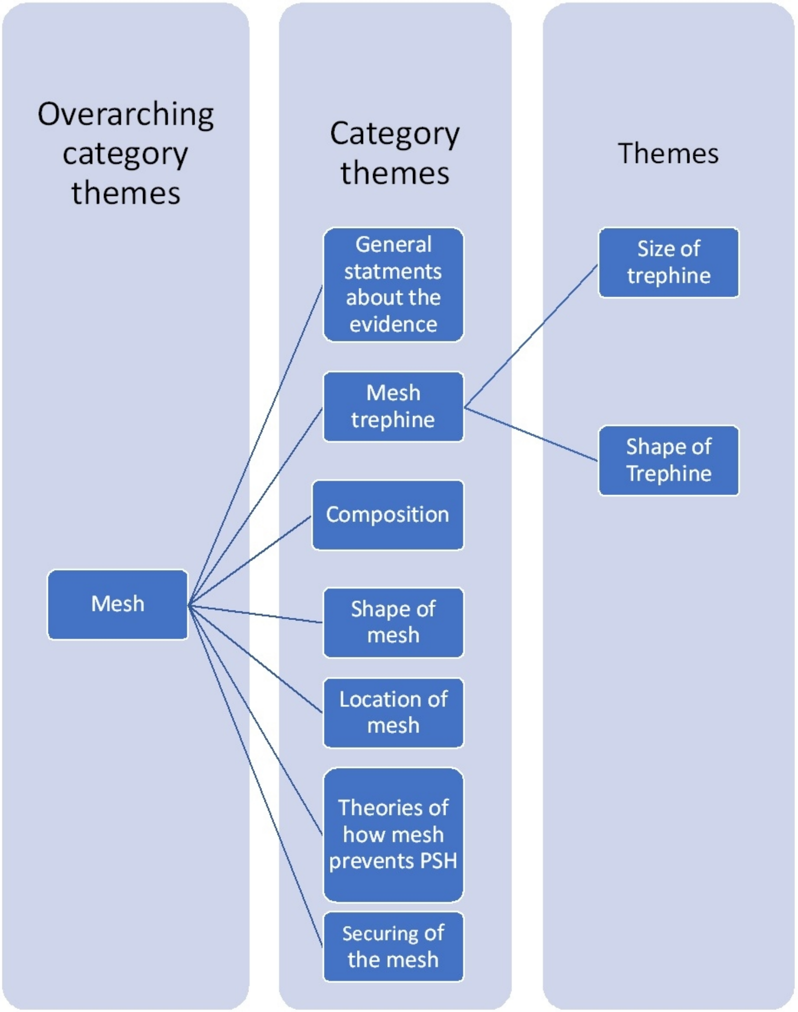

203x222mm (144 x 144 DPI)

Supplementary tables

Table 1: Excerpt from the long-list for the category theme '*Trephine formation: Muscle layers*'.

	**Technical variations**	
**a. Relationship to rectus abdominis**	Within the belly of rectus	
	Outside the belly of rectus	Within rectus sheath
		Within oblique abdominal muscles
	Other	
**b. Relationship to arcuate line**	Above	
	Below	
**c. Relationship to linea semilunaris**	Disrupts	
	Does not disrupt	
	Uncertain	
**d. Anterior sheath**	No incision made	Blunt puncture [trocar, scissors, mechanical device, other]
		Other
	Incision made	Size of incision [widest diameter in cm]
		Shape of incision [linear (horizontal, vertical, cruciate, circular, other]
		Use of sutures to close the margins?
		Measurement of incision size [finger breadths, ruler, by eye, other]
	Adjustments made to incision/puncture	Blunt [retractor, manually, scissors, other]
		Sharp [scalpel, diathermy, other]
**e. Posterior sheath**	No incision made	Layer punctured bluntly [trocar, scissors, mechanical device, other]
		Other
	Incision made	Size of incision [widest diameter in cm]
		Shape of incision [linear (horizontal, vertical), cruciate, circular, other]
		Measurement of incision size [finger breadths, ruler, by eye, other]
	Use of sutures to close the margins	
	Adjustments made to incision/puncture	Blunt [retractor, manually, scissors]
		Sharp [scalpel, diathermy]
**f. Muscle fibres**	Separated	Blunt [trocar, scissors, mechanical device, other]
		Sharp [scalpel, diathermy]
	Not separated	Retracted sideways (e.g. LRAPS technique)
		Other

For peer review only

Table 2: Examples from the qualitative data representing development of the overarching category theme '*Trephine formation: Skin and subcutaneous tissue; Muscle layers; Other*

Skin and subcutaneous tissues	
**Sub theme**	**Extract**
Skin incision at stoma site	*Extract 1: "The surgeon turns the knife 90 degrees and 'shaves through' the skin around the Langenbeck. This makes a circle shaped incision in the skin. They then achieve haemostasis using forceps diathermy." [Observation NB: BRI0021, End Colostomy, Laparoscopic, RDE]*<br>*Extract 2: "Holding the skin edges with some forceps, the surgeon uses diathermy to incise each edge (skin and subcutaneous tissue), thereby turning the cruciate incision into a oval." [Observation NB: BRI0034, End Ileostomy, Converted laparoscopic to open, BRI]*
Subcutaneous adipose	*Extract 3: "I used to take out a long column of fat and then I thought, "Well, fat is so compressible, what's the point?" And so I just separate it and cut, you know, in a straight line down to the rectus with some protractors… Well, I'm trying to take away- take away as little as possible, to try and minimise the risk of parastomal hernias. So I don't know if it works or not, but again, it's anecdotal other than evidence-based." [HCP: BRI0009, Surgeon, Upper GI, BRI]*<br>*Extract 4: Interviewer: "So you think that may have an effect (on PSH rates)."*<br>*Respondent:" Yes…And I think the fatter somebody is the more fat people excise and therefore the bigger the space because the fat retracts around it." [HCP: BRI0015, Surgeon, Lower GI, RDE]*<br>*Extract 5: "I would have a cone of fat with a circle of skin on the top." [HCP: BRI0018, Surgeon, Hepatobiliary, RDE]*<br>*Extract 6: "No subcutaneous tissue is excised." [Observation CM; RDE, End Colostomy, Laparoscopic, RDE]*
Muscle layers	
**Sub theme**	**Extract**
Location of the muscle trephine	*Extract 7: "I think it makes a difference [to PSH rates]. If you put the trephine within the rectus sheath then the rectus sheath is a closed compartment because you've got a posterior and anterior layer and lateral and medial. Therefore any rise in the force or the pressure within that rectus abdominal muscle by the passage of stoma, or material through a stoma, is equalised by the fact it's a closed compartment so you get are resistant force. If you put it outside the rectus abdominus, in the obliques, then by the very nature of which your obliques and transverse salus separate then they're more likely to separate around a stoma. I think there's a difference in whether the patient's got a posterior sheath or whether you've got a peritoneum because if you've only got a peritoneum you're less likely to have a resisted force because it's dissipated by the peritoneum at the back as opposed to posterior sheath."* [HCP: BRI0015, Surgeon, Lower GI, RDE]

For peer review only

	Extract 8: "I honestly don't know. You could imagine it might [make a difference in PSH rates], but I don't know." [HCP: BRI0018, Surgeon, Hepatobillary, RDE]<br><br>*Extract 9: "Patients who have [a stoma] above the arcuate line of Douglas may be less inclined to have parastomal hernias…Disrupting the linea semilunaris I think is a recipe for disaster."* [HCP: BRI0023, Lower GI, RDE]
Approach to creating the trephine through the anterior sheath	*Extract10: "Some people do transverse. Some do longitudinally. Some make cruciate incisions. I've seen people do incisions say either longitudinally or horizontally, and then buttress the ends with suture material, to try and prevent propagation." [HCP: BRI0023, Lower GI, RDE]*<br><br>*Extract 11: 'By virtue of doing a cruciate incision you are splitting, because you've got a linear incision, it might widen. If we make a circular incision does that mean that the tensal strength or the forces within the abdominal wall, are they going to distribute." [HCP: BRI0004, Surgeon Lower GI, BRI]*<br><br>*Extract 12 "The cruciate incision allows the bowel to come through with the minimum defect. So that's why I've avoided doing the circular incision in the rectus sheath." [HCP: BRI0009, Surgeon, Upper GI, BRI]*
Approach to creating the trephine through the posterior sheath/peritoneum	*Extract 13: ""In terms of technique, I think the size of the cruciate incision, both on the anterior and the posterior layers of the rectus sheath, matter. I think that that's probably, I think, what matters most." [HCP: BRI0001, Surgeon, Upper GI, BRI]*
Approach to creating the muscle trephine	*Extract 14: "Respondent: If you de-vascularise any muscle fibres and it becomes infected then infection leads to hernia formation there is no doubt about that.*<br><br>*Interviewer: So splitting of the fibres improves (PSH rates) that rather than incising them?*<br><br>*Respondent: I think it does, I can't show you evidence to say that. Logically to me if you are splitting the fibres and not disrupting my cutting through the fibres and all the blood supply to the muscle and the surrounding area." [HCP: BRI0022, Surgeon, Lower GI, RDE]*
Dilatation of the stoma	*Extract 15: "Further fibres of muscles/fascial tissue are cut with diathermy" [Observation NB: BRI0021, End Colostomy, Laparoscopic, RDE]*
How the measurements of the stoma trephine are made	*Extract 16: "Well, it's very subjective. Some people would say two fingers, but my two fingers are bigger than your two fingers." [HCP: BRI0010, Surgeon, Lower GI, BRI]*
Other	
**Sub theme**	**Extract**

For peer review only

Intraoperative nerve damage	*Extract 17: "I think if you start making incisions I think you're more at risk at damaging muscles- Not muscles, the nerves that run into the abdominal wall." [HCP: BRI0004, Surgeon Lower GI, BRI]*

For peer review only

Table 3: Examples from the qualitative data representing the development of the overarching category theme '*reinforcing the stoma trephine with mesh*'

Sub theme	Extract
Mesh type	*Extract 6: "See, the fact that all these different meshes are being used for parastomal hernias must mean that no one of them is actually working. The more ways there are of doing something, the more sure you are that nobody knows what the right way is." [HCP: BRI0009, Upper GI, BRI]*<br><br>*Extract 7: "The corners are trimmed off the mesh, making an oval shape. It looks like it's made of polypropylene."* [Observation NB: BRI0021, End Colostomy, Laparoscopic, RDE]<br><br>*Extract 8: Interviewer: "Why do you think Permacol's [biologic mesh] beneficial?"*<br><br>*Respondent: "Compared to [synthetic] mesh? Just because it resists infection better."* [HCP: BRI0032, Lower GI, BRI]<br><br>*Extract 9: "I would use in most patients a piece of polypropylene mesh in the retrorectus position…With the synthetic meshes the rate of tissue ingrowth is so rapid, and scar tissue formation is so quick, that you will find that you will have a fairly solid ring of tissue within four to six weeks, so that you get the protective effect relatively quickly. It's why most of the RCTs would demonstrate nowadays a protective effect with prophylactic mesh."* [HCP: BRI0023, Lower GI, RDE]
Mesh size	*Extract 10: "The consultant measures (using a ruler) the width and length of the retrorectus space and draws a rectangle in permanent marker of the edge of the mesh. The SpR holds the mesh while the consultant cuts using scissors."* [Observation CM: BRI0030, End Colostomy, Open, BRI]<br><br>*Extract 12: "If we do that it involves developing a plane between the muscle layer and the posterior sheath and placing a very small piece of mesh 4cm by 4cm with polypropylene which you pull the stoma through."* [HCP: BRI0022, Lower GI, RDE]
Mesh shape	*Extract 13: "The corners are trimmed off the mesh, making an oval shape. It looks like it's made of polypropylene…The surgeon folds the mesh into quarters, then cuts off the corner, which makes a circular shaped hole in the centre.* [Observation NB: BRI0021, End Colostomy, Laparoscopic, RDE]
Location of mesh	*Extract 14: "The consultant then places the mesh with the retrorectus space."* [Observation CM: BRI0030, End Colostomy, Open, BRI]<br><br>*Extract 15: "Whether that is done intra-peritoneal, whether that's done in a retroperitoneal space, or in an onlay space… So, what I'm saying to you is there's about ten different ways of doing something, which to anyone says that none of them are perfect, and that's never really been subjected to a trial of sufficient power, strength for us to know what the most effective technique is."* [HCP: BRI0004, Lower GI, BRI]<br><br>*Extract 16: "I think the evidence when they did all those prophylactic meshes, whether you put it intraperitoneal, retromuscular or anterior into the muscles, it*

For peer review only

	didn't show any difference in the outcomes. I think it's just strengthening the muscle layer, but probably a large trial will tell us whether there's any difference. In an ideal world, I think you should probably put it either intraperitoneal or retromuscular. I think putting it anterior to the rectus sheath is probably pointless, you might as well not put it, I don't think it adds anything to it because the hernias start from the inside…Yes, it will just lift the mesh; it's not going to make any difference. It either has to be between the rectus and the posterior sheath or intraperitoneal where it might have a use." [HCP: BRI0010, Lower GI, BRI]
How the space is created and method of positioning the mesh	*Extract 17: "Single- index finger feels and sweeps around breath of trephine to create a space in the retrorectus space."* [Observation CM: BRI0021, End Colostomy, Laparoscopic, RDE]<br><br>*Extract 18 "The surgeon uses their finger to create a space, and further fibres of muscles/fascial tissue is cut with diathermy…The surgeon uses a clip to guide the mesh into the pocket that they have created for it. They use their fingers to smooth the mesh out and get it into position."* [Observation NB: BRI0021, End Colostomy, Laparoscopic, RDE]
Mesh trephine	*Extract 19: "But I think, if you're putting a keyhole into any mesh, in mesh prophylaxis, or even as a repair, it's just going to shrink away, that circle is just going to get larger…*<br><br>*Interviewer: "So it contracts… Do you think that's a reason why the Sugarbaker technique would be superior to-"*<br><br>*Respondent: "Yes, so that's what I think, why Sugarbaker would be superior. That said, I don't think it's 100% effective. That's what I would use as a method of mesh prophylaxis and permanent stoma."* [HCP: BRI004, Lower GI, BRI]<br><br>*Extract 20: "If you've got a circular incision maybe you've got a bit more strength around the radius of it, rather than splitting it."* [HCP: BRI0032, Surgeon Lower GI, RDE]
Method of securing the mesh	*Extract 21: 'When I do it laparoscopically I just use the tacks. If I'm doing open surgery I use stitches."* [HCP: BRI0022, Lower GI, RDE]<br><br>*Extract 22: I will use transfascial sutures to place the mesh as well, and that acts partially as a fixation. I will do that and some stapling device as well, and the Sugarbaker is associated with its own unique stapling pattern, because of the way in which it overlies the bowel."* [HCP: BRI023, Lower GI, RDE]

For peer review only

Table 4: Short-list of data items used for development of the CIPHER Study case report forms

**1. Surgical approach to stoma formation**		
**a. Context of stoma formation**	Indication for surgery *(see separate sheet)*	
	Name of procedure *(see separate sheet)*	
**b. Intended type of access used**	Minimally invasive [SILS; Laparoscopic; Robotic]	
	Open	
	Trephine	
**c. Intended type of procedure converted to open**	Conversion from minimally invasive to open [SIL converted, Laparoscopic converted; Robotic converted] Y/N	
**d. Type of stoma formed**	Envisaged longevity of stoma [permanent, uncertain]	
	End	
	Loop	With or without rod
	Loop end (Abcarian)	
	Double barrelled	
	Other	
**e. Section of bowel used to form functioning end of stoma**	Jejunum	
	Ileum	
	Ascending colon	
	Transverse colon	
	Descending colon	
	Sigmoid colon	
**f. Stoma site**	Stoma site pre-marked Y/N [preserved with suture, pen or not preserved]	Stoma site marked by [stoma nurse, surgeon, non-specialist nurse, other]
		Stoma formed/ not formed at pre-marked site (Y/N)
**g. Route of stoma**	Trans-peritoneal	
	Extra-peritoneal *(cannot be trephine in section b)*	
**2. Trephine formation**		
**a. Subcutaneous tissue**	Subcutaneous tissue excised (Y/N)	
**b. Relationship of the muscle layer incision to the rectus abdominis**	Outside of the rectus sheath (Within oblique abdominal muscles)	
	Within the rectus sheath	Through the belly of the rectus abdominis
		Lateral to the belly of the rectus abdominis* (e.g. LRAPS technique)
**c. Anterior sheath *(ONLY if within the rectus sheath*)***	Was a laparoscopic trocar used to puncture the anterior sheath (Y/N) *(only for minimally invasive Laparoscopic + robotic OR converted Laparoscopic + robotic procedure) (NOT SILS or SILS converted)*	
	Size of incision [widest diameter in mm]	
	Shape of incision [linear (horizontal, vertical), cruciate, circular, other]	
	Was any of the anterior sheath removed? (Y/N)	
	Adjustments made to the size of the incision (Y/N)	Sutures used to buttress end of incision (Y/N)

**d. Posterior sheath *(ONLY within the rectus sheath*)***	Was a laparoscopic trocar used to puncture the anterior sheath (Y/N) *(only for minimally invasive Laparoscopic + robotic OR converted Laparoscopic + robotic procedure) (NOT SILS or SILS converted)*		
	Size of incision [widest diameter in mm]		
	Shape of incision [linear (horizontal, vertical), cruciate**,** circular**,** other]		
	Was any of the posterior sheath removed? (Y/N)		
	Adjustments made to the size of the incision (Y/N)		Sutures used to buttress end of incision (Y/N)
**e. Muscle fibres**	Separated with blunt dissection (Y/N) (*only possible if within rectus belly/oblique muscles)*		
**f. Intra-operative vessel damage**	Epigastric vessel (Y/N)		
**g. Laparoscopic procedures (*only for minimally invasive Laparoscopic + robotic OR converted Laparoscopic + robotic procedure) (NOT SILS)***	Location of trephine in relation to port site [Trephine created at port site/ Trephine created in a location other than port site]	Trephine created at port site	Trephine created at the port site at the beginning of procedure [then subsequently used as port site]
			Trephine created at end of procedure [conversion of port site to trephine]
**3. Reinforcing the stoma trephine with mesh**			
**a. Mesh type**	Mesh used (Y/N)		Product code, fix label, provide manufacture name
**b. Mesh size**	Mesh cut or adjusted (Y/N)		Size of mesh inserted if changed from original (Y/N) [height and width measured in cm OR if circular/oval diameter in cm]
			Shape of mesh if changed from original (Y/N) [3D/funnel; Circular/oval; Square/rectangular]
**c. Location of mesh placement**	Please indicate using diagram [Diagram provided with the following drop-down categories: Sublay/pre-peritoneal/retro-rectus; Underlay/intra-peritoneal; Onlay; Inlay] (Retrorectus; Intra-peritoneal; Onlay but keep all descriptions on the diagram)		
**d. Route used to position mesh**	Through the main operative incision (e.g. in an open procedure or an extended port site*) (any type of surgery) (can be all three: onlay, intraperitoneal or retorectus)*		
	Through the stoma trephine *(any type of surgery) (can be all three: onlay, intraperitoneal or retorectus)*		
	Via a port (*Any minimally invasive or converted procedure) (can only be intraperitoneal placement of mesh)*		
	Keyhole Y/N		What shape was the keyhole [cruciate; circular/oval; Slit]
			What was the size was the keyhole *(Maximum diameter)*
**e. Securing the mesh**	Mesh secured to abdominal wall *(including sheath, muscle, peritoneum)* (Y/N)		
	Mesh secured to stoma serosa (Y/N)		
**4. Use of the stoma as a specimen extraction site**			

For peer review only

**a. Stoma trephine used as a specimen extraction site**	Stoma trephine used as an extraction site (Y/N)
**5. Closure of other wounds formed during the procedure**	
**a. Closure of deep layers of the abdominal wall**	Main abdominal incision (e.g. in an open procedure, extended port site or specimen extraction site) [Small bite closure; Large bite closure; NA])
	Biggest port site (e.g. 10, 11 or 12mm) *(Minimally invasive laparoscopic + robotic OR converted laparoscopic + robotic procedures only)* [Closure of deep layer Y/N/NA]
**6. Spouting the stoma lumen**	
**a. Suturing of bowel to skin**	Has the stoma been spouted? (Y/N)

For peer review only

CIPHER STUDY

The CIPHER Study

SURGICAL TECHNIQUE

TH1

Patient Initials: [ ][ ][ ]

CIPHER Study ID: [ ][ ][ ]-[ ][ ][ ][ ]

SURGEON DETAILS

Most senior surgeon scrubbed in at time of stoma formation:

Name: ______________________________ GMC number: ________________

PATIENT DETAILS

Operation date: __ __ /__ __ /__ __ __ __ (d d m m y y y y)

Operation start time (defined as knife to skin: time): __ __ : __ __

Operation end time (defined as time of final skin suture in wound): __ __ : __ __

Month of birth: __ __ Year of birth: __ __ __ __

Patient's Sex: *Male* [ ] *Female* [ ]

SURGICAL APPROACH TO STOMA FORMATION

Indication for surgery (***please select one option***):

- Tumour – benign [ ]
- Tumour – malignant [ ]
- Diverticular Disease [ ]
- Functional Intestinal Disorder [ ]
- Inflammatory Bowel Disease (IBD) [ ]
- IBD – Crohn's [ ]
- IBD – Ulcerative Colitis [ ]
- Other [ ]

If other, specify: ______________________________

ASA Classification:

- I [ ]
- II [ ]
- III [ ]
- IV [ ]
- V [ ]

For peer review only

CIPHER STUDY

The CIPHER Study

TH1

SURGICAL TECHNIQUE

Patient Initials: ☐☐☐

CIPHER Study ID: ☐☐☐-☐☐☐☐

Name of procedures (tick YES or NO as appropriate for each):

	Yes	*No*
Small bowel resection	☐	☐
Colectomy: left (including sigmoid colectomy and anterior resection)	☐	☐
Colectomy: right (including ileocaecal resection)	☐	☐
Colectomy: subtotal or panproctocolectomy	☐	☐
Hartmann's procedure	☐	☐
Colorectal resection – other	☐	☐
Reduction of volvulus	☐	☐
Stricturoplasty	☐	☐
Drainage of abscess/collection	☐	☐
Debridement	☐	☐
Abdominoperineal excision	☐	☐
Posterior pelvic exenteration	☐	☐
Repair or revision of anastomosis	☐	☐
Repair of intestinal fistula	☐	☐
Resection of other intra-abdominal tumour(s)	☐	☐
Stoma formation	☐	☐
Other	☐	☐

If other, specify: ______________________________

If **YES** to ***abdominoperineal excision*** or ***posterior pelvic exenteration***:

Vertical rectus abdominis myocutaneous (VRAM) flap *Yes* ☐ *No* ☐

Intended type of access used (***please select one***):

- SLS ☐
- Laparoscopic ☐
- Robotic ☐
- Open ☐
- Trephine ☐

Intended type of procedure converted to open: *Yes* ☐ *No* ☐
***(Do not answer if intended type of access* was open)**

For peer review only

For peer review only - http://bmjopen.bmj.com/site/about/guidelines.xhtml

The CIPHER Study

TH1

SURGICAL TECHNIQUE

Patient Initials: ☐☐☐

CIPHER Study ID: ☐☐☐-☐☐☐☐

Envisaged longevity of stoma: Permanent ☐ Uncertain ☐

Type of stoma formed (***please select one***):

- End ☐
- Loop ☐
- Loop end ☐
- Double barrelled ☐
- Other ☐

If **loop**, with or without rod: With ☐ Without ☐

Section of bowel used to form functioning end of stoma (***please select one***):

- Jejunum ☐
- Ileum ☐
- Ascending colon ☐
- Transverse colon ☐
- Descending colon ☐
- Sigmoid colon ☐

Stoma site pre-marked (***please select one***):

- Not preserved ☐
- Preserved with suture ☐
- Pre-marked with pen ☐

If ***preserved with suture*** or ***pre-marked with pen***,

Stoma site marked by (***please select one***):

- Stoma nurse ☐
- Surgeon ☐
- Non-specialist nurse ☐
- Other ☐

If other, specify: _______________

	Yes	*No*
Stoma formed at pre-marked site:	☐	☐

Route of stoma: Trans-peritoneal ☐ Extra-peritoneal ☐

For peer review only

For peer review only - http://bmjopen.bmj.com/site/about/guidelines.xhtml

The CIPHER Study

TH1

SURGICAL TECHNIQUE

Patient Initials: ☐☐☐

CIPHER Study ID: ☐☐☐-☐☐☐☐

TREPHINE FORMATION

Subcutaneous tissue excised: *Yes* ☐ *No* ☐

Relationship of the muscle layer incision to the rectus abdominis (***please select one***):

- Outside of the rectus sheath (within oblique abdominal muscles) ☐ → **Continue on next page**
- Within the rectus sheath - though the belly of the rectus abdominis ☐
- Within the rectus sheath - lateral to the belly of the rectus abdominis ☐

Complete the box below

Anterior sheath: was a laparoscopic trocar used to puncture the anterior sheath *Yes* ☐ *No* ☐
(Only answer for laparoscopic or robotic procedures)

Anterior sheath: size of incision [widest diameter in mm] ___________

Anterior sheath: Shape of incision (***please select one***)

- Linear - horizontal — ☐
- Linear - vertical | ☐
- Cruciate + ☐
- Circular ○ ☐
- Other ? ☐

Anterior sheath: was any of the anterior sheath removed *Yes* ☐ *No* ☐

Anterior sheath: adjustments made to the size of the incision *Yes* ☐ *No* ☐

Posterior sheath: was a laparoscopic trocar used to puncture the posterior sheath *Yes* ☐ *No* ☐
(Only answer for laparoscopic or robotic procedures)

Posterior sheath: size of incision [widest diameter in mm] ___________

Posterior sheath: Shape of incision (***please select one***)

- Linear - horizontal — ☐
- Linear - vertical | ☐
- Cruciate + ☐
- Circular ○ ☐
- Other ? ☐

Posterior sheath: was any of the posterior sheath removed *Yes* ☐ *No* ☐

Posterior sheath: adjustments made to the size of the incision *Yes* ☐ *No* ☐

For peer review only

For peer review only - http://bmjopen.bmj.com/site/about/guidelines.xhtml

The CIPHER Study

SURGICAL TECHNIQUE

TH1

Patient Initials: ☐☐☐

CIPHER Study ID: ☐☐☐-☐☐☐☐

Muscle fibres separated with blunt dissection *Yes* ☐ *No* ☐

Intra-operative vessel damage - epigastric vessel *Yes* ☐ *No* ☐

Location of trephine in relation to port site (***please select one***):
(Only answer for laparoscopic or robotic procedures, **including procedures converted to open)**

Trephine created at the port site as the beginning of procedure (then subsequently used as port site) ☐

Trephine created at end of procedure (conversion of port site to trephine) ☐

Trephine created in a location other than port site ☐

Were sutures used to buttress the incision (***please select one***):
(Only answer if relationship of the muscle layer incision to the rectus abdominis is "within rectus sheath")

Anterior sheath only ☐

Posterior sheath only ☐

Both anterior and posterior sheath ☐

Anterior and posterior sheath sutured together ☐

No ☐

For peer review only

For peer review only - http://bmjopen.bmj.com/site/about/guidelines.xhtml

The CIPHER Study

SURGICAL TECHNIQUE

TH1

Patient Initials: ☐☐☐

CIPHER Study ID: ☐☐☐-☐☐☐☐

REINFORCING THE STOMA TREPHINE WITH MESH

Was mesh used to reinforce the stoma trephine *Yes* ☐ *No* ☐ → If **NO**, continue on next page

If **YES**, complete the box below

Mesh product code (attach product sticker) ***Attach product sticker here***

Mesh cut or adjusted *Yes* ☐ *No* ☐

If **YES**,

Craniocaudal length of mesh inserted if changed from original [in cm] ______________

Medio-lateral length of mesh inserted if changed from original [in cm] ______________

Diameter of mesh inserted if changed from original [in cm] ______________

Shape of mesh inserted if changes from original (***please select one***):

- 3D/funnel ☐
- Circular/Oval ☐
- Square/rectangular ☐
- No change in shape ☐

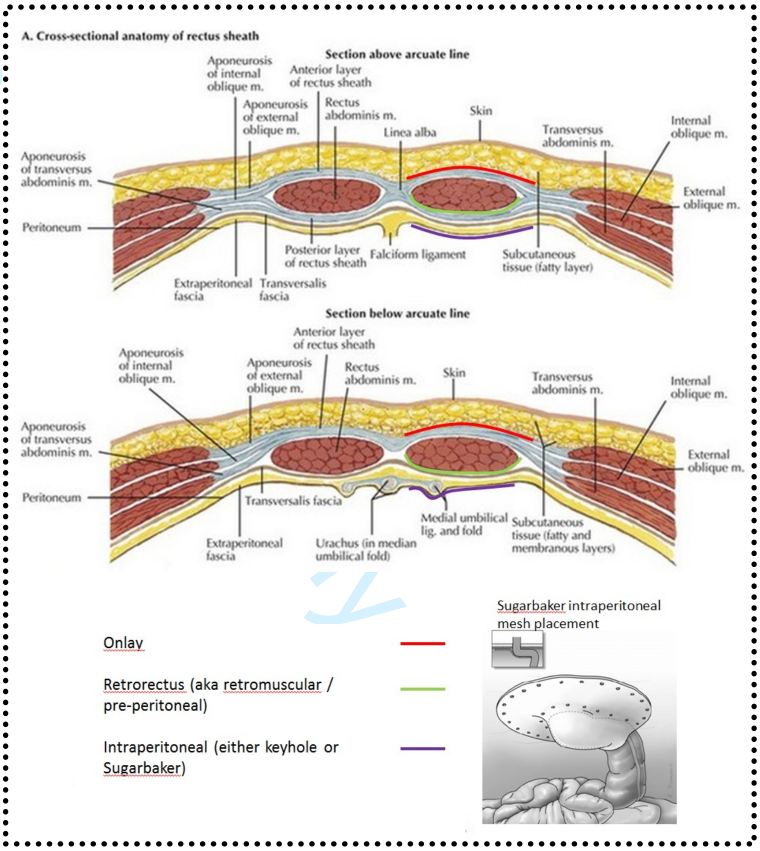

Location of mesh replacement (***please select one***):

- Sublay/pre-peritoneal/retro-rectus ☐
- Underlay/intra-peritoneal ☐
- Onlay ☐

Route used to position mesh (***please select one***):

- Through the main operative incision ☐
- Through the stoma trephine ☐
- Via a port ☐

What shape was the hole in the mesh?

Cruciate ☐ Circular/oval ☐ Slit ☐ None (Sugarbaker) ☐

What size was the hole in the mesh [in mm] __________

Mesh secured to abdominal wall (including sheath, muscle, peritoneum) *Yes* ☐ *No* ☐

Mesh secured to stoma serosa *Yes* ☐ *No* ☐

For peer review only - http://bmjopen.bmj.com/site/about/guidelines.xhtml

The CIPHER Study

SURGICAL TECHNIQUE

TH1

Patient Initials: ☐☐☐

CIPHER Study ID: ☐☐☐-☐☐☐☐

USE OF THE STOMA TREPHINE AS A SPECIMEN EXTRACTION SITE

Stoma trephine used as an extraction site *Yes* ☐ *No* ☐

CLOSURE OF OTHER WOUNDS FORMED DURING THE PROCEDURE

Main abdominal incision (***please select one***):

Small bite closure ☐

Large bite closure ☐

N/A ☐

Biggest port size [in mm] ____________

(Only answer for laparoscopic or robotic procedures, including procedures converted to open)

Closure of deep layer *Yes* ☐ *No* ☐

(Only answer for laparoscopic or robotic procedures, including proce- dures con- verted to open)

SPOUTING THE STOMA LUMEN

Has the stoma been spouted *Yes* ☐ *No* ☐

COMMENTS

____

____

____

____

Who has collated this data?

GMC/NMC number: ______________________________

Date completed: __ __ / __ __ /__ __ __ __

For peer review only

BMJ Open

What should be included in case report forms? Development and application of novel methods to inform surgical study design: a mixed methods case study in parastomal hernia prevention

Journal:	*BMJ Open*
Manuscript ID	bmjopen-2022-061300.R1
Article Type:	Original research
Date Submitted by the Author:	30-Aug-2022
Complete List of Authors:	Murkin, Charlotte; Bristol Medical School, Population Health Sciences<br>Rooshenas, Leila; University of Bristol, School of Social and Community Medicine<br>Smart , Neil ; Royal Devon & Exeter NHS Foundation Trust<br>Daniels, IR; Royal Devon & Exeter Hospital<br>Pinkney, Tom; University of Birmingham School of Clinical and Experimental Medicine, Surgery<br>Shabbir, J; University Hospitals Bristol NHS Foundation Trust<br>Rockall, Timothy; The Royal Surrey County Hospital, Oesophago-gastric Surgery<br>Bennett, J; Gloucestershire Hospitals NHS Foundation Trust, Department of Colorectal Surgery<br>Torkington, Jared; University Hospital of Wales, Department of Colorectal Surgery<br>Randall, J; University Hospitals Bristol NHS Foundation Trust<br>Brandsma, HT; Canisius Wilhelmina Hospital, Department of Surgery<br>Reeves, Barnaby; University of Bristol, Bristol Heart Institute<br>Blazeby, Jane; University of Bristol, Department of Social Medicine<br>Blencowe, Natalie; Bristol Medical School, Population Health Sciences
**Primary Subject Heading**:	Surgery
Secondary Subject Heading:	Qualitative research
Keywords:	QUALITATIVE RESEARCH, SURGERY, EPIDEMIOLOGY, Colorectal surgery < SURGERY

SCHOLARONE™ Manuscripts

I, the Submitting Author has the right to grant and does grant on behalf of all authors of the Work (as defined in the below author licence), an exclusive licence and/or a non-exclusive licence for contributions from authors who are: i) UK Crown employees; ii) where BMJ has agreed a CC-BY licence shall apply, and/or iii) in accordance with the terms applicable for US Federal Government officers or employees acting as part of their official duties; on a worldwide, perpetual, irrevocable, royalty-free basis to BMJ Publishing Group Ltd ("BMJ") its licensees and where the relevant Journal is co-owned by BMJ to the co-owners of the Journal, to publish the Work in this journal and any other BMJ products and to exploit all rights, as set out in our licence.

The Submitting Author accepts and understands that any supply made under these terms is made by BMJ to the Submitting Author unless you are acting as an employee on behalf of your employer or a postgraduate student of an affiliated institution which is paying any applicable article publishing charge ("APC") for Open Access articles. Where the Submitting Author wishes to make the Work available on an Open Access basis (and intends to pay the relevant APC), the terms of reuse of such Open Access shall be governed by a Creative Commons licence – details of these licences and which Creative Commons licence will apply to this Work are set out in our licence referred to above.

Other than as permitted in any relevant BMJ Author's Self Archiving Policies, I confirm this Work has not been accepted for publication elsewhere, is not being considered for publication elsewhere and does not duplicate material already published. I confirm all authors consent to publication of this Work and authorise the granting of this licence.

For peer review only

What should be included in case report forms? Development and application of novel methods to inform surgical study design: a mixed methods case study in parastomal hernia prevention

C Murkin[1], L Rooshenas[1], NJ Smart[2], IR Daniels[2], T Pinkney[3], J Shabbir[4], T Rockall[5], J Bennett[6], J Torkington[7], J Randall[4], HT Brandsma[8], B Reeves[9], JM Blazeby[1], NS Blencowe[1]

On behalf of the CIPHER Study[9]

1. Bristol NIHR Biomedical Research Centre and Centre for Surgical Research, Population Health Sciences, Bristol Medical School, Bristol, UK
2. Exeter Surgical Health Services Research Unit (HeSRU), Royal Devon & Exeter Hospital, Exeter, Devon, UK
3. Academic Department of Surgery, Queen Elizabeth Hospital, University of Birmingham, UK
4. Division of Surgery, University Hospitals Bristol NHS Foundation Trust, Bristol, UK
5. Royal Surrey County Hospital NHS Foundation Trust, Guildford, UK
6. Department of Colorectal Surgery, Gloucestershire Hospitals NHS Foundation Trust, UK
7. Department of Colorectal Surgery, University Hospital of Wales Healthcare NHS Trust, Cardiff, UK
8. Department of Surgery, Canisius Wilhelmina Hospital, Nijmegen, The Netherlands
9. Clinical Trials and Evaluation Unit, Bristol Trials Centre, Bristol Medical School, University of Bristol, Bristol, UK

Acknowledgements

The CIPHER team would like to thank those at the CTEU and the Stoma Nurses from Bristol Royal Infirmary and the Royal Devon and Exeter Hospital for their invaluable contribution to this study.

Corresponding author

NS Blencowe

natalie.blencowe@bristol.ac.uk

Centre for Surgical Research

Population Health Sciences

Bristol Medical School

University of Bristol

For peer review only - http://bmjopen.bmj.com/site/about/guidelines.xhtml

Keywords

Case report forms, cohort study, mixed methods research, qualitative research, literature reviews

Abstract

Objectives: To describe the development and application of methods to optimise the design of case report forms (CRFs) for clinical studies evaluating surgical procedures, illustrated with an example of abdominal stoma formation.

Design: i) Literature reviews, to identify reported variations in surgical components of stoma formation, were supplemented by ii) intra-operative qualitative research (observations, videos and interviews), to identify unreported variations used in practice to generate iii) a 'long list' of items, which were rationalised using iv) consensus methods, providing a pragmatic list of CRF items to be captured in the CIPHER study (Cohort study to Investigate the Prevention of parastomal HERnias).

Setting: Two secondary care surgical centres in England.

Participants: Patients undergoing stoma formation, surgeons undertaking stoma formation, and stoma nurses.

Outcome measures: Successful identification of key CRF items to be captured in the CIPHER study.

Results: 59 data items relating to stoma formation were identified and categorised within six themes: i) surgical approach to stoma formation; ii) trephine formation; iii) reinforcing the stoma trephine with mesh; iv) use of the stoma as a specimen extraction site; v) closure of other wounds during the procedure; and vi) spouting the stoma.

Conclusions: This study used multi-modal data collection to understand and capture the technical variations in stoma formation and design bespoke CRFs for a multicentre cohort study. The CIPHER study will use the CRFs to examine associations between the technical variations in stoma formation and risks of developing a parastomal hernia.

Clinical trial registration

Main CIPHER study ISRCTN17573805.

For peer review only

Article Summary

Strengths and limitations of this study

- This study used a scientific and systematic process to inform the design of case report forms for a main study

- Supplementing literature reviews with qualitative methods helped to highlight important surgical technique variations that may otherwise not have been identified
- A potential limitation is that literature searches were undertaken up to 2016 and therefore further important studies (generating more themes) may have been missed
- Due to the in-depth and multi-modal data collection, this study was time consuming so future studies are needed to streamline the process

For peer review only - http://bmjopen.bmj.com/site/about/guidelines.xhtml

Introduction

A major challenge facing surgical research is that surgical procedures are considered to be complex healthcare interventions, comprising multiple components that can act independently or interdependently to influence outcomes [1]. This complexity means that even surgical procedures labelled with the same name are frequently undertaken in different ways. Such variation has implications for the design and conduct of studies evaluating surgical interventions. It can introduce bias into randomised controlled trials (RCTs) due to difficulties in establishing exactly how the interventions in each group were delivered. In non-randomised studies, a lack of consideration for variations in surgical technique may compromise the full exploration of relationships between exposures and outcomes and result in misidentification of important factors, leading to criticism of the results [1]. This may be particularly problematic in studies that aim to identify risk factors for the 'failure' of a procedure (i.e. recurrence of the same condition, or development of a post-operative complication).

One way to understand the heterogeneity of surgical procedures (within randomised and non-randomised studies) is to record accurately how their constituent components are performed, within case report forms (CRFs). This makes it possible to document variation in delivery of an intervention, and subsequently link this to outcomes, enabling study findings to be contextualised within routine practice. It is therefore crucial that consideration is given to how details of intervention delivery are recorded in CRFs. Whilst it may be impractical to monitor all components of an intervention, it is necessary to provide clarity about which details are expected to be documented and very important information for interpreting study results. Currently, however, methods for identifying the components of interest are lacking and there is a tension between recording all possible surgical components and streamlining data collection. This paper describes the development of novel methods to optimise the documentation of intervention components in CRFs for studies evaluating surgical procedures.

Methods

The research described in this paper was undertaken as preliminary work prior to the CIPHER study (UK Cohort study to Investigate the Prevention of parastomal HERnia), a multicentre cohort study (HTA-14/166/01) aiming to investigate the technical risk factors for developing parastomal hernia (PSH). Technical risk factors can be defined as variations in operative technique that may predispose patients to a PSH. PSH is a common complication following stoma formation surgery and although

For peer review only

various patient-related risk factors have been proposed, technical factors relating to the initial surgical procedure are much less certain. The preliminary work comprised three phases: 1) identification of all of the possible technical factors that may contribute to PSH formation, using literature reviews and mixed qualitative research methods to deconstruct stoma formation into its component parts and steps; 2) amalgamation of these findings into a 'long list' of potential factors influencing PSH development, and 3) rationalisation of the 'long list' into a pragmatic but systematically determined list of final data items to be collected in the CIPHER study.

Phase 1: identifying the technical risk factors for PSH development

Potential risk factors relating to the technical aspects of stoma formation were defined as those that were a) 'known' (defined as reported in the literature, identified from systematic literature searches) and b) 'unreported but practised' (identified using mixed qualitative methods). Methods to identify each type are described in full below.

a) Systematic literature reviews

To comprehensively identify 'known' technical risk factors, snowballing methods were used to find papers cited by and citing prominent articles relevant to the research question. Snowballing was selected as an efficient alternative to conventional search strategies. Database searches using terms such as 'stoma' and 'parastomal hernia' yielded many abstracts, of which only a small proportion were relevant. In contrast, snowballing provided a manageable volume of results and in-depth review of full-text papers to identify all relevant information relating to the intricacies of stoma formation.

Search strategy

Snowballing began using an index paper [2], defined as a high-quality paper known to contain information relevant to the study question. The index paper [3] was selected by two senior surgeons both with extensive experience of surgical research. Papers cited by the index paper were then screened (backward snowballing) using broad inclusion criteria (i.e. written in English and providing technical details of small or large bowel stoma formation). Once backward snowballing was complete, all papers citing the index paper were retrieved (forward snowballing) using a citation tracking service. A further five index papers [4–8] were then selected to continue snowballing. These papers were identified through discussion with experts, with a focus on data-rich, high-quality literature (e.g. reviews). At the end of the snowballing process, two newly published RCTs were identified. Because of their potential relevance, it was agreed they should form part of the literature review but further snowballing was not undertaken [9, 10].

For peer review only - http://bmjopen.bmj.com/site/about/guidelines.xhtml

Data collection and analysis

Searches and analyses of the articles were performed in tandem. Once identified as relevant, papers were uploaded to NVivo 10 (QSR International, Melbourne, Australia). Included papers were read in full to identify all text regarding the technical variations of stoma formation. Once relevant text was identified, a qualitative content analysis was performed and the text was coded into themes, arranged according to the component parts of stoma formation [11]. A proportion of texts were double coded by a second reviewer (LR) to iteratively refine the coding framework. Once saturation was felt to be achieved, a further reviewer (NB) tested the framework by coding further works to identify any additional themes. This was performed twice.

b) Mixed qualitative study

To identify the 'unreported but practised' potential risk factors for PSH, an exploratory study was performed using mixed qualitative methods. This comprised non-participant observation [2] and digital video data capture of stoma formation within the operating theatre, and semi-structured interviews with surgeons and stoma nurses. This work had three objectives, to i) confirm, refine, refute and develop the 'known and reported' factors identified in the literature work, ii) uncover and explore 'unknown but practised' technical variations in stoma formation that may influence PSH development, and iii) triangulate the non-participant observation and semi-structured interview to allow for a more comprehensive understanding of stoma formation techniques [2]. For example, interviews allowed for opportunity to explore and explain the practices observed, and tease out practices that informants reported conducting outside of the observed cases; observations allowed for direct insight into real cases, providing detail that might have been omitted from interviews.

This mixed qualitative study gained NHS Research and Ethics Committee approval from the East Midlands - Nottingham 1 Research Ethics Committee [ref 16/EM/0155] on 1st June 2016. All patients, stoma nurses and surgeons provided written consent to be observed and digitally video recorded in theatre and/or be interviewed. Verbal consent was taken from additional healthcare personnel present in the operating theatre, for observations and digital video recording.

Sampling

Operations featuring stoma formation were purposefully sampled from two NHS hospital centres, with the aim of observing stoma formation performed by different surgeons, rather than the same surgeon creating multiple stomas. The sampling of healthcare professionals was based on a 'key informant' purposeful approach, whereby individuals with exclusive specialist expertise are selected

For peer review only - http://bmjopen.bmj.com/site/about/guidelines.xhtml

as the most productive sample to speak to and observe [12]. Clinicians with a role in conducting stoma surgery or providing aftercare were therefore approached. This included stoma nurses and surgeons performing stoma surgery either routinely (i.e. colorectal surgeons) or as part of the emergency service (i.e. Upper GI and colorectal surgeons), at both consultant and registrar level. Approximately 32 surgeons (including registrars) were performing stoma formation and six stoma nurses were employed at the time of data collection between the two trusts. Sampling continued up until the point of saturation, whereby during parallel analysis no new themes were uncovered and additional data were not adding any further explanatory or descriptive benefit. The decision about when to cease further sampling/data collection was guided by the core research team's (CM, LR, NB) assessment of the 'tipping point', whereby further interviews were not deemed unlikely to be of global benefit for fulfilling the study aim and objectives. This was similar to what others have referred to as the point of 'informational redundancy' [13]: a point at which "new data tend to be redundant of data already collected" [14]. In this case, we reached a point whereby no new items were emerging from concurrent analysis of interviews. While there was a possibility of this changing with further data collection, the team made a judgment to cease, once the benefits of continued sampling were weighed up against the consequences of delaying the next steps of the mixed-methods project.

Data collection

Non-participant observations of stoma formation were undertaken by CM (a medically qualified researcher) and documented as field notes. Observations were supplemented by digital videos of the procedure, filmed by theatre staff or a member of the medical illustration team using portable video recorders in conjunction with above-head theatre cameras (where available). Recording started when stoma formation began and stopped upon completion, when the stoma bag was attached. All recordings were stored on encrypted and secure devices.

Semi-structured interviews were conducted by CM using a topic guide. The topic guide was a series of open-ended questions and prompts/probes, arranged into broad topics to guide the interviewer [11]. Informed by literature review findings, the topic guide was pre-tested in two interviews (not included in the analysis) to test for flow, relevance and whether the questions provoked discussion. Interview topics derived from the category themes identified during the literature review and were updated iteratively as new themes emerged during the interviews. All interviews were digitally audio-recorded and transcribed verbatim. Interviews with surgeons were held directly after an observation wherever possible, to add explanatory weight to the practices observed and explore inconsistencies between what was observed and what was said. Due to time constraints this was sometimes not possible, and instead interviews were held at a mutually convenient time.

For peer review only - http://bmjopen.bmj.com/site/about/guidelines.xhtml

Data analysis

Efforts were made to analyse the data immediately after each interview or observation was completed. This enabled emerging findings to be explored further and scrutinised in the interviews and/or observations that followed. Field notes from observations were aligned with each digital video and watched in full, unedited, by two researchers (CM and NB) independently. The researchers took notes on the videos by documenting the surgeon's movements to create a stepwise account of the surgery, which was imported into NVivo. Semi-structured interviews with surgeons and nurses were transcribed verbatim and also imported into NVivo.

All transcripts were analysed thematically by coding, line by line, to construct themes relating to technical variations in stoma formation. This was an iterative process. CM and LR coded a sample of the transcripts in the early stage of the analysis and met to discuss their coding and agree a consistent framework. LR scrutinised subsequent coded transcripts and CM, LR and NB met regularly to discuss data interpretation and evolution of the coding framework.

Phase 2: Creating a long-list of potential risk factors for PSH

Findings from the literature review and mixed qualitative methods were amalgamated by comparing and contrasting emerging themes. Two researchers, including one senior general surgeon (NB), reviewed each theme and compared the findings of the two data collection processes. The wording of themes and organisation into categories was iteratively refined, combining similar themes and retaining distinction where relevant. Some themes were reclassified as non-technical factors, such as pre-operative bowel preparation. This was a long and iterative process as the coding framework from both phases interacted and created more complexity within the framework of overarching themes, category themes, and themes (Figure 1). Themes did not always use the same language and were difficult to untangle for the more complex concepts. For example, many surgeons referred to the 'Modified Sugarbaker' technique as 'Sugarbaker' in the interviews, whereas in the literature the two were distinct with different meanings. After removing duplicates, refining the description of the themes and creating exhaustive response categories, a 'long list' of discrete risk factors was compiled, organised sequentially according to the steps of stoma formation.

<Insert Figure 1>

Phase 3: Agreement of the final list of data items to be collected in the CIPHER cohort study

A consensus meeting was held to achieve agreement about the final list of data items to be collected. Participants were selected using a key-informant approach, where participants were deliberately

For peer review only

selected for their expertise as either appointed colorectal surgeons or for their experience in research and publication in the field of parastomal hernia prevention, to represent a broad spread of views. Trial managers were invited to provide views on the practicality of recording information (e.g. measurement of adequate bowel mobilisation). Each risk factor in the 'long list' was introduced by the meeting facilitator and discussed in turn. Attendees were encouraged to consider whether each data item was 'essential', 'desirable' or 'not required' for inclusion in the CIPHER CRFs. Consideration was also given to the wording of each item, to ensure there was no ambiguity to the data items and that the response categories were mutually exclusive. When required, researchers (NB and CM) supplemented discussions with information about the origin of each data item (e.g. from qualitative work or reported in the literature). In the case of items reported in the literature, they also described strength of evidence (e.g. from a systematic review, RCT or case series).

If consensus (i.e. unanimous agreement) was achieved about whether an item was 'essential' or 'not desirable', the facilitator moved onto the next item for discussion. Items where consensus was not met or those considered 'desirable' were carried forward to the next 'round' of discussion, when they were reconsidered in light of how all other items had been prioritised.

Further meetings with the trials design team were held to map the items prioritised and refined through consensus using design principles for CRFs.

Patient and public involvement (PPI)

PPI work was central to the design and conduct of the CIPHER study. During conception of the project, it was discussed at the Association of Coloproctology of Great Britain and Ireland Patient Consultation Exercise (attended by representatives from national patient support groups in) and the study team met separately with patient representatives from colostomy, ileostomy and urostomy organisations. Two patients are members of the Study Steering Committee. Items relating to the seniority of surgeon forming the stoma, and whether they were supervised, were of particular interest to patients attending the PPI meeting and they agreed strongly that these should be included in the CRFs.

Results

Phase 1: Identifying the risk factors for PSH development

a) Literature review

Data saturation was achieved after undertaking forward and backward snowballing of six index papers (Table 1). A total of 480 references were screened and of these, 130 articles finally included with a

For peer review only - http://bmjopen.bmj.com/site/about/guidelines.xhtml

publication date ranging from 1958 – 2016. Within these articles, 138 items relating to the technical aspects of stoma formation, hypothesised to influence PSH prevention, were identified.

For peer review only

For peer review only - http://bmjopen.bmj.com/site/about/guidelines.xhtml

Table 1: Approach taken to literature snowballing

Index paper	Description of index paper	References forward snowballing	References backward snowballing	Articles excluded	Articles not located	Duplicates	Articles eligible	Additional articles included in the review (excluding duplicates)
Shabbir, 2012 [3]	Systematic review	34	42	12	1	0	63	42
Aquina, 2014 [4]	Review article	108	13	12	1	15	93	38
Hauters, 2016 [5]	Prospective cohort study	29	15	1	0	19	24	10
Prudhomme, 2016 [6]	Randomised control trial	30	0	0	0	19	11	6
Hotouras, 2013 [7]	Systematic review	115	42	12	6	37	102	26
Hardt, 2013 [8]	Systematic review	52	9	4	3	29	25	6
Additional articles suggested by experts: Brandsma, 2016 [9]& López-Cano, 2016 [10]	Randomised control trials	2	0	0	0	0	0	2

For peer review only - http://bmjopen.bmj.com/site/about/guidelines.xhtml

For peer review only

b) Mixed qualitative methods

Six procedures involving stoma formation were observed (Table 2). The ages of patients whose stoma formation was observed ranged from 31-84 years. Thirteen health professionals were interviewed across two centres (Table 3). No surgeon that was approached declined an interview. However, it was not possible to interview four willing surgeons due to their conflicting time commitments. Six stoma nurses were eligible for inclusion, three consented and none further were approached. Interviews were conducted by CM who was acquainted with three of the surgeons interviewed. Interviews lasted between 17:22 and 1:13:95 minutes (mean = 27:45 minutes). There was a broad range of experience across the clinicians interviewed and consensus meeting participants: their length of time in role ranged from <1-16 and <1-17 years, respectively.

The qualitative work identified 150 items relating to technical factors that were thought to influence the development of PSH.

Table 2: Characteristics of the non-participant observation patient sample

Sex	Ethnicity	Type of stoma formed	Surgical approach to stoma formation	Indication for stoma formation	Planned or unplanned surgery	Centre number
Male	White British	End colostomy	Laparoscopic	Bowel management for paraplegia	Planned	1
Male	White British	End colostomy	Laparoscopic	Bowel management for multiple sclerosis	Planned	1
Female	White British	End colostomy	Open	Bowel cancer	Planned	2
Female	White British	End Ileostomy	Laparoscopic	Inflammatory Bowel Disease	Planned	1
Male	White British	End Ileostomy	Converted laparoscopic to open	Bowel cancer	Planned	2
Male	White British	End colostomy	Open	Sigmoid volvulus	Unplanned	2

For peer review only - http://bmjopen.bmj.com/site/about/guidelines.xhtml

Table 3: Healthcare professional interviewee characteristics

Sex	Specialty	Grade/role
Female	Upper gastrointestinal	Consultant
Female	Colorectal	Stoma nurse
Female	Colorectal	Stoma nurse
Male	Colorectal	Consultant
Male	Upper gastrointestinal	Consultant
Male	Upper gastrointestinal	Consultant
Male	Colorectal	Consultant
Male	Colorectal	Consultant
Male	Colorectal	Consultant
Male	Colorectal	Registrar
Male	Colorectal	Consultant
Female	Colorectal	Stoma nurse
Male	Colorectal	Consultant

Phase 2: Amalgamation of Phase 1 findings into a long-list of data items

Following amalgamation of the themes for the Phase 1 data collection processes and exclusion of duplicates, 180 technical factors were identified (40 were unique to the literature, 63 unique to the qualitative work, 77 appeared in both). This long-list of individual data items was arranged into tables to present the data in a format that was easy to manage for the consensus meeting. Formatting of the long-list into tables resulted in the splitting and condensing of some data items as well as the addition of further response categories such as 'other' where appropriate. The long-list was reviewed by two senior colorectal surgeons from different centres who were not attending the consensus meeting. This was to check for the researchers' understanding of terminology and also to ensure that all factors had adequately been identified. This review resulted in the addition of 12 new data items that were felt to be of interest. A total of 207 data items were taken to the consensus meeting for discussion (a segment of the long-list is provided in Additional file 1, Appendices, Table 1).

The data items were arranged into eight overarching category themes: i) surgical approach to stoma formation; ii) trephine formation (skin and subcutaneous tissue, muscle layers, other); iii) reinforcing the stoma trephine with mesh; iv) closure of the lateral space; v) use of the stoma as a specimen extraction site; vi) stoma snugness; vii) closure of other wounds formed during the procedure; viii) creating and securing the stoma lumen. Each overarching category theme is discussed in greater detail

For peer review only - http://bmjopen.bmj.com/site/about/guidelines.xhtml

below using examples from the qualitative work to demonstrate their reported significance to parastomal hernia prevention.

i) Surgical approach to stoma formation

Findings from both the literature and qualitative work suggested several aspects of the surgical approach to stoma formation may influence the development of PSH. Many variations to operative technique were identified in the literature, with further depth and detail added by the qualitative work. These included: the overall approach to stoma formation (open, laparoscopic, mixed); the section of bowel used to create the stoma; the length of bowel mobilised; whether the stoma site was 'pre-marked'; and the route of the stoma through the abdominal wall (Table 4).

Table 4: Examples from the qualitative data representing the development of the overarching category theme *'Surgical approach to stoma formation'*

Sub theme	Extract
Surgical approach to stoma formation	*Extract 1:"I would favour using a laparoscopic technique if that was technically possible. The reason being that you can actually confirm the anatomy so you can perform an end colostomy, which I think reduces the risk of parastomal formation and the other complications such as prolapse and retraction. You can actually mobilise the colon to bring it up to the abdominal wall."[HCP: BRI0022, Surgeon Lower GI, RDE]* *Extract 2: "There is talk, that maybe sometimes laparoscopic surgery they end up with more hernias, but to me I think it's when they've had a full-blown laparotomy and then they have a stoma form. I think they're probably more at risk because they've got double… They appear to have weaker muscles, don't they?" [HCP: BRI0003, Stoma Nurse, BRI]*
The section of bowel used to create the stoma	*Extract 3: "It does, you can do end stoma rather than a loop. The loop ones I think we tend to have a lot more trouble with prolapse, retraction and herniation because you have to make a bigger cut to bring up the loop of the colon." [HCP: BRI0022, Surgeon, Lower GI, RDE]* *Extract 4: "An end seems less likely; a loop ileostomy seems more likely to get a parastomal hernia than an end ileostomy. I'm trying to think if colostomies seem less likely to get an earlier-on hernia and more likely to get a later-on hernia." [HCP: BRI0002, Stoma Nurse, BRI]*
Length of bowel mobilised	*Extract 5: Interviewer: "What do you think about the amount of bowel mobilised? Do you think that would make a difference to parastomal hernias?" Respondent: "Probably. Yes, you're probably right, probably more with stoma prolapse or retraction, rather than parastomal hernias. I suppose if you have a prolapsing stoma, it would widen the defect. I don't know." [HCP: BRI0009, Surgeon, Upper GI, BRI]*

For peer review only - http://bmjopen.bmj.com/site/about/guidelines.xhtml

Pre-marked stoma site	*Extract 6: "The stoma site is pre-marked at two sites above and below and left lateral to the umbilicus. Both sites have been tied with a suture." [Observation CM: BRI0014, End colostomy, Laparoscopic, BRI]*<br>*Extract 8: Interviewer: "You also mentioned preoperative marking of the stoma site. Do you think that will make a difference to the parastomal hernia rates?"*<br>*Respondent: "It probably doesn't, it is probably more about having a better site for the patient in terms of a place where they can change it. I suppose sometimes if it is marked in a position that is not ideal for a surgeon it can be more challenging for us. If anything is more challenging you might increase things marginally, but probably not." [HCP: BRI0022, Surgeon, Lower GI, RDE]*
Route of the stoma through the abdominal wall	*Extract 9: "Technical factors associated with parastomal. So, I suppose one thing to address is whether we do this as a trans-peritoneal, or an extra peritoneal approach. So, years ago, in the '60s and '70s, and maybe even more recently, it was quite common for the stomas to be tunnelled, pre-peritoneally, laterally, so essentially what you were doing is you'd have the bowel up laterally against the abdominal wall or the under surface, and then it would come out through the muscles as an extraperitoneal stoma. That may have an impact, I don't know, that's never been subjected to a randomised trial comparing it to the trans-peritoneal approach, where the bowel simply just comes through the abdominal wall without tunnelling it. So I think that's a possible surgical technique factor." [HCP: BRI0004, Surgeon Lower GI, BRI]"*

ii) *Trephine formation:*

The term 'trephine' describes the hole or opening in the abdominal wall for the stoma to pass through. Trephine formation was divided into three subsections: skin and subcutaneous fat; muscle layers; and other.

a) Skin and subcutaneous tissue

All the variations in this theme were identified in the non-participant observations, with some being common to the literature and and/or the semi-structured interviews. Various techniques were employed by surgeons to form the trephine of the skin, including the instrument used to create the incision as well as size and shape of the incision (e.g. See Additional file 1, Appendices, Table 2, Extracts 1 and 2). How the trephine was formed through the subcutaneous tissue was also raised as a possible technical factor that could impact on PSH rates. Variations involving the subcutaneous tissue included whether adipose tissue was excised or divided, and (if excised), whether this was done in the shape of a cone or column (See Additional file 1, Appendices, Table 2, Extract 3-6).

b) Muscle layers

For peer review only - http://bmjopen.bmj.com/site/about/guidelines.xhtml

Both the literature and mixed qualitative methods identified multiple variations relating to trephine formation through the abdominal muscle layers. Many were uniquely found in either the literature or the qualitative work. Overall, both the literature and qualitative work indicated that factors surrounding the muscle trephine may be important for PSH prevention. Sub-themes within this theme included the location of the muscle trephine, methods used to make the incision through the anterior rectus sheath, posterior rectus sheath, how the stoma trephine was created through the muscle fibres and methods to measure and dilate the stoma trephine. Interviews revealed different surgical preferences for where the trephine should be created, with some reporting that trephine formation within the rectus sheath was superior for PSH prevention, whilst others were unsure of the significance of the trephine location. Informants also described diverse practices for how the trephine was formed through the different layers of the abdominal wall. Some described using circular incisions to incise the anterior and posterior sheath. This was thought to prevent PSH through an improved distribution of tensile forces (See Additional file 1, Appendices, Table 2, Extract 11). Others preferred a cruciate incision to create the trephine with a 'minimal defect' (See Additional file 1, Appendices, Table 2, Extract 12).

c) Trephine formation: Other

This overarching category theme related to intraoperative nerve injury and factors relating to laparoscopic procedures, such as level of gas inflation during the trephine formation and use of the stoma trephine as a port site. The sub-themes specific to laparoscopic procedures were identified solely in the qualitative work, particularly the semi-structured interviews. As shown in the extract below, the level of inflation and use of the stoma site as a port-site were anticipated by some to impact on PSH occurrence:

Extract 1: "I would generally deflate, I think it's important to deflate the abdomen before making a stoma cut…so from what we've talked about so far, what you want is a hole that's closely mirrors the diameter of the bowel so not too much so that you can get a parastomal hernia, not too little so that it… you're gonna get it pinched. So, to create that doing it under tension with the CO2 inside pushing up against the fascia just doesn't make sense to me."

Extract 2: Interviewer: "I've seen people perform laparoscopic surgery through the stoma site…so will use a [Alexis] wound protector and a glove; do you think that will have an affect on parastomal hernia rates?"

Respondent: "Yeah I think exactly the same reason so I don't do it. Nice idea I know people are desperate to keep their wounds down, but if you've got a sub total colectomy for a permanent

For peer review only - http://bmjopen.bmj.com/site/about/guidelines.xhtml

ileostomy which is with the patient for the rest of their life, I think you're gonna get a hernia and they would probably, I have never actually done it with anyone but they would probably narrow the stoma hole down later and they're using stitches, and yet we've said that repairing a parastomal hernia with stitches has got a 100% recurrence rate" [HCP: BRI0036, Surgeon, Lower GI, BRI]

iii) Reinforcing the stoma trephine with mesh

The use of mesh to reinforce the stoma site at the primary stoma formation was an overarching theme throughout the literature and qualitative work with both sources providing rich data. The use of mesh was a commonly published topic; however, the qualitative work demonstrated uncertainty on its value:

Extract 1: "I put an intraperitoneal mesh to stop the parastomal hernia. That is my anecdotal experience, I've done 10 or 15 of those and I think they are less likely to get hernias, but even if they get hernias, they're less likely to be symptomatic." [HCP: BRI0010, Lower GI, BRI]

Extract 2: "I've seen meshes eroding through the bowel, so I'm reluctant to use it." [HCP: BRI0010, Surgeon, Lower GI, BRI]

There were also variations in practice and views in relation to the type of mesh, its size and shape, location, positioning, the size and shape of trephine through the mesh and how the mesh was secured (See Additional file 1, Appendices, Table 3).

iv) Closure of the lateral space

Closure of the lateral spaces was referred to in both the literature and the semi -structured interviews as a method of PSH prevention. One surgeon theorised that a method to close the lateral space could influence PSH formation by closing a space where a hernia could form, while others were unsure:

Extract 1: "I think having a permanent one, potentially closing the space, you know, probably in the short term, you know, before a bit of bowel has a chance to sort of get its foot through the door metaphorically, after you're closing that space off so there's nothing that can sneak around there while you're waiting for a bit of fibrosis to form, makes sense to me." [HCP: BRI0032, Surgeon Lower GI, RDE]

Closure of the lateral space was performed through 'fascial fixation', the act of attaching the stoma, either the bowel wall (serosa) or the mesentery (the bowels attachment to the posterior abdominal

For peer review only - http://bmjopen.bmj.com/site/about/guidelines.xhtml

wall where its' blood supply derives from) to the anterior abdominal wall [15]. Fascial fixation was discussed in a portion of the semi-structured interviews, and was occasionally performed by two surgeons within their clinical practice (Extract 1 and 2 below), but not others. Sub-themes within this category included fixation of the mesentery to the abdominal wall and fixation of the stoma to the abdominal wall:

Extract 1: "The other thing maybe is whether or not you choose to [tack] the stoma, or choose to tack the bowel to the fascia. So, in does that somehow reduce your risk of parastomal hernia formation? Again, I don't know… I would suture the serosal surface. That's another thing; I don't do any tacking of the mesentery to anything, it's just literally bowel wall." [HCP: BRI0004, Surgeon Lower GI, BRI]

Extract 2: "I suture the colon to the rectus and to the skin separately…" [HCP: BRI0009, Surgeon, Upper GI, BRI]

v) Use of the stoma as a specimen extraction site

Use of the stoma trephine as an extraction site (where the stoma trephine was used to remove the excised piece of bowel) was an overarching category theme only identified through semi-structured interviews and was observed in the non-participant observation:

Extract 1: "The specimen is handed out. With the specimen removed, I can now see that all that bowel did seem to come out of the stoma hole. Impressive! Won't that have stretched it?" [Observation NB: BRI0033, End Ileostomy, Laparoscopic, RDE]

Some surgeons interviewed felt strongly that this would be a high-risk factor for PSH development due to the stretching and extending of the stoma trephine size to accommodate the delivery of the often diseased bowel (see extracts below). Other sub themes included in this overarching category theme were the type of specimen (colon or small bowel) that was brought through the stoma trephine and adjustments to the trephine size to facilitate delivery of the bowel. These factors were also proposed to influence the likelihood of PSH, as shown below:

Extract 2: *"What I feel strongly about is when you use the stoma site to extract a specimen you do predispose them to parastomal hernias so if you're doing a, predictly (sic) if you're doing a subtotal colectomy which is the way it's tempting to do it, you're taking a diseased colon out through a hole which eventually is gonna become an ileostomy site and yes you can narrow it down with sutures afterwards but if we know that parastomal hernia repairs, repair by these sutures have an*

For peer review only - http://bmjopen.bmj.com/site/about/guidelines.xhtml

almost 100% recurrence rate then it's gonna get a parastomal hernia… I think it is important… you're making a hole to get the colon out which is um you know much bigger than the small bowel you're gonna leave there eventually. So technically if you do make the hole too big you're get a parastomal hernia there's no doubt about it." [HCP: BRI0036, Surgeon, Lower GI, BRI]

Extract 3: *"You then have to create a very large hole for the specimen to come out of, and, as we discussed before, the size of the hole… Once you've made it big, making it smaller again is really challenging."* [HCP: BRI0023, Lower GI, RDE]

Extract 4: "The other thing is whenever I, if I ever widen the trephine to deliver a specimen, I then to tend to put a couple of stitches in to try and narrow it again, but I don't think that's as good as not opening it in the first place, if that makes sense." [HCP: BRI0032, Surgeon Lower GI, RDE]

vi) Stoma snugness

When the bowel is being brought through the stoma trephine to form the stoma, it may require further adjustments following an assessment of how 'snug' the bowel is within the stoma trephine- a term used by some interview participants to describe the fit of the stoma in the abdominal wall (Table 5, Extract 1). During the non-participant observations, it was observed that one of the surgeons assessed for *'snugness'* by inserting a finger into the stoma trephine and making a further incision to the abdominal wall (Table 5, Extract 2). This was also discussed in the interviews (Table 5, Extracts 3). A further subtheme within this overarching category theme was mesentery stripping, where one surgeon theorised excessive removal of the stoma's mesentery could cause ischemia and therefore predispose to PSH (Table 5, Extract 4).

Table 5: Examples from the qualitative data representing the development of the overarching category theme *'stoma snugness'*

Sub theme	Extract
Assessment of stoma snugness	*Extract 1: "It is better to pull it through and think, "That is a bit snug." Then making a bit of a nick in the posterior sheath to make it a bit wider or using your Langenbeck's to sometimes just stretch it. That does vary between whether you do that sharp or blunt." [HCP: BRI0022, Surgeon, Lower GI, RDE]*<br><br>*Extract 2: "With the surgeons index finger he checks for snugness of the trephine. On deciding it is too tight he requests a langenbeck retractor and places it along side his finger, asking the SpR to hold it and retract. Using mackindo scissors he makes a further incision in the peritoneum. Consultant re-checks for snugness on either side of the stoma, checking from inside of*

For peer review only - http://bmjopen.bmj.com/site/about/guidelines.xhtml

	the abdomen and outside." [Observation CM: BRI0034, End Ileostomy, Converted laparoscopic to open, BRI]<br><br>*Extract 3: "The traditional has been one finger in, one finger in, and a bit of pulling and tearing and everything else." [HCP: BRI0023, Lower GI, RDE]*
Mesentery stripping	*Extract 4: "What you're trying to avoid is stripping the mesentery off, cos if you strip the mesentery off the bowel it's ischemic and it will go dusky and flat so you might create a bigger whole and then you've got a risk factor for parastomal hernia." [HCP: BRI0036, Surgeon, Lower GI, BRI]*<br><br>*Extract 5: "If the appendices epiploicae are large I'll remove them but I want it under no tension and subcuticular to extramucosal sutures with all the knots buried." [HCP: BRI0015, Surgeon, Lower GI, RDE]*

vii) Closure of other wounds formed during the procedure

Closure of other abdominal wounds formed during the procedure was identified as themes in both the literature and qualitative work. The qualitative work provided rich data on the importance of closing the other abdominal incisions for PSH prevention. For example, BRI00023 felt strongly that restoring the anterior abdominal wall's function through closure of the deep layers using small bite closure with a specific suture size and type was superior for PSH prevention (Table 6, Extract 1). A further sub-theme identified was the order of the wound closure. Some interviewees stated their preference for closing the anterior abdominal wall incisions prior to securing the lumen of the stoma, this practice was also confirmed in the observations (Table 6, Extract 3 and 4).

Table 6: Examples from the qualitative data representing the development of the overarching category theme *'closure of other wounds formed during the procedure'*.

Sub theme	Extract
Layers of wound closure (Deep layers; Skin layers)	*Extract 1: "How you close the abdominal wall I think is really important, because it then affects how likely the patient is to develop an incisional hernia. If the patient develops an incisional hernia that will impact, because of the mechanics of the abdominal wall on the stoma aperture and then lead to development of parastomal hernias. The two are intimately related. You have to take every possible step to ensure that you have good abdominal wall closure, and restoration of appropriate function, so we tend to use the small bite closure technique using 2-0 PDS delayed absorbable sutures. It's been standard practice now for about two years, particularly for primary surgery." [HCP: BRI0023, Lower GI, RDE]*

For peer review only - http://bmjopen.bmj.com/site/about/guidelines.xhtml

	Extract 2: "Typically I would use a glue to give you a seal. Then you haven't got a dressing extending from the edges of the main wound, that might impinge on where your stoma bag would sit. Also the glue, even if there are leakages, will give you a seal over the centre of the wound." [HCP: BRI0018, Surgeon, Hepatobiliary, RDE]
Order of wound closure	*Extract 3: "At this point the end of the bowel that I've brought through is typically stapled off, and I will leave it stapled off when it's drawn through the stoma. Then we would finish any further intraabdominal work, close the anterior abdominal wall, close the skin, dress the skin." [HCP: BRI0018, Surgeon, Hepatobillary, RDE]*<br><br>*Extract 4: "4.0 monocryl on curve and PDS sutures close midline umbilical port site. Mostly out of view with the handheld. Monocryl for port smaller lateral port sites. Wet and dry. Glue used (theory to reduce stoma infections). Two babcocks on distal edges of stapled stoma. Out of view. Lotus dissects the stapled line. Fine tooth forceps and 4.0 monocryl on curve secures stoma to skin with slight spout at 3, 12, 9 then 6 o'clock positions." [Observation CM: BR0021, End Colostomy, Laparoscopic, RDE]*

viii) Creating and securing the stoma lumen

How the stoma lumen was created and secured was an overarching category theme identified in both the literature and qualitative work. Subthemes included efforts to reduce faecal spillage and cleaning of the stoma lumen. Further detail was provided, particularly from non-participant observations, which identified multiple methods of suturing the stoma lumen. It was not clear if interview participants perceived these factors to affected PSH rates, but there were many different reported and observed practices to creating and securing the stoma lumen.

Phase 3: Agreement of the final list of data items to be collected in the case report forms

Consensus methods

A consensus meeting was held with eight professionals, chaired by the CIPHER study's Chief Investigator (Table 7). Decisions were made through discussion and voting. Whilst consensus on the classification of most data items was simple, others proved more difficult. Some items such as 'length of bowel mobilised' were agreed to be important, but discussion raised concerns over the difficulty in obtaining intraoperative measurements as well as the reliability of surgeons' self-reporting. This item was therefore classified as 'undesirable'. For data items where consensus was unclear, the panel took

For peer review only

an open vote by raising their hands. While this method resolved some of the conflict, for a small proportion of the data items, disagreement persisted. These data items were classed as 'desirable' and re-discussed at the end of the meeting. For 15 data items, further discussion did not resolve the differing views and it was decided that these data items would remain 'desirable' and would be provisionally retained, with a final decision to be made during design of the CRFs.

Table 7: Characteristics of the consensus meeting participants

Gender	Specialty	Grade	Trust
Male	Colorectal	Consultant	Royal Devon and Exeter
Male	Colorectal	Consultant	Queen Elizabeth Hospital
Male	Colorectal	Consultant	Bristol Royal Infirmary
Male	Colorectal	Consultant	Royal Surrey County Hospital
Female	Colorectal	Specialty Trainee (ST7)	Southmead Hospital
Female	Professor of Surgery	Consultant	Bristol Royal Infirmary
Male	Professor of Health Services Research	Non-clinician	Bristol Royal Infirmary
Male	Health services provider	Non-clinician	Bristol Royal Infirmary

Four data items that were suggested during the consensus meeting were also included in the final short list: i) name of the primary procedure, ii) indication for surgery, iii) excision of anterior sheath during trephine formation, and iv) excision of posterior sheath during trephine formation. Additional amendments to the list included wording changes, condensing of items, and creating of additional response categories. A total of 207 data items were discussed. These items were categorised as essential (n=56), desirable (n=15) and undesirable (n=136). The undesirable items were deemed not important to collect in CRFs and therefore excluded.

For peer review only - http://bmjopen.bmj.com/site/about/guidelines.xhtml

Constructing the short-list of data items into data fields involved two meetings between the research team, the trials unit staff and the Chief Investigator of the CIPHER study. Due to the number of items deemed 'essential' during the consensus meeting it was decided that the majority of 'desirable' items could not be included in the final CRFs. Each data item was considered individually, and only three of the fifteen desirable items were included in the final CRFs (use of sutures to buttress the end of the trephine anterior sheath; use of sutures to buttress the end of the trephine posterior sheath; damage to epigastric vessel), because they were simple and collecting them would not increase the burden on participating surgeons. This decision was supported by the members of the consensus meeting, who were contacted latterly. Amendments were made to phrasing and formatting of the essential items but the content/meaning of the items remained unaltered. The final number of data items included in the CRFs was 59 (See Additional file 1, Appendices, Table 4). Combining expert opinion and the trials unit staff knowledge of CRF practicalities was key to ensuring the data items were mapped clearly on to the CRFs, enhancing data collection completeness. These collaborative discussions also enabled any disparity between surgeon and trials unit members expectations and any residual ambiguities between the data items to be resolved.

Adherence to the CRFs and any identified associations between surgical components of stoma formation and the risk of developing parastomal hernia will be reported in the results of the main CIPHER study (See Additional file 2, CIPHER Case Report Form Extract).

Discussion

This novel study used a combination of literature reviews, mixed qualitative methods and consensus processes to identify key components of a surgical procedure. This informed development of CRFs for a cohort study investigating risk factors for the development of PSH. A long-list containing 207 data items were identified, which was rationalised to 59 to be recorded in the CRFs (Figure 2: Overview of study). This application of methods has enabled the development of a systematic process for recording how surgical procedures are conducted in clinical studies, resulting in improvements in transparency of intervention delivery and subsequent implementation in practice. The use of scientifically informed CRFs in trials will be valuable to examine protocol adherence and associations between surgical components and outcome.

Previous studies investigating risk factors for the development of PSH have tended to focus on technical variations of interest to the investigators and wider surgical community (e.g. prophylactic mesh placement [16], trephine location [17]). In some, a selection of other technical factors were

For peer review only

standardised within the study protocol, although no commentary or justification was provided for these. A typology of surgical procedures, developed to identify components of surgical interventions within RCTs, could have been employed to facilitate the process of ascertaining how much standardisation is required for each intervention component [18]. However, because this approach was designed for RCTs it does not completely suit non-randomised study designs such as CIPHER. The current study modifies and extends the previously developed methods [18] to incorporate how to develop CRFs to capture the delivery of a surgical intervention within its natural setting, recognising that surgical technique is usually variable.

This is a novel study undertaken to inform the development of CRFs in a detailed and systematic way. Despite this, however, there are potential limitations. Snowballing search strategies are not considered to be as rigorous as systematic database searches of all available literature, tending to favour the identification of well-known literature, and as such there is a risk that the included papers may not represent the full body of existing literature. As this study focused on achieving saturation of themes, we deemed it unnecessary to identify all the existing evidence and instead were able to identify relevant studies in a more streamlined way. Key to this was the thoughtful selection of the index papers to ensure adequate breath of data and testing the data saturation point by consulting experts and providing a further independent reviewer who coded additional papers to test the end point. The authors particularly acknowledge the limitations of the literature review date (2016). We have reviewed key papers [19, 20] published after 2016 which did not yield new themes. Similar to the findings of this study, the authors concluded the lack of consensus on the optimal surgical technique for parastomal hernia prevention to still be present. The methods used to reach consensus can also be criticised. The meeting was chaired by the study CI who theoretically could influence responses, and voting was not anonymous. To mitigate against this, attendees were deliberately selected to ensure a broad range of views were represented.

Inductive qualitative methodological approaches are fundamental to advancing knowledge and insight in a field; these approaches overcome the challenges of becoming constrained by the research team's preconceptions, as is often the limitation of survey methodology. In this project, the qualitative work served this critical role of identifying new items, thus moving the research team beyond the confines of what had already been reported in the literature. It successfully contributed new insights which were incorporated into the final list of items. There is, however, a tension between inductive qualitative methods, principally aimed at generating depth of meaning and understanding, and the intentions underpinning this project: identification of concepts for exhaustive lists. Claims of saturation are difficult in this study, irrespective of how one conceptualises saturation [13]. Unlike qualitative studies that seek saturation of thematic or theoretical constructs (e.g. Grounded Theory

For peer review only

[21]), our intention was to identify and capture as many examples of 'variation' in surgical practice as possible. We reached a pragmatic decision to cease further interviews during analysis, when themes were noted to regularly repeat and no new themes or changes to hierarchies were made to the coding framework. This was the point at which further attempts to interview were deemed to be counter-productive, considering the time/funding constraints, and the subsequent stages of the study. Including more trusts/individuals in the sample might have illuminated more examples of variation. Nonetheless, this does not undermine or diminish the value of the insights that were contributed by the qualitative work, as would be the case in studies seeking theoretical saturation. New items were added following the consensus meeting, but this was to be expected. The consensus meeting was different in its nature and aims, relative to the observations, interviews, and literature reviews. The contributors were explicitly asked to suggest addition of items from the emerging final list, and had been exposed to a wide array of data items that will likely have influenced their thoughts and responses. It was anticipated that list items would be added/removed, by virtue of the task.

This study identified numerous potential technical factors relating to the future development of PSH, highlighting the complexity of surgical interventions and the need for this to be carefully considered in feasibility or pilot work prior to a main study. Similar to another study conducted by the research team, the research team found the key informants were very eager to participate and to share their expert opinions, via observations and interview [22]. Nobody declined to participate and there was no perceived hesitation from anyone approached. In fact, the surgeons and stoma nurses directly facilitated the observations through introductions to appropriate patients and co-ordination with the theatre teams. Willingness to participate further validates the feasibility of performing such work. A further key finding from this study is that unique themes relating to the technical process of stoma formation were identified through both literature and qualitative data collection. Moreover, the broad - and at times conflicting - range of surgical practices/beliefs amongst participating healthcare professionals may represent a lack of evidence and/or awareness of the evidence. Together, these provide strong rationale for undertaking such work prior to a main study, preferably through multiple methods of data collection. The mixed methods approach has enabled the breadth of data items included in the final CRFs to be more comprehensive than if they were based solely on the opinions and interests of the CIPHER study team. Nesting this work within the early stages of the CIPHER study may have helped to reduce the risk of omitting key variations in stoma formation that may affect PSH development. Other studies may find it helpful to build in a similar phase of feasibility work to provide the time and funding to develop CRFs in such detail. Future work may be needed to streamline the development of CRFs in such detail. On reflection the research team can recommend performing the

For peer review only

literature review while awaiting ethic decisions and local approvals and having multiple researchers performing the literature search in parallel to improve efficiency.

This study used multi-modal data collection to enable systematic identification of the components and technical variations of a complex surgical intervention. This informed the rigorous development of CRFs for a future cohort study (CIPHER) aiming to determine risk factors for a key complication of stoma formation surgery (PSH). Through a mixed methods approach, data items were rationalised into a manageable number to include those considered most important. These methods will enable future studies investigating surgical interventions to comprehensively design CRFs to reflect the complexity of the intervention, making it possible to understand exactly how an intervention was delivered within a study so that appropriate conclusions can be drawn.

Author contributions

NB, LR and JB conceived the idea for the study. CM, LR, JB and NB wrote the protocol. CM undertook the literature and qualitative work supervised by LR, JB and NB. NS, ID, TP, JS, TR, JB, JT, JR, BR, and HB all contributed to the consensus meeting. CM, NB, LR and JB drafted the manuscript and all authors read and approved the final manuscript.

Funding statement

This work was performed as part of the NIHR funded UK **C**ohort study to **I**nvestigate the prevention of **P**arastomal **HER**nia (CIPHER) Study: Understanding surgery and current practice in stoma formation and developing Patient Reported Outcome Measures for Parastomal Hernia to inform Phase B of CIPHER. Part A was led by Professor Jane Blazeby and supported by the Bristol-based Clinical Trials and Evaluation Unit (CTEU), Royal College of Surgeons surgical trials centre, the MRC ConDuCT-II Hub for Trials Methodology Research, and the NIHR Biomedical Research Centre at University Hospitals Bristol and Weston NHS Foundation Trust and the University of Bristol. The views expressed are those of the author(s) and not necessarily those of the NIHR or the Department of Health and Social Care. Jane Blazeby is an NIHR Senior Investigator and Natalie Blencowe is an MRC Clinician Scientist.

Competing of interests

The authors declare that they have no competing interests.

Ethics approval

For peer review only - http://bmjopen.bmj.com/site/about/guidelines.xhtml

This study gained National Health Service (NHS) Research and Ethics Committee approval from the East Midlands - Nottingham 1 Research Ethics Committee [ref 16/EM/0155] on the 1st of June 2016. Ethical and local agreements were in place for both NHS trusts prior to commencing the research.

Data sharing

The datasets generated and analysed during the current study are not publicly available due to confidentiality reasons but are available from the corresponding author on reasonable request.

Word count

References:

[1] Avery KN, Barham CP, Berrisford R, et al. Understanding surgical interventions in RCTs: the need for better methodology. *The Lancet* 2013; 381: 27–28.

[2] Ritchie J, Lewis J, Lewis P of SPJ, et al. *Qualitative Research Practice: A Guide for Social Science Students and Researchers*. SAGE, 2013.

[3] Shabbir J, Chaudhary BN, Dawson R. A systematic review on the use of prophylactic mesh during primary stoma formation to prevent parastomal hernia formation. *Colorectal Dis* 2012; 14: 931–936.

[4] Aquina CT, Iannuzzi JC, Probst CP, et al. Parastomal hernia: a growing problem with new solutions. *Dig Surg* 2014; 31: 366–376.

[5] Hauters P, Cardin J-L, Lepere M, et al. Long-term assessment of parastomal hernia prevention by intra-peritoneal mesh reinforcement according to the modified Sugarbaker technique. *Surg Endosc* 2016; 30: 5372–5379.

[6] Prudhomme M, Alline M, Chauvat J, et al. Primary prevention of peristomial hernias via parietal prostheses: A randomized, multicentric study (GRECCAR 7 trial). *Dig Liver Dis* 2016; 48: 812–816.

[7] Hotouras A, Murphy J, Thaha M, et al. The persistent challenge of parastomal herniation: a review of the literature and future developments. *Colorectal Dis* 2013; 15: e202–e214.

[8] Hardt J, Meerpohl JJ, Metzendorf M-I, et al. Lateral pararectal versus transrectal stoma placement for prevention of parastomal herniation. *Cochrane Database Syst Rev*.

[9] Brandsma HT, Hansson BME, Aufenacker TJ, et al. Prophylactic mesh placement to prevent parastomal hernia, early results of a prospective multicentre randomized trial. *Hernia* 2016; 20: 535–541.

For peer review only - http://bmjopen.bmj.com/site/about/guidelines.xhtml

[10] López-Cano M, Serra-Aracils X, Mora L, et al. Preventing parastomal hernia using a modified sugarbaker technique with composite mesh during laparoscopic abdominoperineal resection. *Ann Surg* 2016; 264: 923–928.

[11] Bryman A. *Social Research Methods*. Oxford University Press, 2016.

[12] Marshall MN. Sampling for qualitative research. *Fam Pract* 1996; 13: 522–526.

[13] Saunders B, Sim J, Kingstone T, et al. Saturation in qualitative research: exploring its conceptualization and operationalization. *Qual Quant* 2018; 52: 1893–1907.

[14] Grady MP. *Qualitative and Action Research: A Practitioner Handbook*. Phi Delta Kappa International, 1998.

[15] Fazio VW, Church JM, Wu JS. *Atlas of intestinal stomas*. Springer Science & Business Media, 2012.

[16] Odensten C, Strigård K, Rutegård J, et al. Use of prophylactic mesh when creating a colostomy does not prevent parastomal hernia: a randomized controlled trial—STOMAMESH. *Ann Surg* 2019; 269: 427.

[17] Hardt J, Seyfried S, Weiß C, et al. A pilot single-centre randomized trial assessing the safety and efficacy of lateral pararectus abdominis compared with transrectus abdominis muscle stoma placement in patients with temporary loop ileostomies: the PATRASTOM trial. *Colorectal Dis* 2016; 18: O81–O90.

[18] Blencowe NS, Mills N, Cook JA, et al. Standardizing and monitoring the delivery of surgical interventions in randomized clinical trials. *Br J Surg* 2016; 103: 1377.

[19] Antoniou SA, Agresta F, Garcia Alamino JM, et al. European Hernia Society guidelines on prevention and treatment of parastomal hernias. *Hernia* 2018; 22: 183–198.

[20] Aslam MI, Rubio-Perez I, Smart NJ, et al. A survey on practices for parastomal hernia prevention and repair among ESCP surgeons. *Hernia* 2019; 23: 825–828.

[21] Glaser BG, Strauss AL. *The Discovery of Grounded Theory: Strategies for Qualitative Research*. Transaction Publishers, 2009.

[22] Blencowe NS, Blazeby JM, Donovan JL, et al. Novel ways to explore surgical interventions in randomised controlled trials: applying case study methodology in the operating theatre. *Trials* 2015; 16: 589.

For peer review only

Figure one: Example of the coding framework for mesh trephine size

Figure two: Overview of study

For peer review only

For peer review only - http://bmjopen.bmj.com/site/about/guidelines.xhtml

Figure 1: Example of the coding framework for mesh trephine size

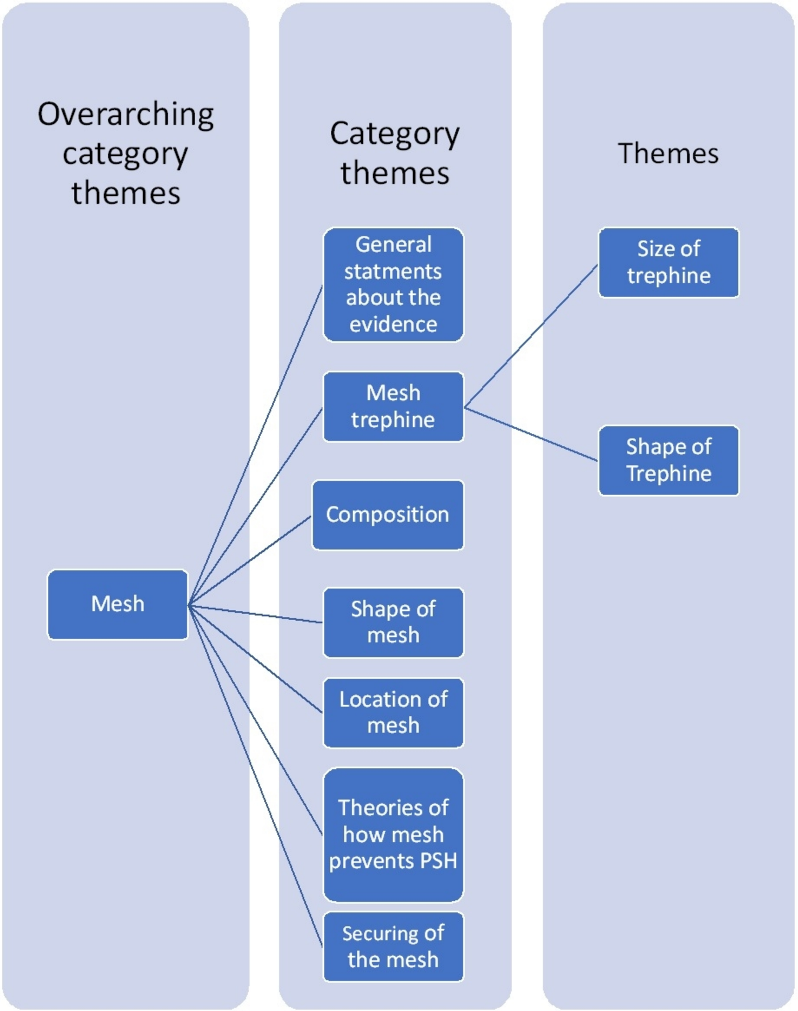

203x222mm (144 x 144 DPI)

Figure two: Overview of study

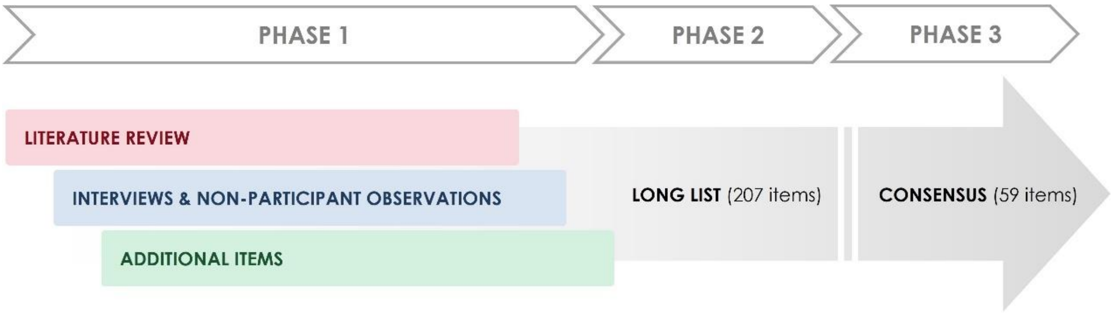

For peer review only

Additional File 1, Appendices

Table 1: Example from the long-list representing the discussion held at the consensus meeting on the overarching category theme '*Reinforcing the stoma trephine with mesh*'.

**Technical variations**		
Reinforcing the stoma trephine with mesh		
**a. Mesh type**	Synthetic	Non-absorbable [polypropylene, polyethylene terephthalate, polytetrafluoroethylen (PTFE), polyvinylidene fluoride (PVDF), other]
		Absorbable [rapid, delayed]
		Combined
	Biologic	
	Other	
**b. Mesh size**	Size of mesh inserted [height and width measured in cm OR if circular widest diameter in cm]	Mesh was uncut/unadjusted
		Mesh not cut/adjusted
	Mesh size estimation	Finger breadths
		Ruler
		Eye
		Other
**c. Shape of inserted mesh**	2D	Oval
		Square
		Circle
		Other
	3D	Funnel
		Other
**d. d. Location of mesh and mechanism of creating space**	Sublay [pre-peritoneal, retro-rectus]	Space created by instrumental dissection
		Space created with blunt force (e.g. finger)
		Combination
		Other
	Onlay [supra-rectus]	Space created by instrumental dissection
		Space created with blunt force (e.g. finger)
		Combination
		Other

For peer review only

<table>
<tr><td rowspan="2"></td><td colspan="3">Inlay [intra-rectus]</td></tr>
<tr><td colspan="3">Underlay [intra-peritoneal, IPOM]</td></tr>
<tr><td rowspan="4">e. Route used to position mesh</td><td colspan="3">Through the main operative incision</td></tr>
<tr><td colspan="3">Through the stoma trephine</td></tr>
<tr><td colspan="3">Via a port</td></tr>
<tr><td colspan="3">Other</td></tr>
<tr><td rowspan="9">f. Mesh trephine</td><td colspan="3">Sugarbaker (mesh edges secured to fascial edges)</td></tr>
<tr><td colspan="3">Modified Sugarbaker (mesh edges secured overlapping the fascial edges)</td></tr>
<tr><td rowspan="7">Key-hole (trephine within the mesh) Y/N</td><td colspan="2">Size of key-hole (widest diameter in cm)</td></tr>
<tr><td rowspan="3">Shape of key-hole</td><td>Circular</td></tr>
<tr><td>Cruciate</td></tr>
<tr><td>Other</td></tr>
<tr><td colspan="2">Key-hole created using a mechanical device (other than the tacker)</td></tr>
<tr><td colspan="2">Key-hole created before mesh has been secured</td></tr>
<tr><td colspan="2">Key-hole created after mesh has been secured</td></tr>
<tr><td rowspan="19">g. Securing the mesh</td><td colspan="3">Not secured</td></tr>
<tr><td rowspan="11">Securing the mesh to the abdominal wall</td><td rowspan="3">Tacking</td><td>Single crown</td></tr>
<tr><td>Double crown</td></tr>
<tr><td>Other</td></tr>
<tr><td rowspan="2">Suturing</td><td>Continuous</td></tr>
<tr><td>Interrupted [entire perimeter, corners only, other]</td></tr>
<tr><td rowspan="2">Suture choice</td><td>Absorbable</td></tr>
<tr><td>Non-absorbable</td></tr>
<tr><td>Glue</td><td></td></tr>
<tr><td colspan="2">Combination of tacks and sutures</td></tr>
<tr><td colspan="2">Mechanical device to secure the mesh (other than tacker)</td></tr>
<tr><td colspan="2">Other</td></tr>
<tr><td rowspan="6">Securing the mesh to the stoma serosa</td><td rowspan="3">Suturing</td><td>Interrupted [number and position]</td></tr>
<tr><td>Continuous</td></tr>
<tr><td>Other</td></tr>
<tr><td rowspan="2">Suture choice</td><td>Absorbable</td></tr>
<tr><td>Non-absorbable</td></tr>
<tr><td colspan="2">Other</td></tr>
</table>

For peer review only

Table 2: Examples from the qualitative data representing the development of the overarching category theme '*Trephine formation: Skin and subcutaneous tissue; Muscle layers; Other*

**Overarching category theme**<br><br>**Trephine formation*: Skin and subcutaneous tissue***	
**Sub theme**	**Extract**
Skin incision at stoma site	*Extract 1: "The surgeon turns the knife 90 degrees and 'shaves through' the skin around the Langenbeck. This makes a circle shaped incision in the skin. They then achieve haemostasis using forceps diathermy." [Observation NB: BRI0021, End Colostomy, Laparoscopic, RDE]*<br><br>*Extract 2: "Holding the skin edges with some forceps, the surgeon uses diathermy to incise each edge (skin and subcutaneous tissue), thereby turning the cruciate incision into a oval." [Observation NB: BRI0034, End Ileostomy, Converted laparoscopic to open, BRI]*
Subcutaneous adipose	*Extract 3: "I used to take out a long column of fat and then I thought, "Well, fat is so compressible, what's the point?" And so I just separate it and cut, you know, in a straight line down to the rectus with some protractors… Well, I'm trying to take away- take away as little as possible, to try and minimise the risk of parastomal hernias. So I don't know if it works or not, but again, it's anecdotal other than evidence-based." [HCP: BRI0009, Surgeon, Upper GI, BRI]*<br><br>*Extract 4: Interviewer: "So you think that may have an effect (on PSH rates)."*<br><br>*Respondent:" Yes…And I think the fatter somebody is the more fat people excise and therefore the bigger the space because the fat retracts around it." [HCP: BRI0015, Surgeon, Lower GI, RDE]*<br><br>*Extract 5: "I would have a cone of fat with a circle of skin on the top." [HCP: BRI0018, Surgeon, Hepatobiliary, RDE]*<br><br>*Extract 6: "No subcutaneous tissue is excised." [Observation CM; RDE, End Colostomy, Laparoscopic, RDE]*

For peer review only

**Overarching category theme** **Trephine formation*: Muscle layers***	
**Sub theme**	**Extract**
Location of the muscle trephine	*Extract 7: "I think it makes a difference [to PSH rates]. If you put the trephine within the rectus sheath then the rectus sheath is a closed compartment because you've got a posterior and anterior layer and lateral and medial. Therefore any rise in the force or the pressure within that rectus abdominal muscle by the passage of stoma, or material through a stoma, is equalised by the fact it's a closed compartment so you get are resistant force. If you put it outside the rectus abdominus, in the obliques, then by the very nature of which your obliques and transverse salus separate then they're more likely to separate around a stoma. I think there's a difference in whether the patient's got a posterior sheath or whether you've got a peritoneum because if you've only got a peritoneum you're less likely to have a resisted force because it's dissipated by the peritoneum at the back as opposed to posterior sheath."* [HCP: BRI0015, Surgeon, Lower GI, RDE] <br>*Extract 8: "I honestly don't know. You could imagine it might [make a difference in PSH rates], but I don't know."* [HCP: BRI0018, Surgeon, Hepatobillary, RDE] <br>*Extract 9: "Patients who have [a stoma] above the arcuate line of Douglas may be less inclined to have parastomal hernias…Disrupting the linea semilunaris I think is a recipe for disaster."* [HCP: BRI0023, Lower GI, RDE]
Approach to creating the trephine through the anterior sheath	*Extract10: "Some people do transverse. Some do longitudinally. Some make cruciate incisions. I've seen people do incisions say either longitudinally or horizontally, and then buttress the ends with suture material, to try and prevent propagation." [HCP: BRI0023, Lower GI, RDE]* <br>*Extract 11: 'By virtue of doing a cruciate incision you are splitting, because you've got a linear incision, it might widen. If we make a circular incision does that mean that the tensal strength or the*

For peer review only

	forces within the abdominal wall, are they going to distribute." [HCP: BRI0004, Surgeon Lower GI, BRI]<br><br>*Extract 12 "The cruciate incision allows the bowel to come through with the minimum defect. So that's why I've avoided doing the circular incision in the rectus sheath." [HCP: BRI0009, Surgeon, Upper GI, BRI]*
Approach to creating the trephine through the posterior sheath/peritoneum	*Extract 13: ""In terms of technique, I think the size of the cruciate incision, both on the anterior and the posterior layers of the rectus sheath, matter. I think that that's probably, I think, what matters most." [HCP: BRI0001, Surgeon, Upper GI, BRI]*
Approach to creating the muscle trephine	*Extract 14: "Respondent: If you de-vascularise any muscle fibres and it becomes infected then infection leads to hernia formation there is no doubt about that.*<br><br>*Interviewer: So splitting of the fibres improves (PSH rates) that rather than incising them?*<br><br>*Respondent: I think it does, I can't show you evidence to say that. Logically to me if you are splitting the fibres and not disrupting my cutting through the fibres and all the blood supply to the muscle and the surrounding area." [HCP: BRI0022, Surgeon, Lower GI, RDE]*
Dilation of the stoma	*Extract 15: "Further fibres of muscles/fascial tissue are cut with diathermy" [Observation NB: BRI0021, End Colostomy, Laparoscopic, RDE]*
How the measurements of the stoma trephine are made	*Extract 16: "Well, it's very subjective. Some people would say two fingers, but my two fingers are bigger than your two fingers." [HCP: BRI0010, Surgeon, Lower GI, BRI]*
**Overarching category theme**<br><br>**Trephine formation*: Other***	

For peer review only

Sub theme	Extract
Intraoperative nerve damage	*Extract 17: "I think if you start making incisions I think you're more at risk at damaging muscles- Not muscles, the nerves that run into the abdominal wall." [HCP: BRI0004, Surgeon Lower GI, BRI]*

Table 3: Examples from the qualitative data representing the development of the overarching category theme '*reinforcing the stoma trephine with mesh*'

***Overarching category theme: '*Reinforcing the stoma trephine with mesh'**	
**Sub theme**	**Extract**
Mesh type	*Extract 6: "See, the fact that all these different meshes are being used for parastomal hernias must mean that no one of them is actually working. The more ways there are of doing something, the more sure you are that nobody knows what the right way is." [HCP: BRI0009, Upper GI, BRI]*<br><br>*Extract 7: "The corners are trimmed off the mesh, making an oval shape. It looks like it's made of polypropylene."* [Observation NB: BRI0021, End Colostomy, Laparoscopic, RDE]<br><br>*Extract 8: Interviewer: "Why do you think Permacol's [biologic mesh] beneficial?"*<br><br>*Respondent: "Compared to [synthetic] mesh? Just because it resists infection better."* [HCP: BRI0032, Lower GI, BRI]<br><br>*Extract 9: "I would use in most patients a piece of polypropylene mesh in the retrorectus position…With the synthetic meshes the rate of tissue ingrowth is so rapid, and scar tissue formation is so quick, that you will find that you will have a fairly solid ring of tissue within four to six weeks, so that you get the protective effect relatively quickly. It's why most of the RCTs would demonstrate nowadays a protective effect with prophylactic mesh."* [HCP: BRI0023, Lower GI, RDE]
Mesh size	*Extract 10: "The consultant measures (using a ruler) the width and length of the retrorectus space and draws a rectangle in permanent marker of the edge of the mesh. The SpR holds the mesh while the consultant cuts*

For peer review only

	using scissors." [Observation CM: BRI0030, End Colostomy, Open, BRI]<br><br>*Extract 12: "If we do that it involves developing a plane between the muscle layer and the posterior sheath and placing a very small piece of mesh 4cm by 4cm with polypropylene which you pull the stoma through."* [HCP: BRI0022, Lower GI, RDE]
Mesh shape	*Extract 13: "The corners are trimmed off the mesh, making an oval shape. It looks like it's made of polypropylene…The surgeon folds the mesh into quarters, then cuts off the corner, which makes a circular shaped hole in the centre.* [Observation NB: BRI0021, End Colostomy, Laparoscopic, RDE]
Location of mesh	*Extract 14: "The consultant then places the mesh with the retrorectus space."* [Observation CM: BRI0030, End Colostomy, Open, BRI]<br><br>*Extract 15: "Whether that is done intra-peritoneal, whether that's done in a retroperitoneal space, or in an onlay space… So, what I'm saying to you is there's about ten different ways of doing something, which to anyone says that none of them are perfect, and that's never really been subjected to a trial of sufficient power, strength for us to know what the most effective technique is."* [HCP: BRI0004, Lower GI, BRI]<br><br>*Extract 16: "I think the evidence when they did all those prophylactic meshes, whether you put it intraperitoneal, retromuscular or anterior into the muscles, it didn't show any difference in the outcomes. I think it's just strengthening the muscle layer, but probably a large trial will tell us whether there's any difference. In an ideal world, I think you should probably put it either intraperitoneal or retromuscular. I think putting it anterior to the rectus sheath is probably pointless, you might as well not put it, I don't think it adds anything to it because the hernias start from the inside…Yes, it will just lift the mesh; it's not going to make any difference. It either has to be between the rectus and the posterior sheath or intraperitoneal where it might have a use."* [HCP: BRI0010, Lower GI, BRI]

For peer review only

How the space is created and method of positioning the mesh	*Extract 17: "Single- index finger feels and sweeps around breath of trephine to create a space in the retrorectus space."* [Observation CM: BRI0021, End Colostomy, Laparoscopic, RDE]<br><br>*Extract 18 "The surgeon uses their finger to create a space, and further fibres of muscles/fascial tissue is cut with diathermy…The surgeon uses a clip to guide the mesh into the pocket that they have created for it. They use their fingers to smooth the mesh out and get it into position."* [Observation NB: BRI0021, End Colostomy, Laparoscopic, RDE]
Mesh trephine	*Extract 19: "But I think, if you're putting a keyhole into any mesh, in mesh prophylaxis, or even as a repair, it's just going to shrink away, that circle is just going to get larger…*<br><br>*Interviewer: "So it contracts… Do you think that's a reason why the Sugarbaker technique would be superior to-"*<br><br>*Respondent: "Yes, so that's what I think, why Sugarbaker would be superior. That said, I don't think it's 100% effective. That's what I would use as a method of mesh prophylaxis and permanent stoma."* [HCP: BRI004, Lower GI, BRI]<br><br>*Extract 20: "If you've got a circular incision maybe you've got a bit more strength around the radius of it, rather than splitting it."* [HCP: BRI0032, Surgeon Lower GI, RDE]
Method of securing the mesh	*Extract 21: 'When I do it laparoscopically I just use the tacks. If I'm doing open surgery I use stitches."* [HCP: BRI0022, Lower GI, RDE]<br><br>*Extract 22: I will use transfascial sutures to place the mesh as well, and that acts partially as a fixation. I will do that and some stapling device as well, and the Sugarbaker is associated with its own unique stapling pattern, because of the way in which it overlies the bowel."* [HCP: BRI023, Lower GI, RDE]

Table 4: Short-list of data items used for development of the CIPHER Study case report forms

For peer review only

<table>
<tr><th colspan="3">1. Surgical approach to stoma formation</th></tr>
<tr><td rowspan="2">a. Context of stoma formation</td><td colspan="2">Indication for surgery (see separate sheet)</td></tr>
<tr><td colspan="2">Name of procedure (see separate sheet)</td></tr>
<tr><td rowspan="3">b. Intended type of access used</td><td colspan="2">Minimally invasive [SILS; Laparoscopic; Robotic]</td></tr>
<tr><td colspan="2">Open</td></tr>
<tr><td colspan="2">Trephine</td></tr>
<tr><td>c. Intended type of procedure converted to open</td><td colspan="2">Conversion from minimally invasive to open [SIL converted, Laparoscopic converted; Robotic converted] Y/N</td></tr>
<tr><td rowspan="6">d. Type of stoma formed</td><td colspan="2">Envisaged longevity of stoma [permanent, uncertain]</td></tr>
<tr><td colspan="2">End</td></tr>
<tr><td>Loop</td><td>With or without rod</td></tr>
<tr><td colspan="2">Loop end (Abcarian)</td></tr>
<tr><td colspan="2">Double barrelled</td></tr>
<tr><td colspan="2">Other</td></tr>
<tr><td rowspan="6">e. Section of bowel used to form functioning end of stoma</td><td colspan="2">Jejunum</td></tr>
<tr><td colspan="2">Ileum</td></tr>
<tr><td colspan="2">Ascending colon</td></tr>
<tr><td colspan="2">Transverse colon</td></tr>
<tr><td colspan="2">Descending colon</td></tr>
<tr><td colspan="2">Sigmoid colon</td></tr>
<tr><td rowspan="2">f. Stoma site</td><td rowspan="2">Stoma site pre-marked Y/N [preserved with suture, pen or not preserved]</td><td>Stoma site marked by [stoma nurse, surgeon, non-specialist nurse, other]</td></tr>
<tr><td>Stoma formed/ not formed at pre-marked site (Y/N)</td></tr>
<tr><td rowspan="2">g. Route of stoma</td><td colspan="2">Trans-peritoneal</td></tr>
<tr><td colspan="2">Extra-peritoneal (cannot be trephine in section b)</td></tr>
<tr><th colspan="3">2. Trephine formation</th></tr>
<tr><td>a. Subcutaneous tissue</td><td colspan="2">Subcutaneous tissue excised (Y/N)</td></tr>
<tr><td rowspan="3">b. Relationship of the muscle layer incision to the rectus abdominis</td><td colspan="2">Outside of the rectus sheath (Within oblique abdominal muscles)</td></tr>
<tr><td rowspan="2">Within the rectus sheath</td><td>Through the belly of the rectus abdominis</td></tr>
<tr><td>Lateral to the belly of the rectus abdominis* (e.g. LRAPS technique)</td></tr>
<tr><td rowspan="5">c. Anterior sheath (ONLY if within the rectus sheath*)</td><td colspan="2">Was a laparoscopic trocar used to puncture the anterior sheath (Y/N) (only for minimally invasive Laparoscopic + robotic OR converted Laparoscopic + robotic procedure) (NOT SILS or SILS converted)</td></tr>
<tr><td colspan="2">Size of incision [widest diameter in mm]</td></tr>
<tr><td colspan="2">Shape of incision [linear (horizontal, vertical), cruciate, circular, other]</td></tr>
<tr><td colspan="2">Was any of the anterior sheath removed? (Y/N)</td></tr>
<tr><td>Adjustments made to the size of the incision (Y/N)</td><td>Sutures used to buttress end of incision (Y/N)</td></tr>
</table>

For peer review only

**d. Posterior sheath *(ONLY within the rectus sheath*)***	Was a laparoscopic trocar used to puncture the anterior sheath (Y/N) *(only for minimally invasive Laparoscopic + robotic OR converted Laparoscopic + robotic procedure) (NOT SILS or SILS converted)*		
	Size of incision [widest diameter in mm]		
	Shape of incision [linear (horizontal, vertical), cruciate, circular, other]		
	Was any of the posterior sheath removed? (Y/N)		
	Adjustments made to the size of the incision (Y/N)		Sutures used to buttress end of incision (Y/N)
**e. Muscle fibres**	Separated with blunt dissection (Y/N) (*only possible if within rectus belly/oblique muscles)*		
**f. Intra-operative vessel damage**	Epigastric vessel (Y/N)		
**g. Laparoscopic procedures (*only for minimally invasive Laparoscopic + robotic OR converted Laparoscopic + robotic procedure) (NOT SILS)***	Location of trephine in relation to port site [Trephine created at port site/ Trephine created in a location other than port site]	Trephine created at port site	Trephine created at the port site at the beginning of procedure [then subsequently used as port site]
			Trephine created at end of procedure [conversion of port site to trephine]
**3. Reinforcing the stoma trephine with mesh**			
**a. Mesh type**	Mesh used (Y/N)		Product code, fix label, provide manufacture name
**b. Mesh size**	Mesh cut or adjusted (Y/N)		Size of mesh inserted if changed from original (Y/N) [height and width measured in cm OR if circular/oval diameter in cm]
			Shape of mesh if changed from original (Y/N) [3D/funnel; Circular/oval; Square/rectangular]
**c. Location of mesh placement**	Please indicate using diagram [Diagram provided with the following drop-down categories: Sublay/pre-peritoneal/retro-rectus; Underlay/intra-peritoneal; Onlay; Inlay] (Retrorectus; Intra-peritoneal; Onlay but keep all descriptions on the diagram)		
**d. Route used to position mesh**	Through the main operative incision (e.g. in an open procedure or an extended port site) *(any type of surgery) (can be all three: onlay, intraperitoneal or retorectus)*		
	Through the stoma trephine *(any type of surgery) (can be all three: onlay, intraperitoneal or retorectus)*		
	Via a port (*Any minimally invasive or converted procedure) (can only be intraperitoneal placement of mesh)*		
	Keyhole Y/N		What shape was the keyhole [cruciate; circular/oval; Slit]
			What was the size was the keyhole *(Maximum diameter)*

For peer review only

**e. Securing the mesh**	Mesh secured to abdominal wall *(including sheath, muscle, peritoneum)* (Y/N)
	Mesh secured to stoma serosa (Y/N)
**4. Use of the stoma as a specimen extraction site**	
**a. Stoma trephine used as a specimen extraction site**	Stoma trephine used as an extraction site (Y/N)
**5. Closure of other wounds formed during the procedure**	
**a. Closure of deep layers of the abdominal wall**	Main abdominal incision (e.g. in an open procedure, extended port site or specimen extraction site) [Small bite closure; Large bite closure; NA])
	Biggest port site (e.g. 10, 11 or 12mm) *(Minimally invasive laparoscopic + robotic OR converted laparoscopic + robotic procedures only)* [Closure of deep layer Y/N/NA]
**6. Spouting the stoma lumen**	
**a. Suturing of bowel to skin**	Has the stoma been spouted (Y/N)

For peer review only

CIPHER STUDY

The CIPHER Study

TH1

SURGICAL TECHNIQUE

Patient Initials: ☐☐☐

CIPHER Study ID: ☐☐☐-☐☐☐☐

SURGEON DETAILS

Most senior surgeon scrubbed in at time of stoma formation:

Name: ______________________________ GMC number: ______________

PATIENT DETAILS

Operation date: __ __ /__ __ /__ __ __ __ (d d / m m / y y y y)

Operation start time (defined as knife to skin: time): __ __ : __ __

Operation end time (defined as time of final skin suture in wound): __ __ : __ __

Month of birth: __ __ Year of birth: __ __ __ __

Patient's Sex: *Male* ☐ *Female* ☐

SURGICAL APPROACH TO STOMA FORMATION

Indication for surgery (***please select one option***):

- Tumour – benign ☐
- Tumour – malignant ☐
- Diverticular Disease ☐
- Functional Intestinal Disorder ☐
- Inflammatory Bowel Disease (IBD) ☐
- IBD – Crohn's ☐
- IBD – Ulcerative Colitis ☐
- Other ☐

If other, specify: ______________________________

ASA Classification:

- I ☐
- II ☐
- III ☐
- IV ☐
- V ☐

For peer review only

BMJ Open

CIPHER STUDY

The CIPHER Study

SURGICAL TECHNIQUE

TH1

Patient Initials: ☐☐☐

CIPHER Study ID: ☐☐☐-☐☐☐☐

Name of procedures (tick YES or NO as appropriate for each):

	Yes	*No*
Small bowel resection	☐	☐
Colectomy: left (including sigmoid colectomy and anterior resection)	☐	☐
Colectomy: right (including ileocaecal resection)	☐	☐
Colectomy: subtotal or panproctocolectomy	☐	☐
Hartmann's procedure	☐	☐
Colorectal resection – other	☐	☐
Reduction of volvulus	☐	☐
Stricturoplasty	☐	☐
Drainage of abscess/collection	☐	☐
Debridement	☐	☐
Abdominoperineal excision	☐	☐
Posterior pelvic exenteration	☐	☐
Repair or revision of anastomosis	☐	☐
Repair of intestinal fistula	☐	☐
Resection of other intra-abdominal tumour(s)	☐	☐
Stoma formation	☐	☐
Other	☐	☐

If other, specify: ______________________________

If **YES** to ***abdominoperineal excision*** or ***posterior pelvic exenteration***:

Vertical rectus abdominis myocutaneous (VRAM) flap *Yes* ☐ *No* ☐

Intended type of access used (***please select one***):

- SLS ☐
- Laparoscopic ☐
- Robotic ☐
- Open ☐
- Trephine ☐

Intended type of procedure converted to open: *Yes* ☐ *No* ☐
***(Do not answer if intended type of access* was open)**

For peer review only - http://bmjopen.bmj.com/site/about/guidelines.xhtml

The CIPHER Study

TH1

SURGICAL TECHNIQUE

Patient Initials: ☐☐☐

CIPHER Study ID: ☐☐☐-☐☐☐☐

Envisaged longevity of stoma: Permanent ☐ Uncertain ☐

Type of stoma formed (***please select one***):

- End ☐
- Loop ☐ → If **loop**, with or without rod: With ☐ Without ☐
- Loop end ☐
- Double barrelled ☐
- Other ☐

Section of bowel used to form functioning end of stoma (***please select one***):

- Jejunum ☐
- Ileum ☐
- Ascending colon ☐
- Transverse colon ☐
- Descending colon ☐
- Sigmoid colon ☐

Stoma site pre-marked (***please select one***):

- Not preserved ☐
- Preserved with suture ☐ →
- Pre-marked with pen ☐ →

If ***preserved with suture*** or ***pre-marked with pen***,
Stoma site marked by (***please select one***):

- Stoma nurse ☐
- Surgeon ☐
- Non-specialist nurse ☐
- Other ☐

If other, specify: _______________

	Yes	*No*
Stoma formed at pre-marked site:	☐	☐

Route of stoma: Trans-peritoneal ☐ Extra-peritoneal ☐

For peer review only

The CIPHER Study

SURGICAL TECHNIQUE

TH1

Patient Initials: ☐☐☐

CIPHER Study ID: ☐☐☐-☐☐☐☐

TREPHINE FORMATION

Subcutaneous tissue excised: *Yes* ☐ *No* ☐

Relationship of the muscle layer incision to the rectus abdominis (***please select one***):

- Outside of the rectus sheath (within oblique abdominal muscles) ☐ → **Continue on next page**
- Within the rectus sheath - though the belly of the rectus abdominis ☐
- Within the rectus sheath - lateral to the belly of the rectus abdominis ☐

→ Complete the box below

Anterior sheath: was a laparoscopic trocar used to puncture the anterior sheath *Yes* ☐ *No* ☐
(Only answer for laparoscopic or robotic procedures)

Anterior sheath: size of incision [widest diameter in mm] ___________

Anterior sheath: Shape of incision (***please select one***)

- Linear - horizontal — ☐
- Linear - vertical | ☐
- Cruciate + ☐
- Circular ○ ☐
- Other ? ☐

Anterior sheath: was any of the anterior sheath removed *Yes* ☐ *No* ☐

Anterior sheath: adjustments made to the size of the incision *Yes* ☐ *No* ☐

Posterior sheath: was a laparoscopic trocar used to puncture the posterior sheath *Yes* ☐ *No* ☐
(Only answer for laparoscopic or robotic procedures)

Posterior sheath: size of incision [widest diameter in mm] ___________

Posterior sheath: Shape of incision (***please select one***)

- Linear - horizontal — ☐
- Linear - vertical | ☐
- Cruciate + ☐
- Circular ○ ☐
- Other ? ☐

Posterior sheath: was any of the posterior sheath removed *Yes* ☐ *No* ☐

Posterior sheath: adjustments made to the size of the incision *Yes* ☐ *No* ☐

For peer review only - http://bmjopen.bmj.com/site/about/guidelines.xhtml

The CIPHER Study

TH1

SURGICAL TECHNIQUE

Patient Initials: ☐☐☐

CIPHER Study ID: ☐☐☐-☐☐☐☐

Muscle fibres separated with blunt dissection *Yes* ☐ *No* ☐

Intra-operative vessel damage - epigastric vessel *Yes* ☐ *No* ☐

Location of trephine in relation to port site (***please select one***):
(Only answer for laparoscopic or robotic procedures, including procedures converted to open)

Trephine created at the port site as the beginning of procedure (then subsequently used as port site) ☐

Trephine created at end of procedure (conversion of port site to trephine) ☐

Trephine created in a location other than port site ☐

Were sutures used to buttress the incision (***please select one***):
(Only answer if relationship of the muscle layer incision to the rectus abdominis is "within rectus sheath")

Anterior sheath only ☐

Posterior sheath only ☐

Both anterior and posterior sheath ☐

Anterior and posterior sheath sutured together ☐

No ☐

For peer review only

The CIPHER Study

SURGICAL TECHNIQUE

TH1

Patient Initials: ☐☐☐

CIPHER Study ID: ☐☐☐-☐☐☐☐

REINFORCING THE STOMA TREPHINE WITH MESH

Was mesh used to reinforce the stoma trephine *Yes* ☐ *No* ☐ → If **NO**, continue on next page

If **YES**, complete the box below

Mesh product code (attach product sticker) ***Attach product sticker here***

Mesh cut or adjusted *Yes* ☐ *No* ☐

If **YES**,

Craniocaudal length of mesh inserted if changed from original [in cm] ______________

Medio-lateral length of mesh inserted if changed from original [in cm] ______________

Diameter of mesh inserted if changed from original [in cm] ______________

Shape of mesh inserted if changes from original (***please select one***):

3D/funnel ☐

Circular/Oval ☐

Square/rectangular ☐

No change in shape ☐

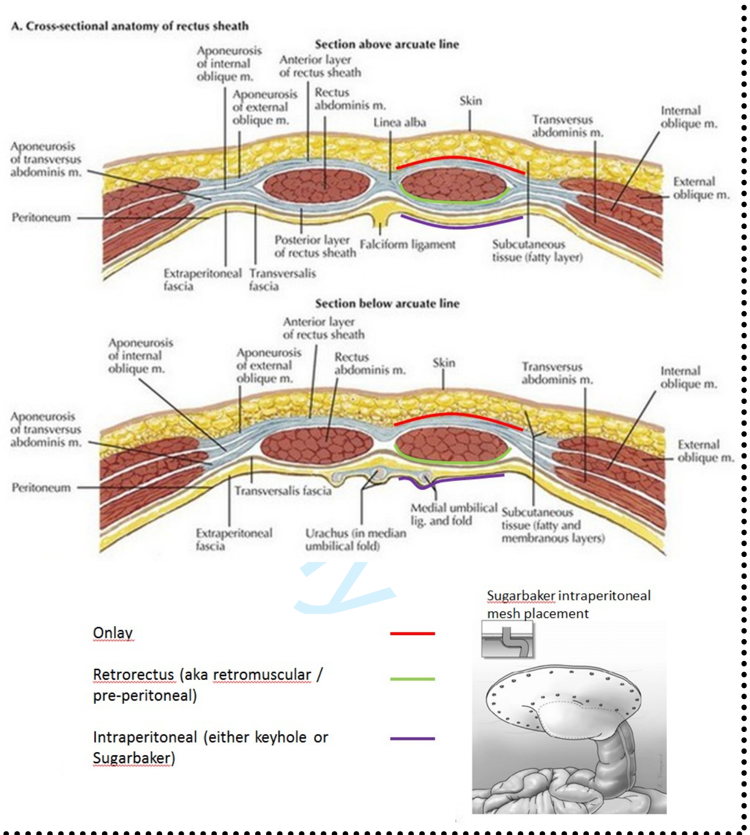

Location of mesh replacement (***please select one***):

Sublay/pre-peritoneal/retro-rectus ☐

Underlay/intra-peritoneal ☐

Onlay ☐

Route used to position mesh (***please select one***):

Through the main operative incision ☐

Through the stoma trephine ☐

Via a port ☐

What shape was the hole in the mesh?

Cruciate ☐ Circular/oval ☐ Slit ☐ None (Sugarbaker) ☐

What size was the hole in the mesh [in mm] __________

Mesh secured to abdominal wall (including sheath, muscle, peritoneum) *Yes* ☐ *No* ☐

Mesh secured to stoma serosa *Yes* ☐ *No* ☐

For peer review only

For peer review only - http://bmjopen.bmj.com/site/about/guidelines.xhtml

TH1

SURGICAL TECHNIQUE

Patient Initials: ☐☐☐

CIPHER Study ID: ☐☐☐-☐☐☐☐

USE OF THE STOMA TREPHINE AS A SPECIMEN EXTRACTION SITE

Stoma trephine used as an extraction site *Yes* ☐ *No* ☐

CLOSURE OF OTHER WOUNDS FORMED DURING THE PROCEDURE

Main abdominal incision (***please select one***):

Small bite closure ☐

Large bite closure ☐

N/A ☐

Biggest port size [in mm] ____________

(Only answer for laparoscopic or robotic procedures, including procedures converted to open)

Closure of deep layer *Yes* ☐ *No* ☐

(Only answer for laparoscopic or robotic procedures, including proce- dures con- verted to open)

SPOUTING THE STOMA LUMEN

Has the stoma been spouted *Yes* ☐ *No* ☐

COMMENTS

____

____

____

____

Who has collated this data?

GMC/NMC number: ______________________________

Date completed: __ __ / __ __ /__ __ __ __

For peer review only

Standards for Reporting Qualitative Research (SRQR)*

http://www.equator-network.org/reporting-guidelines/srqr/

Title and abstract

	Page/line no(s).
**Title** - Concise description of the nature and topic of the study Identifying the study as qualitative or indicating the approach (e.g., ethnography, grounded theory) or data collection methods (e.g., interview, focus group) is recommended	Page 1, line 1
**Abstract** - Summary of key elements of the study using the abstract format of the intended publication; typically includes background, purpose, methods, results, and conclusions	Page 2, line 3

Introduction

**Problem formulation** - Description and significance of the problem/phenomenon studied; review of relevant theory and empirical work; problem statement	Page 4, line 2
**Purpose or research question** - Purpose of the study and specific objectives or questions	Page 4, line 14

Methods

**Qualitative approach and research paradigm** - Qualitative approach (e.g., ethnography, grounded theory, case study, phenomenology, narrative research) and guiding theory if appropriate; identifying the research paradigm (e.g., postpositivist, constructivist/ interpretivist) is also recommended; rationale**	Page 6, line 29
**Researcher characteristics and reflexivity** - Researchers' characteristics that may influence the research, including personal attributes, qualifications/experience, relationship with participants, assumptions, and/or presuppositions; potential or actual interaction between researchers' characteristics and the research questions, approach, methods, results, and/or transferability	Page 7, line 18<br>Page 12, line 6<br>Page 25, line 16
**Context** - Setting/site and salient contextual factors; rationale**	Page 7, line 30
**Sampling strategy** - How and why research participants, documents, or events were selected; criteria for deciding when no further sampling was necessary (e.g., sampling saturation); rationale**	Page 6, line 15<br>Page 7, line 30
**Ethical issues pertaining to human subjects** - Documentation of approval by an appropriate ethics review board and participant consent, or explanation for lack thereof; other confidentiality and data security issues	Page 6, line 24
**Data collection methods** - Types of data collected; details of data collection procedures including (as appropriate) start and stop dates of data collection and analysis, iterative process, triangulation of sources/methods, and modification of procedures in response to evolving study findings; rationale**	Page 7, line 18

For peer review only - http://bmjopen.bmj.com/site/about/guidelines.xhtml

**Data collection instruments and technologies** - Description of instruments (e.g., interview guides, questionnaires) and devices (e.g., audio recorders) used for data collection; if/how the instrument(s) changed over the course of the study	Page 7, line 18<br>Page 7, line 25
**Units of study** - Number and relevant characteristics of participants, documents, or events included in the study; level of participation (could be reported in results)	Page 12, line 2
**Data processing** - Methods for processing data prior to and during analysis, including transcription, data entry, data management and security, verification of data integrity, data coding, and anonymization/de-identification of excerpts	Page 8, line 2
**Data analysis** - Process by which inferences, themes, etc., were identified and developed, including the researchers involved in data analysis; usually references a specific paradigm or approach; rationale**	Page 8, line 9
**Techniques to enhance trustworthiness** - Techniques to enhance trustworthiness and credibility of data analysis (e.g., member checking, audit trail, triangulation); rationale**	Page 6, line 18<br>Page 8, line 4<br>Page 8, line 29

Results/findings

**Synthesis and interpretation** - Main findings (e.g., interpretations, inferences, and themes); might include development of a theory or model, or integration with prior research or theory	Page 12, line 12<br>Page 13, line 20
**Links to empirical data** - Evidence (e.g., quotes, field notes, text excerpts, photographs) to substantiate analytic findings	Page 14, line 4 –<br>Page 21, line 9

Discussion

**Integration with prior work, implications, transferability, and contribution(s) to the field -** Short summary of main findings; explanation of how findings and conclusions connect to, support, elaborate on, or challenge conclusions of earlier scholarship; discussion of scope of application/generalizability; identification of unique contribution(s) to scholarship in a discipline or field	Page 23, line 22<br>- Page 24, line 8<br>Page 25, line 14<br>-<br>Page 26, line 11
**Limitations** - Trustworthiness and limitations of findings	Page 24, line 9 -<br>Page 25, line 13

Other

**Conflicts of interest** - Potential sources of influence or perceived influence on study conduct and conclusions; how these were managed	Page 26, line 30
**Funding** - Sources of funding and other support; role of funders in data collection, interpretation, and reporting	Page 26, line 18

*The authors created the SRQR by searching the literature to identify guidelines, reporting standards, and critical appraisal criteria for qualitative research; reviewing the reference lists of retrieved sources; and contacting experts to gain feedback. The SRQR aims to improve the transparency of all aspects of qualitative research by providing clear standards for reporting qualitative research.

For peer review only - http://bmjopen.bmj.com/site/about/guidelines.xhtml

**The rationale should briefly discuss the justification for choosing that theory, approach, method, or technique rather than other options available, the assumptions and limitations implicit in those choices, and how those choices influence study conclusions and transferability. As appropriate, the rationale for several items might be discussed together.

Reference:

O'Brien BC, Harris IB, Beckman TJ, Reed DA, Cook DA. **Standards for reporting qualitative research: a synthesis of recommendations.** *Academic Medicine*, Vol. 89, No. 9 / Sept 2014 DOI: 10.1097/ACM.0000000000000388

For peer review only
